# New York State Climate Impacts Assessment Chapter 09: Transportation

**DOI:** 10.1111/nyas.15198

**Published:** 2024-12-09

**Authors:** Amy Macdonald, Joan McDonald, Thomas Schmitt, Hua Cai, Jennifer Ceponis, Cheila Cullen, Projjal Dutta, Andrea Cristina Ruiz, Amanda Stevens

**Affiliations:** ^1^ Ripple Resilience New York New York USA; ^2^ Westchester County White Plains New York USA; ^3^ Eastern Research Group, Inc. Golden Colorado USA; ^4^ Department of Environmental and Ecological Engineering Purdue University West Lafayette Indiana USA; ^5^ Capital District Transportation Committee Albany New York USA; ^6^ Department of Chemistry, Earth and Environmental Sciences City University of New York New York New York USA; ^7^ New York Metropolitan Transportation Authority New York New York USA; ^8^ Vanderbilt University Nashville Tennessee USA; ^9^ New York State Energy Research and Development Authority Albany New York USA

**Keywords:** adaptation, climate change, impacts, infrastructure, New York State, resilience, transportation, vulnerability

## Abstract

The transportation sector's functionality depends on climate conditions. Many New York State residents, communities, businesses, and institutions have already experienced transportation‐related climate change impacts, and many more will experience impacts in the future. This chapter examines climate change impacts on the various modes of transportation in New York State. It also highlights climate‐driven vulnerabilities among the state's communities, workers, regions, and businesses, with particular attention paid to issues of equity and environmental justice.

## 1 TECHNICAL WORKGROUP KEY FINDINGS

The transportation sector's functionality depends on climate conditions. Many New York State residents, communities, businesses, and institutions have already experienced transportation‐related climate change impacts, and many more will experience impacts in the future. This chapter examines climate change impacts on the various modes of transportation in New York State. It also highlights climate‐driven vulnerabilities among the state's communities, workers, regions, and businesses, with particular attention paid to issues of equity and environmental justice.

**Key Finding 1: Many climate hazards could damage or disrupt New York State's transportation systems, with heavy precipitation and sea level rise presenting the most severe risks**. All modes of transportation are vulnerable to increased inland flooding driven by more intense storms or increased coastal flooding driven by sea level rise and storm surge. These events can block vital routes, impede the safe operation of vehicles, damage infrastructure, and accelerate long‐term infrastructure deterioration. Transportation agencies have begun to respond to this threat with a combination of “gray” infrastructure, like elevating structures and creating barriers to keep floodwaters out of tunnels, and “green” infrastructure that uses natural solutions to absorb or drain excess water.

**Key Finding 2: New York State's transportation systems are vulnerable to impacts that cascade across modes of transport and across sectors**. The intermodal and cross‐sectoral nature of the state's transportation network increases the potential for cascading impacts, which can affect physical infrastructure, user accessibility, and supply chains. For example, power outages or floods that disrupt mass transit or intermodal hubs can create a surge of demand in other modes, increasing congestion and delays. Solutions include not only making individual modes more resilient, but also ensuring the availability of reliable backup modes to keep New York moving.

**Key Finding 3: Climate change could exacerbate existing transportation system inequities, which well‐planned climate solutions can help to reduce**. The construction of some transportation infrastructure such as highways or transit lines has created disproportionate burdens, such as highways that severed historically Black neighborhoods decades ago or “transit deserts” that leave some communities with few transportation options. Climate change could amplify inequities as people with higher dependence on certain kinds of transportation or with limited mobility options lack alternatives if their primary mode is disrupted. Investments in climate adaptation and resilience present opportunities to improve transportation access and mobility while addressing the consequences of past decisions.

**Key Finding 4: Transportation investments increasingly seek to address climate change impacts, but decision‐makers still face constraints that limit their ability to respond proactively**. As climate impacts on the transportation system become more apparent, many transportation agencies in New York State have taken steps to design for the climate of the future, not the climate of the past, and embrace new techniques and technologies. Still, decision‐makers must contend with rising costs, financial constraints, limited personnel capacity (particularly in smaller or less well‐funded jurisdictions), and a backlog of routine maintenance needs and infrastructure in disrepair. Sustaining proactive investments requires transportation agencies and departments to balance their resource commitments against the potential costs of inaction.

**Key Finding 5: Advancements in transportation planning, systems, and mobility present both opportunities and challenges for climate adaptation**. Electric vehicles (EVs), on‐demand mobility options, intelligent systems, and advanced materials are among the many advancements fostering the evolution of the transportation sector. While these advancements may present opportunities for enhanced mobility and environmental benefits, they can also exacerbate vulnerabilities, such as increasing dependence on the electric power grid. Planners must consider how climate change will affect the transportation systems of tomorrow, not just the systems of today.

BOX 1 **Developments since the 2011 ClimAID assessment**

This chapter expands the scope of climate change impacts on the transportation sector compared with the 2011 ClimAID assessment. It includes a larger suite of topics, such as micromobility, intermodal connections, human and transportation system interactions, and emerging adaptation strategies and technologies. Whereas ClimAID largely focused on vulnerabilities and climate impacts of physical systems organized by climate hazards and risks, this chapter considers impacts on a modal basis.

The current assessment gives expanded attention to groups of people who face particular vulnerabilities and disproportionate impacts, including underserved and overburdened communities, Indigenous Peoples, urban and rural communities, and transportation sector employees. This chapter also expands on resilience and adaptation strategies and opportunities within the transportation sector while focusing on equity as a key element of sustainable climate change response.

Climate resilience practices have continued to advance and become more widely adopted, particularly in response to extreme events like Superstorm Sandy. This chapter addresses such events and speaks to the changes in modal technologies, transportation investments, and resilience and adaptation strategies that have occurred since the 2011 ClimAID assessment.

## 2 INTRODUCTION AND BACKGROUND

The impacts of climate change affect all transportation modes in New York State and will continue to affect both the physical infrastructure and social aspects of transportation usage. This chapter discusses climate‐related impacts on the transportation sector, projections of future climate changes and impacts, and opportunities to adapt and build resilience. It cites evidence from technical and peer‐reviewed literature, anecdotal sources, and recent climate models, as well as from direct engagement with a range of sector experts and stakeholders.

The cross‐sectoral nature of climate change impacts (and of the transportation system itself) creates challenges when assessing impacts to this sector. This chapter reduces cross‐sectoral complexity by (1) segmenting the transportation sector by mode and (2) highlighting the climate hazards and stressors that directly impact each mode. This organization can help readers easily comprehend the impacts to specific modes while also recognizing cross‐sectoral and intermodal connections. The chapter is organized as follows:

- **Sections 3 through 8** discuss climate impacts for six transportation modes: roads and highways, mass transit, rail transportation, maritime transportation, air transportation, and micromobility. Each section provides background information on the mode, including scope; context within New York State; and social, economic, and usage trends. Each section then discusses observed and projected impacts, people and systems at particular risk, additional factors and concerns such as the role of local and state governments and emerging technologies, and adaptation and resilience strategies. Each section also discusses intermodal connections and dependencies, consistent with the growing recognition that a “siloed” approach to transportation modes could overlook or understate important impacts, particularly as impacts compound and cascade.^1^ Many examples are drawn from the New York City Metropolitan Area because of its confluence of many modes, its large population, and the hazards associated with its coastal location. However, much of the discussion is equally applicable to transportation systems in other parts of the state.
  Note that each adaptation and resilience discussion intentionally focuses on a few notable strategies or examples, as it would be beyond the scope of this assessment to provide a comprehensive catalog of adaptation measures in use or potentially applicable in New York State. Thus, the results of this assessment should not be construed as a quantitative characterization of the state of transportation adaptation in New York or any corresponding “adaptation deficit.”
- **Section** **9** looks at opportunities for positive change that can grow out of climate adaptation efforts and identifies emerging topics and research needs. This section also provides a conclusion, summarizing the major findings and recommendations presented in the chapter.
- The Traceable Accounts appendix examines each key finding in depth. It provides citations that support each assertion, and it presents the authors’ assessment of confidence in each finding.
- **Case studies** highlight the climate impacts farmers and communities are experiencing firsthand as well as the adaptation and resilience strategies they are employing to minimize the effects on their livelihoods. These case studies are not included in the chapter proper but are available through links provided in the chapter.

### 2.1 Sector scope and context

New York's bustling transportation sector is rooted in the state's unique geography, population base, and robust economy. The state has long prospered from its access to natural waterways like the Atlantic Ocean (via New York Harbor and the St. Lawrence Seaway), as well as from massive infrastructure projects for transporting agricultural and manufactured goods, such as the Erie Canal. In the 19th century, innovation around railways and locomotion allowed for unprecedented connection of people and products throughout the state.^2^ Centuries of transportation projects, as well as population and economic growth, have created one of the largest, most complex transportation networks and supporting workforces in the United States.

New York City was also a pioneer of mass transit, from its adoption of the first horse‐drawn streetcars in America to the opening of its famous subway. Due largely to the prevalence of mass transit in the New York City Metropolitan Area, state residents use mass transit at the highest rate in the country.^3^ This heavy use of public transit contributes to New York also having the lowest per‐capita greenhouse gas emissions in the United States.^4^ Figure 9‐1 shows this relatively heavy use of mass transit, along with the many other modes in which New Yorkers commute to work. (Note that elsewhere in the chapter, some of the statistics available at the time of writing predated the COVID‐19 pandemic. The pandemic changed certain transportation usage patterns in substantial ways, and some of these patterns have not returned to their prepandemic state.)

**FIGURE 9‐1:**
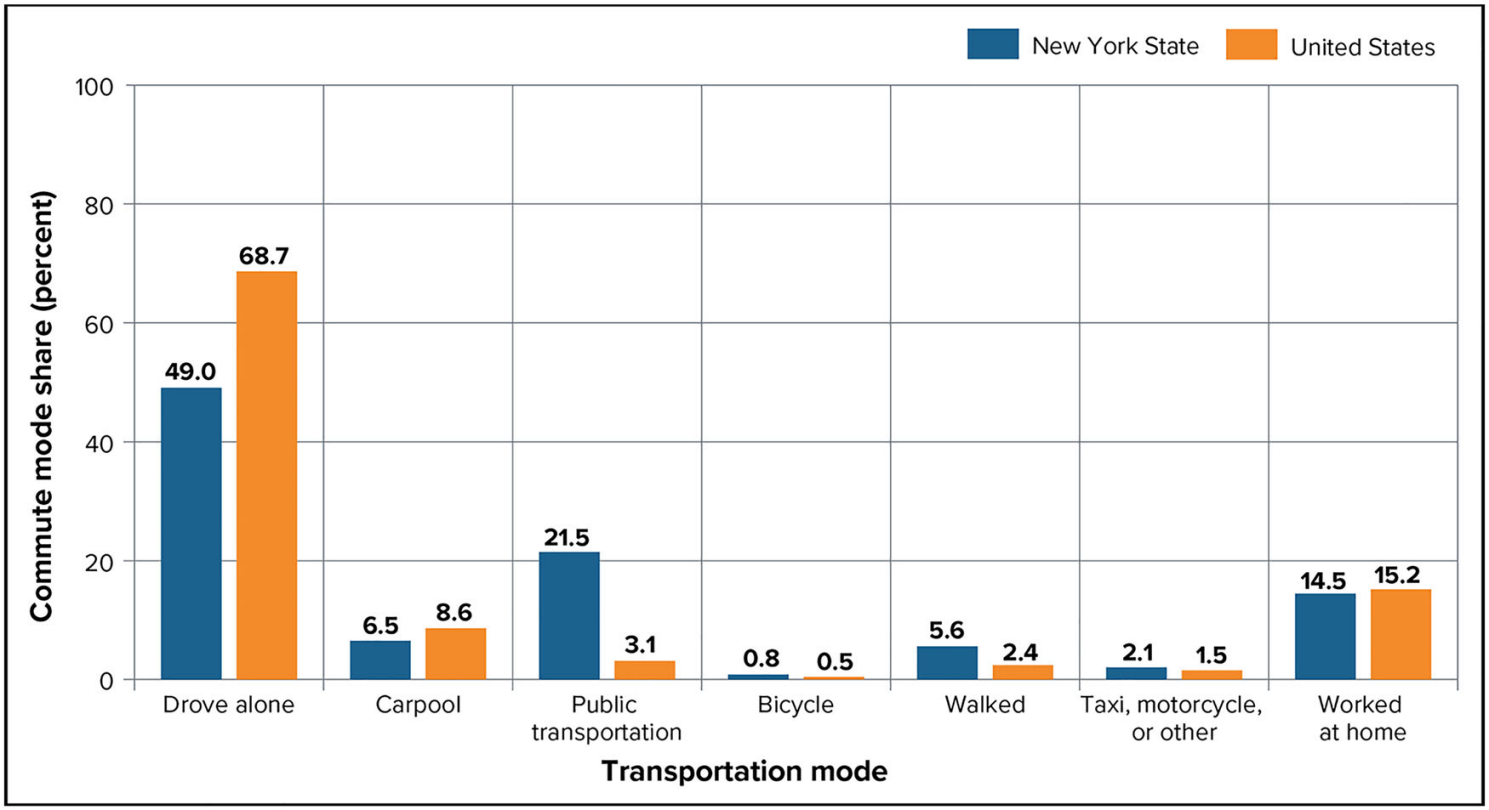
Prevalence of transportation modes for commuters in New York State, 2022. The graph shows percentages of workers aged 16 and older. Data from U.S. Department of Transportation (n.d.).^3^

New York State's robust multimodal transportation system is vital to the state's continued development and prosperity, as it facilitates access to jobs and essential services, efficient movement of goods, and connections to the global economy.

Transportation in the state is evolving in response to social, economic, and environmental trends. New planning strategies and new technologies, such as EVs and on‐demand services, are emerging as part of this evolution. So is micromobility: transportation by means of small, lightweight, user‐operated devices, such as bikes, scooters, and skateboards. While many of these technologies and strategies will result in positive change (e.g., lower greenhouse gas emissions, greater efficiency), they can also present new complications as the climate changes.

### 2.2 Key climate hazards

The integrity and functionality of New York's transportation systems are sensitive to climate conditions. For consistency, this assessment uses the term “climate hazard” to describe such conditions, including some that are inherently hazardous and others, like precipitation, that can create a hazard under certain circumstances (too much, too little, bad timing). Key climate hazards include:

- **Precipitation and flooding**. Increased precipitation can disrupt the flow of traffic and cause temporary blockages or backups. Flooding and ground saturation can damage fixed infrastructure, such as roads, bridges, runways, subways, and rails. Snow and ice can cause road and railway closures and disrupt mass transit and air travel.
- **Severe storms**. New Yorkers have already seen how extreme weather events can disrupt transportation. For example, Superstorm Sandy in 2012 and Hurricane Ida in 2021 caused deaths and catastrophic damage; images of flooded subway tunnels and cars stranded on flooded streets and rural highways are likely fresh in many residents’ memories. Record‐breaking lake‐effect snowstorms in recent years have impeded mobility in Western New York. While uncertainty remains as to how the frequency of storms will change under future climate conditions, several types of storms are projected to become more intense.^5^ Projections also suggest that lake‐effect snow could increase over the next few decades despite a projected decrease in statewide snowfall, although increasing air temperatures mean that more of this precipitation may fall as rain than as snow by the end of the century.^5^ While these extreme weather events cannot be attributed entirely to climate change, they act as examples of the disruption and damage to the transportation system that are projected to accompany potential future climate conditions.
- **Sea level rise and storm surge**. Sea level is projected to rise along New York State's coastline by up to 1 foot by the 2030s, about 2−3 feet by the 2080s, and more than 4 feet by 2150, relative to a 1995−2014 baseline, as discussed in more detail in New York State's Changing Climate.^5^ Over time, the rise of saline ocean waters could corrode transportation infrastructure and erode the land supporting these systems along the state's Atlantic coast and up the Hudson River. Higher sea levels will also create drainage challenges, potentially backing up stormwater runoff onto streets and highways.^6^ Coastal storms with associated storm surge will threaten coastal transportation infrastructure and the people who use it, especially as a higher baseline sea level allows storm surge to rise higher and penetrate farther inland.^5^
- **Extreme temperatures**. Extreme temperatures and extreme temperature variability pose dangers to infrastructure, people, and goods in transit. More frequent and intense periods of extreme heat can damage or weaken infrastructure over the long term, while also presenting immediate threats to the health and safety of travelers and transportation workers. This chapter explores the infrastructure side of this impact in more depth, while the Human Health and Safety chapter addresses the human health dimensions.
- **Wildfires**. Although New York's wildfire risk is projected to remain relatively low compared with other parts of North America, the risk of damaging wildfires nonetheless exists, especially if seasonal drought accompanies the projected increase in average and extreme temperatures.^5^ Smoke from wildfires burning elsewhere can also affect transportation, as the state experienced in June 2023 from wildfires in Canada.^7^ Hazardous air quality can reduce mobility options if travelers must stay indoors, impede transportation services and projects by limiting the time workers can safely work outdoors, and reduce visibility for air travel. In June 2023, many flights in New York were delayed due to low visibility from wildfire smoke.^8^

Climate sensitivities can compound across modes of transportation and across other sectors. For example, extreme heat and severe storms can increase the risk of power outages, which cause service disruptions in electrified mass transit, EV charging, and the powered infrastructure supporting other modes. (Refer to the Energy chapter for more discussion of power grid impacts.)

Current transportation systems were designed based on assumptions about local and regional weather and climate, as well as observed patterns and data. Looking ahead, planners and engineers will need to consider new assumptions and design criteria that account for increased uncertainty in models, changes in historical norms, and more frequent and intense extreme events—all of which increase the stress on transportation systems.

### 2.3 Nonclimate factors

A wide range of nonclimate factors could increase or decrease the risks that climate hazards pose to New York's transportation sector. Nonclimate factors include conditions or features inherent in the physical transportation system as well as societal influences related to human behavior or governance.

- **Density**. While infrastructure throughout the state faces climate risks, the potential impacts are magnified in areas with a high concentration of infrastructure and users, like New York City. A climate event that has a relatively small geographic footprint can still affect many people and create large economic costs when it hits a densely populated area. Moreover, in communities that are largely built out, it may be harder to reduce hazard exposure (e.g., urban flooding where upsizing stormwater management capacity is challenging) or replace infrastructure that requires different installation characteristics.
- **Deterioration**. Each mode is subject to infrastructure deterioration over time. Changing climate conditions can stress transportation infrastructure components, speeding deterioration and shortening expected lifespans. Replacing infrastructure earlier than planned will increase costs to society.
- **Funding and staffing**. Transportation infrastructure requires ongoing societal investment to construct and maintain. A lack of funding or staff could constrain investment in climate‐resilient transportation projects, as well as routine maintenance that also promotes operational resilience. Conversely, massive public infrastructure investments and carbon‐reduction policies can create substantial opportunities for resilience and adaptation planning in transportation.
- **Training, knowledge, and data access**. Literature, data, and training on proven adaptation and resilience techniques are available, but several transportation professionals who provided input to the assessment team noted that these resources can sometimes be difficult for practitioners to identify, locate, and understand. Local governments are responsible for maintaining many aspects of the state's transportation system, and some might lack the resources to learn about and deploy these new techniques.
- **Public opinion**. Public support is critical for capital improvements to transportation. Proposed projects could fail if planners do not take public sentiment into account—for example, ignoring the voices of adjacent communities or failing to consider how equitably the benefits and costs of the project will be distributed.
- **Systemic change in modes and fuels**. It is important to assess the impacts of climate change not just on the state's transportation systems as they look today, but also on the systems as they might look in the decades ahead. As government policies and financial incentives drive electrification and the goal of “net‐zero” greenhouse gas emissions, New York State's transportation systems will become increasingly dependent on the reliability of the electric power grid. The power grid, in turn, faces its own challenges associated with projected increases in the frequency or intensity of extreme events such as heat waves and severe storms. The Energy chapter explores these challenges while addressing a similar dynamic with a system in flux; that chapter considers future impacts of climate change on a power grid that will eventually depend more on renewable sources and need to satisfy demand from increasingly electrified buildings and transportation systems. For transportation systems, increased dependence on electric power creates opportunities to reduce greenhouse gas emissions and local air pollution, but also increases the risk of grid‐related climate impacts and less flexibility if backup modes also depend on electricity.
- **Technology**. New technologies, such as EVs, rideshare apps, and micromobility options, are changing how people choose to travel. Intelligent transportation systems and connected and autonomous vehicles are changing what is possible in terms of transportation system efficiency and safety. New materials and engineering techniques could make some infrastructure more resilient. These developments will affect transportation planning as practitioners seek to incorporate technological benefits. However, new technologies could be difficult to test against less predictable long‐term climate factors and could, therefore, present unexpected climate risks and vulnerabilities.
- **Human behavior and population changes**. Changes in the way people travel can affect transportation investment in specific areas. For example, the broad emergence of the ability to work remotely during and after the COVID‐19 pandemic has changed transit ridership and commuting patterns.^3^, ^9^ Population growth or shrinkage, demographics, and migration patterns can also affect transportation use investment priorities.

### 2.4 Equity and climate justice

Transportation systems—and the mobility and economic activity they support—are broadly understood as a public good, necessary for a functioning society. However, access to mobility is unequal. People with less access to affordable, reliable transportation options will likely have access to fewer job opportunities and face longer travel times to necessary services like grocery stores and medical facilities. Climate change could reinforce or exacerbate these inequities, and so could responses to climate change, if not planned with equity in mind. Conversely, as noted in Section 2.6, responding to climate change also presents opportunities to reduce disparities.

Examples throughout this chapter illustrate how more frequent transportation system disruptions, such as those experienced during events like Superstorm Sandy, could disproportionately affect communities that already face heavy social and economic burdens. There are several general categories of disparities at the nexus of transportation and climate, including the following:

- **Dependence on transportation for physical safety during a climate event**. As the Assessment Introduction and the Buildings chapter explain in more detail, a legacy of racial segregation (e.g., redlining) and other forms of socioeconomic marginalization have forced vulnerable populations into higher‐risk geographies and lower‐quality housing, which increases their exposure to climate‐related hazards.^10^ Areas at risk include low‐lying urban neighborhoods and rural floodplains. People living in these areas will need transportation to evacuate during a climate‐related event such as a flood or a severe coastal storm. Also, people without access to cooling at home could require transportation to get to a cooling center during a heat wave. As the Human Health and Safety chapter explains, low‐income New Yorkers are less likely to have air conditioning.
- **Dependence on transportation for employment**. Lower‐wage earners are more likely to need to physically commute to work.^11^ Thus, they could be more affected by climate‐related mobility disruptions than higher‐wage earners who are more likely to have remote work options.
- **Limited transportation options**. A lack of backup options can leave people stranded if their primary mode of transportation is disrupted by a climate event. This is both a rural and an urban challenge. In rural areas, including around Tribal lands, historically underserved areas can be particularly affected by the closure of limited roads and bridges that connect populations to critical lifelines. In New York State's urban areas, many people depend on public transit, which can be cut off due to events such as flooding and power outages.
- **Impacts on people with disabilities**. People with disabilities can be at particular risk if they cannot travel to reach safe shelter, cooling centers, or critical services. In New York City, the Metropolitan Transportation Authority (MTA) is working with city, state, and federal authorities to improve transit access for people with disabilities, but the challenge remains large. As of 2021, approximately 75% of city subway stations did not comply with the Americans with Disabilities Act.^12^
- **Unequal availability of clean transportation technologies**. People with lower income may be less likely to benefit from emerging technologies in transportation, such as EVs, due to their high initial cost; market distribution that targets high‐income consumers; and government subsidies, like tax credits, that are only useful for households with sizable tax bills.

### 2.5 Indigenous communities

Ten territories held by federally or state recognized Tribal Nations share boundaries with New York State, totaling approximately 137 square miles.^13^ Roadways serve as the primary means of transportation into, out of, and throughout these areas.

State‐level statistics are limited, but national data illustrate a few transportation‐related challenges that Indigenous communities often face. One challenge is funding. Nationally, while state governments spend between $4000 and $5000 per mile on state road and highway maintenance, less than $500 per mile is spent on Tribal country road maintenance.^14^ Poorly maintained roads could be less safe for users and more prone to damage from climate events. A lack of pedestrian facilities, lighting, or traffic control devices can also affect safety.^15^ Studies have shown that nationally, Indigenous Peoples are almost five times more likely than white people to be killed while walking.^16^

Within New York, Indigenous communities have been subjected to transportation systems that have been imposed on them. For example, when a new exit was developed off Interstate 87 between 2019 and 2020, it was built over land considered sacred to three Indigenous Tribes: Stockbridge‐Munsee, Saint Regis Mohawk, and Delaware.^17^ Over a longer time frame, the Seneca Nation and New York State have faced disagreement over a portion of the New York State Thruway (Interstate 90) that the state built through the Cattaraugus Reservation southwest of Buffalo in the 1950s. In 2018, the Seneca Nation filed a lawsuit against the state, asserting that the state had built the highway illegally on Tribal land and noting that decades of toll proceeds have gone to the state rather than the Tribe.^18^ The dispute led to delays in repairing the road, leaving it with potholes and other hazards that required the speed limit to be decreased from 65 to 45 miles per hour.^19^

Climate challenges unfold against this backdrop of historical conflicts and inequities. However, there are also opportunities to address transportation inequities by developing climate adaptation strategies that include Indigenous participation. Such inclusion can also lead to the incorporation of Indigenous knowledge of flood control and watershed management; land erosion prevention practices; and adaptation techniques that use ecological resources such as plants, soil, and water.^20^

### 2.6 Opportunities for positive change

Though a changing climate will have certain negative impacts on the state's transportation system, tackling this challenge offers opportunities for positive change. For example, the federal and New York State governments, along with many municipal governments, have aligned in recent years with the resources and intent to not only repair the state's aging transportation system infrastructure, but to redevelop it to be both more resilient and more equitable. Recent legislation and policies have boosted investment in transportation and other forms of infrastructure. With this investment comes an opportunity to explore how existing transportation systems, many designed generations ago, contribute to structural vulnerability and inequity throughout the state.

Climate adaptation strategies in transportation also present opportunities for cobenefits, not only within different transportation modes but also through other sectors that support or interact with transportation. For example, improving roadways to make them less prone to flooding can also reduce stormwater runoff that can overwhelm sewer systems. Expanding biking and walking infrastructure can help reduce energy use, reduce emissions of greenhouse gases and other air pollutants, and contribute to people's health and well‐being.

## 3 ROADS AND HIGHWAYS

### 3.1 Background

#### 3.1.1 Mode description

Driving is the most prevalent mode of transportation in New York State. Figure 9‐1 shows that as of 2022, more than 57% of workers in the state got to their jobs by driving (the sum of “drive alone”; “carpool”; and “taxi, motorcycle, other”).^3^ These numbers demonstrate the importance of roadways to the state's economy and its residents’ livelihoods.

The structural condition of the state's roadway infrastructure is a key factor in assessing climate change impacts, as infrastructure in poor condition can amplify climate hazards. The consequences of this deterioration are well known to motorists: potholes and uneven surfaces that increase vehicle wear and tear, bridge weight and traffic restrictions, and safety hazards that can contribute to accidents. Deteriorated roadways that fail during an extreme climate event can impede mobility, emergency services, and supply chains.

New York State's roadways are owned and managed by a variety of entities, including federal, state, Tribal, county, and municipal governments. Although local government spending on roads outside of New York City has declined over the last decade compared with overall local infrastructure spending,^21^ state and federal agencies have planned for large amounts of roadway infrastructure spending over the next decade. The federal Bipartisan Infrastructure Law, signed in November 2021, provided $110 billion in new federal spending for upgrading roads and bridges, including increases in dedicated funding for state highway and transportation agencies.^22^

#### 3.1.2 Scope

For the purposes of this chapter, roadways include all pathways that are accessible by a wheeled motorized vehicle, from rural dirt roads to paved streets and expressways. This mode includes bridges and tunnels, overpasses, interchanges, and auxiliary systems that protect fixed infrastructure and enable the smooth and safe flow of vehicle traffic. Auxiliary systems include:

- Drainage systems, encompassing culverts, ditches, sewers, and other forms of water diversion to direct the flow of water under and around roadways.
- Electrical infrastructure integrated with roadways, including lighting, signals, railway barriers, and powered signage.
- Energy infrastructure in the form of gas stations and, increasingly, EV charging systems.

#### 3.1.3 Connections

Roadways are present throughout the state, and they support loads of every type, whereas other modes (i.e., mass transit, rail, maritime, air, micromobility) are more limited in geographic scope and in the type of loads they are designed to transport. Roadways complement the other modes discussed in this chapter by providing “first‐mile” and “last‐mile” transportation, such as when a person drives from home to a train station or airport, or when goods that have crossed the country by train are delivered to stores by trucks. Roadways support buses and micromobility and they also act as the redundant mode, or backup, when other modes fail. These characteristics make roadways integral to trips and supply chains that involve other transportation modes.

One consequence of this integration is that climate‐driven impacts to roadways could cascade to other transportation modes and vice versa. In addition, intermodal connection points—places where travelers or goods are transferred from one transportation mode to another—can be especially vulnerable to climate hazards, as discussed in Section 3.2.1.5.

Roadways are also fundamental to the functionality of other sectors discussed in this assessment. The transportation sector accounts for nearly 30% of the state's total energy use.^23^ Most of this energy is consumed as conventional petroleum‐based fuels. While the Energy chapter discusses the provision of fuels and associated infrastructure such as pipelines, this chapter discusses how climate change can affect the delivery of fuels to end users, such as by causing shortages at gas stations or other points of consumption.

### 3.2 Observed and projected impacts

New York State's roadways are at risk of damage from water, extreme heat, freezing/thawing, and extreme wind. Water is the driving force in hazards such as heavy precipitation, flooding, snow and ice, fog, sea level rise, storm surge, erosion, and corrosion. Water‐driven impacts vary greatly across the state based on factors such as local climate, topography, and proximity to the Great Lakes or Atlantic Ocean. Impacts from heat, cold, and wind also vary geographically. Additionally, the impacts of all these climate hazards vary due to socioeconomic factors such as population density, wealth and resource distribution, and economic activities.

#### 3.2.1 Impacts on the physical system

Like all modes of transportation, roads and highways are designed to function within specific expected ranges of travel demand and climate conditions. Climate conditions outside of the expected ranges interfere with the physical functionality (e.g., structural integrity, viability, safety conditions) of roads and highways. Table 9‐1 lists many climate hazards and their potential impacts to New York State's roadway infrastructure and driving conditions. The Fifth National Climate Assessment, released in late 2023, suggests some additional potential impacts.^24^

**TABLE 9‐1:** Impacts of climate change on road infrastructure.

Climate hazard	Possible impacts to road infrastructure
Temperature: high temperatures, heat waves, daily temperature variation	Thermal expansion at bridge joints and other impacts to concrete pavement infrastructure. Reduced pavement life and other pavement integrity concerns, such as relatively large deflections, transverse cracks, and reduced asphalt‐layer stiffness and strength.
Temperature: freezing/thawing	Differential freezing (slippery conditions form on the surface of isolated sections of pavement, while adjacent sections maintain dry surface conditions). Premature deterioration of pavement structures, resulting in increased cracking. Road blockages, interrupted access, and infrastructure damage from avalanches and rockslides. Infrastructure damage due to slush flows. Deterioration of porous asphalt, resulting in raveling, pothole damage, and material loss at longitudinal joints. Plastic deformation (sinking) of roads, bridge foundations (collapse), drainage pipes, and pavement layers. Damage to roadways (shoulder erosion, increased potholes, even sinkholes) caused by rising water tables and flooding due to rapid thawing.
Precipitation: intense precipitation	Decreased pavement life due to premature damage to subgrade materials and structure. Overloading of drainage systems, causing backups and road flooding. Road damage from mass movements of soil and rocks (slides). Increased soil moisture levels, affecting the structural integrity of roads, bridges, and tunnels. Foundation damage to bridges, culverts, piers, and abutments due to abrasion/scouring. Accelerated deterioration and failure of roadways after flooding. Bridge overtopping and damage caused by flooding. Increased risk of wildfires due to increased precipitation, which leads to accelerated growth and death of vegetation.
Precipitation: low precipitation, drought conditions	Ground stability impacts (desiccation, shrinkage of clay materials). Infrastructure instability. Increased susceptibility to wildfires that directly threaten transport infrastructure. Loss of soil cover, allowing for accelerated erosion when rain occurs and for soil fines to reach drainage systems.
Sea level rise: storm surge and coastal inundation	Road flooding in coastal areas, and more frequent or severe flooding of underground tunnels and low‐lying infrastructure. Decreased pavement life when unbonded layers become saturated. Premature road pavement failures. Erosion of the road base and bridge supports. Loss of coastal wetlands and barrier shorelines that protect roadways from coastal flooding and damage. Gradual land subsidence, increasing risks of road fractures and sinkholes. Asphalt detachment and deterioration of cement‐treated bases from higher salinity.
Extreme storms	Dangerous driving conditions. Roadway and tunnel flooding. Road blockages from fallen trees, downed power lines, flash floods, and landslides. Erosion of road platforms or land adjacent to the road. Blockage of drainage systems due to accumulation of debris. Road disruption from wind‐blown debris, such as vegetation. Power and communication outages that cause traffic signal and signage failure and transportation delays. Fuel shortages that hinder the use of roads in emergencies. Inhibited passage of emergency vehicles, inability to follow evacuation protocols, and disrupted delivery of goods and critical supplies. Delays in scheduled maintenance and repairs.
Wildfires near highways	Burning of asphalt. Failure or melting of components such as guardrail posts. Greater susceptibility to landslides in areas deforested by forest fires.

*Note*: Table adapted from Souza de Abreu,^25^ licensed under CC BY 4.0.

The subsections below provide additional details on the ways in which climate hazards affect specific types of physical roadway infrastructure, including surface roadways, bridges, tunnels, culverts and other drainage infrastructure, intermodal connection points, and power and communications infrastructure.

##### 3.2.1.1 Surface roadways

Extreme heat can damage and degrade roadways. Asphalt softens in extreme heat, potentially affecting the shape of the roadway surface and reducing the lifespan of the roadway. Routine repaving with updated asphalt mixes can effectively mitigate this impact, but increasing the frequency of repaving raises costs. Concrete slabs, built with spacers to account for heat expansion, are susceptible to compressive forces and cracking if they run out of room to expand during prolonged periods of extreme heat. Areas with high densities of paved surfaces, especially urban regions, will experience elevated levels of heat risk due to the heat island effect, compounding the effects of hot weather on road infrastructure. Most of New York State's roadway infrastructure was built to withstand temperature ranges that were typical of the 20th century, which puts it at risk of damage as heat waves become more common and more intense.

Washouts can drastically reduce the interconnectivity of surface roadways, while extreme wind from increasingly intense storm systems can cause dangers and disruptions from debris, such as fallen trees or downed power lines, with extreme gusts threatening to overturn vehicles, blow them off the road, or cause collisions. Several storm events in recent years have caused serious disruption to some of the state's surface roadways, including:

- **Hurricane Irene**. In August 2011, rain, heavy winds, and storm surge from Hurricane Irene caused widespread transportation disruptions in New York State. Twenty‐seven bridges across nine different counties were closed due to flooding, washout, and safety concerns. More than 115 highway closures occurred across 21 counties due to flooding, fallen trees and power lines, culvert collapse, debris, and road damage.^26^
- **Tropical Storm Andrea**. By the time Tropical Storm Andrea reached New York in June 2013, it had weakened substantially. Nevertheless, the storm brought 4 inches of rain and heavy winds that caused lasting impacts to roadways and travel in the state. More than 400 members of the New York State Department of Transportation's (NYSDOT's) road maintenance staff prepared in the days leading up to the onset of the storm. A few days after the storm hit New York, rain and fog persisted, and NYSDOT was still advising drivers to avoid low‐lying, flood‐prone, and coastal state roads (including seven main highways), as pumping and drainage operations and emergency lane closures would be in effect.^27^
- **December 2022 snowstorm**. In December 2022, a prolonged winter storm affected a large region of the United States, including Buffalo.^28^ More than 3 feet of snow fell in the area, making roads impassable and stranding hundreds of people in their cars. Critical infrastructure was also disabled, including power and water systems.

According to a report by the American Society of Civil Engineers, deferred maintenance has left 45% of New York's state‐maintained surface roadways in fair or poor condition, which exacerbates the risk of damage from extreme weather.^29^

##### 3.2.1.2 Bridges

New York State has 17,557 bridges.^30^ Bridges are a potential source of traffic bottlenecks, and damage caused by climate hazards can compound this risk to the smooth flow of traffic. The deterioration of aging bridges adds to the risk, as 1611 of the state's bridges, or 9.2%, are rated in poor condition.^30^ Figure 9‐2 shows the concentration of such bridges by county, along with a general indication of flood risks—one known climate stressor.

**FIGURE 9‐2:**
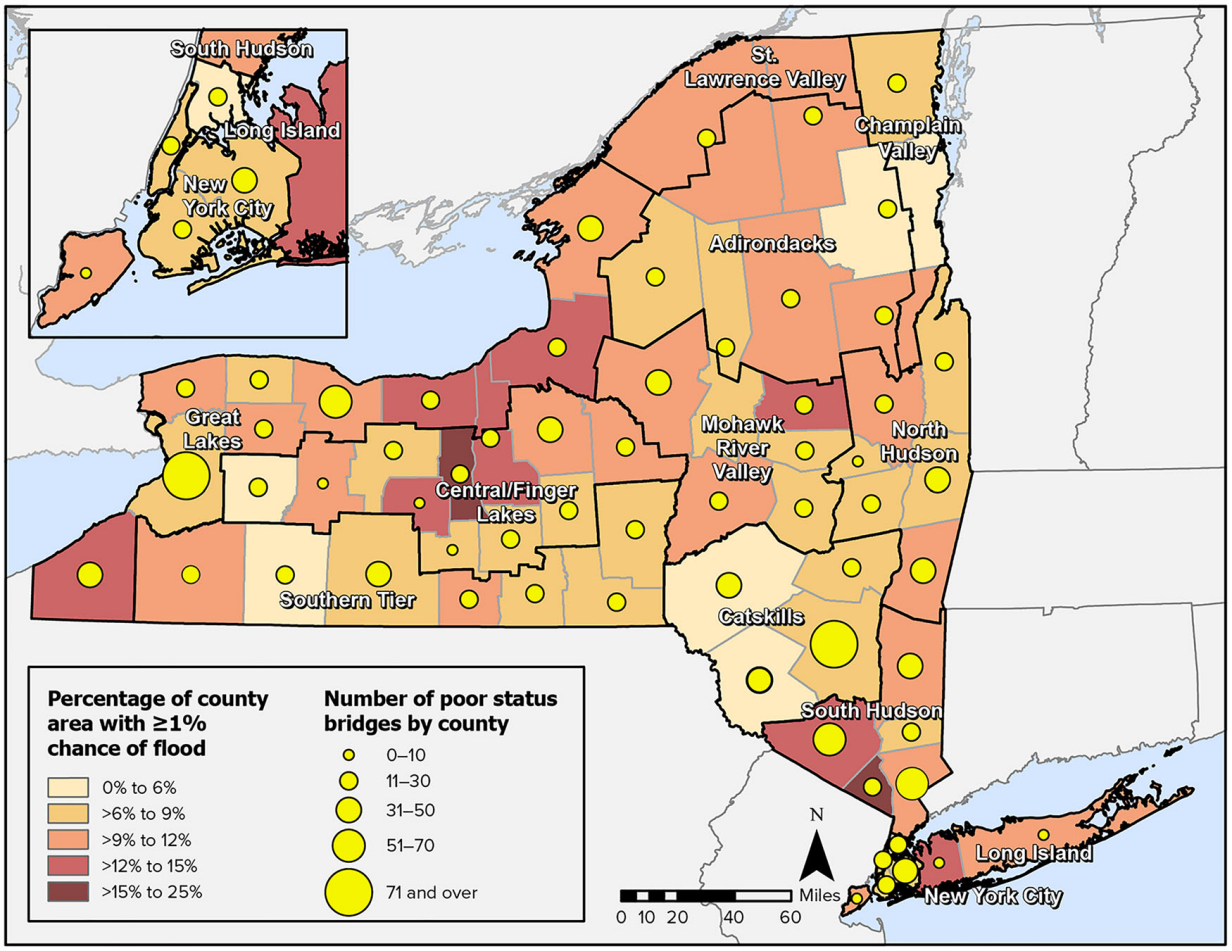
Concentrations of flood risk and poor‐rated bridges by county. Data from U.S. Environmental Protection Agency (2018)^31^ and Federal Highway Administration (2022).^30^

Climate hazards can exacerbate the deterioration of roads and bridges, causing a need for further maintenance and funding. Increases in precipitation and storm intensity are a main threat, as heavy precipitation events can lead to washouts, flooding, and damage. Rapid thawing of ice and snow can also produce riverine flooding by inundating channels and drainage systems with meltwater amounts beyond their flow capacity. As roads are often built near river and stream channels, such flood events often lead to roadway flooding, closures, and damage to infrastructure (i.e., road surfaces, bridges, drainage culverts).

Extreme temperatures also pose a threat. The projections developed for this assessment examined the number of days per year over 95°F—a threshold that by itself is not typically hazardous to bridge components. However, the marked increase projected statewide for days over 95°F, along with other projections that consistently show increases in extreme heat for the rest of the 21st century no matter what definition or threshold is used, suggest that days hot enough to pose a risk to bridges will become more probable than they are now.^5^

Rapid temperature fluctuations that could occur more often with extreme weather patterns also pose a risk to bridges, especially because rapid heating or freezing can be difficult to prepare for and can produce unpredictable impacts. For example, in 2019, a rapid morning thaw dislodged large amounts of ice and water on the Hudson River near Albany, causing several vessels, including a 600‐passenger cruise ship, to break loose.^32^ The unsecured vessels collided with multiple road bridges and a rail bridge linking Amtrak's passenger service, closing the affected bridges for hours. The collisions and subsequent closures occurred over a 6‐mile stretch of the river, stalling traffic and temporarily reducing road connectivity across the Hudson.

##### 3.2.1.3 Tunnels

New York State has tunnels that serve as throughways for roadway, subway, and rail systems. These tunnels can flood during periods of high precipitation and storm surge, which are projected to worsen over the rest of this century.^5^ In October 2012, Superstorm Sandy flooded numerous subway and railroad tubes in New York City, as discussed in Section 4.^33^ Several road tunnels flooded as well, including the Holland Tunnel (connecting New York and New Jersey) and the Brooklyn‐Battery Tunnel (connecting Manhattan and Brooklyn), which filled with millions of gallons of water.^34^

In 2023, the Port Authority of New York and New Jersey (PANYNJ) announced a project to repair the Holland Tunnel, more than 10 years after Superstorm Sandy flooded the tunnel with more than 30 million gallons of brackish water.^35^ The repairs are extensive and will cover mechanical, electrical, communications, and plumbing systems harmed by the salt left over from the flooding. The tunnel is projected to undergo partial closure six nights per week for nearly 3 years.^35^ Traffic will be diverted to other crossings,^36^, ^37^ which will likely increase travel times for many drivers. These nightly closures exemplify the toll of extreme storms on mobility and how the effects of a severe storm can be felt many years later.

##### 3.2.1.4 Culverts and drainage

By channeling water under roads, highways, and other infrastructure, culverts can protect infrastructure from flooding. Culverts are designed to mimic natural stream function, and some can carry floodwaters exceeding the levels of a 100‐year storm.^38^ A right‐sized culvert can greatly reduce damage to nearby infrastructure during flood events. However, New York has experienced a large increase in the number and severity of extreme precipitation events, and that increase is expected to continue.^5^ Culverts typically have an expected service life of 50−100 years,^39^ and many existing culverts were not designed to carry the volume of discharge that is expected in the future under peak flow scenarios. In some cases, building culverts large enough to accommodate the ongoing and expected increases in precipitation frequency and intensity may not be possible due to other environmental limitations in the area.

Through its BRIDGE NY initiative, New York State provides funding to local governments for the rehabilitation and replacement of bridges and culverts. One of the initiative's stated aims is to improve the resilience of structures in the face of climate change. In July 2023, the state announced more than $516 million in awards, including funding for the replacement of roughly 100 culverts statewide.^40^

##### 3.2.1.5 Intermodal connection points

Roadways intersect with all other sectors and modes of transport. Often, substantial vulnerabilities exist where roadways connect to another transportation modes. For example, the point at which various roadways converge to allow trucks to load and unload freight at the Port of New York and New Jersey is vulnerable to sea level rise and storm surge. In general, intermodal connection points at maritime ports are among the most vulnerable locations in New York State's highway network and the freight trucking supply chain.

Many New York City roadway and rail intersections are also vulnerable intermodal connection points. During Hurricane Ida, many roadway drainage systems in New York City failed. Street flooding not only made ground transportation options unusable, but also led to flooding of subway stations.^41^ At intermodal connection points like these, climate disruptions can affect two or more modes at once, nullifying modal redundancies and alternative routes. Even where modes may be individually resilient, the points where they connect can become vulnerable to cascading impacts.

##### 3.2.1.6 Electrical infrastructure

Extreme weather events can disrupt the electrical infrastructure associated with roadways, such as traffic signals, street lighting, and railway barriers. Power outages that disable electrical infrastructure can affect traffic conditions, residents’ safety, and emergency response efforts. For example, in August 2003, one of the largest power outages in U.S. history occurred, leaving residents in New York, other states, and parts of Canada without power for 29 consecutive hours due to a cascade of damaged transmission lines that started with a fire in Ohio.^42^, ^43^ During the outage, all 11,600 signalized intersections in New York City ceased to operate; the New York City subway system came to a halt, stranding 400,000 passengers in tunnels; and the commuter rail network closed, stranding many more people. With public transportation shut down, residents turned to taxi cabs, and thousands of people walked to their destinations. In addition to these major systems failing, auxiliary transportation systems also began to fail, including tunnel lights and ventilation; intelligent transportation system equipment including cameras, loop detectors, variable message signs, and electronic toll collection equipment; and pumps that control flooding in depressed roadways.^42^, ^43^ Telecommunication networks also failed due to a combination of overwhelming wireless call volumes and loss of primary power at transmitter stations^44^—an impact that was notable in 2003 and would likely be even more disruptive in present or future conditions given the increased reliance on telecommunications in transportation (e.g., intelligent transportation systems, connected and autonomous vehicles, mobile apps for ridesharing).

Although mitigation plans and strategies had been developed for responding to transportation network failures as a response to previous blackouts and disasters, New York City was not prepared for a blackout of this size and duration. First‐response efforts focused on assisting people who were in immediate danger, such as those stuck in elevators or trapped in the subway; traffic management was a secondary priority.^42^ In many cases, ordinary citizens stepped up to direct traffic at major intersections where first responders and law enforcement authorities were not able to assist.

#### 3.2.2 Impacts on the movement of people

Extreme weather events affect the movement of people in the following ways:

- **Impeding safe vehicle operation**. If weather conditions create poor visibility or reduce traction, the driving environment immediately becomes less safe. A study on the way snowfall affects traffic volume and crash risk on the western half of the New York State Thruway system determined that anywhere from 35% to 50% of crashes in the month of January are associated with inclement weather.^45^ As a result of inclement weather, 6124 travelers in the state died from traffic‐induced car accidents over the course of 5 years (2015−2020). As the state experiences more days with heavy precipitation in a changing climate,^5^ New Yorkers will spend more time driving in more hazardous road conditions, potentially increasing the number of weather‐related crashes.
- **Creating delays by cutting off travel routes and increasing congestion**. Road closures resulting from severe storm impacts, such as fallen trees, washouts, and increased accidents, lead to increased congestion of alternative routes. In 2016, a study of three alternative travel routes in Buffalo demonstrated the relationship between travel time variability and inclement weather conditions caused by rain and snow. The study found that travel time delays increased by up to 54% during snowy conditions, while rainy conditions caused travel time delays to increase up to 58%, depending on the route taken, day of the week, and time of day.^46^ Moreover, when a severe storm is forecast, people may try to evacuate the area before the event occurs, which can cause congestion along common evacuation routes.
- **Slowing emergency response and repair efforts**. The latter can delay the rebuilding and recovery process.

The impacts of increased roadway traffic include not only longer travel times and lost time for motorists, but also increased exhaust pollution and the financial cost of wasted fuel and vehicle wear. Exhaust pollution from idling vehicles includes greenhouse gases that contribute to climate change, as well as particulate pollution that poses risks to public health.

#### 3.2.3 Impacts on the movement of goods

Throughout the state, trucks transport roughly 472,000 tons of manufactured goods per day, and over 89% of New York's communities depend exclusively on trucks to transport their goods.^47^ Even for goods moved by cargo ship or rail, trucks frequently provide “first‐mile” and “last‐mile” transportation: they carry goods from manufacturing facilities to ports or rail yards, and at the other end of the journey, they move goods from distribution centers to businesses and retailers. Climate hazards that disrupt the movement of goods (the transport supply chain) are largely the same as those that affect the movement of people. However, there are a few differences with respect to infrastructure usage, degrees of impact, and the ways a single hazard can interfere with transporting loads.

Localized climate‐driven impacts to roadways can manifest as supply chain disruptions, delaying the transportation of raw materials and commodities for manufacturing, goods heading to market, and products delivered to consumers. Fuel is also largely transported to distribution points via the roadway system. If transportation fuels cannot reach gas stations or fuel depots due to severe storm impacts, the movement of goods will be further disrupted. Additionally, extreme precipitation, extreme heat, or sea level rise can affect multiple transportation systems involved in the movement of goods. For example, sea level rise can affect not only vessel access to a port but also the roadways and rail systems leading to the port. This creates a compounding effect that further disrupts the state's supply chain.

With the advent of online shopping and the growth in on‐demand delivery options, the demand for freight transportation of goods has increased dramatically. One national transportation research group has projected that the value of goods transported between sites in the state will increase 108% by 2045.^48^ In addition, congestion on New York roads already poses substantial challenges for the transportation of goods. According to an American Transportation Research Institute study that looked at the movement of freight across U.S. highways, 6 of the 100 worst trucking bottlenecks in the country occur on New York roadways.^49^ These congestion problems could grow worse as the volume of freight transportation continues to grow and changing climate conditions disrupt travel, causing further delays in the delivery of goods across the state.^49^

### 3.3 Vulnerable populations and systems

Climate impacts on roads and highways have the potential to affect all New Yorkers, either directly or indirectly. However, due to economic, political, and social factors, some individuals and groups are especially vulnerable to these impacts. The following sections consider the vulnerabilities of populations, regions, and industries to the climate hazards discussed in Section 3.2.

#### 3.3.1 Vulnerable populations

The climate impacts to roadways will have distinct and magnified consequences for the following groups of people:

- **Essential workers**. While many workers can effectively conduct their work remotely, a substantial portion of New York State's workforce must commute each day. A 2023 survey by the Federal Reserve Bank of New York found that 68% of employees at service firms in the state and 94% of employees at manufacturing companies work in‐person only.^50^ The U.S. Centers for Disease Control and Prevention defines essential workers as “those who conduct a range of operations and services in industries that are essential to ensure the continuity of critical functions.”^51^ Health care workers, law enforcement, first responders, construction workers, utility electricians, grocery store clerks, childcare providers, and many other essential workers may struggle to perform their jobs if climate conditions make roads impassable or disrupt transit. People who rely on the services of essential workers, such as hospital patients who need nurses, may also be affected. Workers in the transportation sector, including bus drivers, truck drivers, and subway engineers, are also essential workers. These employees may require functioning road infrastructure not only to travel to their job sites, but also to perform their work.
- **Low‐income individuals, households, and communities**. Low‐wage workers with earnings less than or equal to the 25th percentile are six times less likely to be able to work from home than higher‐wage workers with earnings greater than the 75th percentile.^52^, ^53^ As a result, when roads are affected or degraded by climate change impacts, low‐wage workers may experience lost work time, a reduction in working hours, vehicle repair costs due to poor road conditions, and expenses related to supply chain disruptions.
- **People of color**. Communities with obstructed streets and other roadway infrastructure damage depend on recovery personnel and resources to help clean up the damage in a timely way. National‐level research has shown that, on average, predominantly white communities receive more aid from the Federal Emergency Management Agency than communities of color who have incurred the same severity of damage.^54^
- **Rural residents**. People who live in rural areas often commute long distances for work and rely exclusively on personal vehicles and roads for their daily transportation needs. In the wake of extreme weather events that close or damage roadways, rural residents may have few backup options to get to where they need to go, and they anecdotally have faced longer wait times than others (even multiple days or weeks) before receiving storm relief on local roads. Additionally, rural residents typically have less access to emergency medical care than urban and suburban residents do, with ambulances taking longer to arrive and hospitals tending to be further away. More than 300,000 residents of New York State live outside a 30‐min drive from the nearest hospital.^55^ When climate events damage roads or disrupt travel, the risks that rural residents face from having limited access to medical care are compounded.
- **Small business owners**. Small businesses often depend on in‐person services and sales; consumers must be able to travel to the business. In rural areas and small towns without mass transit options, when climate events affect travel or the delivery of goods by roadways, small business owners are at risk of lost income or business failure. Local markets or stores that are not part of larger, multisite companies have a concentrated climate risk at a single site and will be particularly vulnerable to consumer or supply chain disruptions.
  Some owners of small businesses are independently employed contractors or gig economy workers who directly rely on functioning transportation for their livelihoods. The New School estimated that New York State had more than 151,000 app‐based workers in 2018,^56^ most of whom provide a transportation service such as ride‐hailing or deliveries. Where roadways are impacted by climate hazards, these workers may experience short‐term losses of income if they are unable to reach customers or miss delivery windows due to traffic congestion and detours. However, climate events could also increase demand and income opportunities for driving services, incentivizing app‐based workers to drive in hazardous conditions, which would increase risks to themselves and their passengers.
  Trucking companies represent an additional segment of small businesses dependent on roadways. Many truckers are independently employed contractors or work for other types of small, locally owned businesses^47^ and have little margin to absorb the cost impacts of climate‐disrupted roadways (e.g., delayed deliveries due to route blockages or detours, increased labor hours, and increased fuel costs). As roadway hazards increase from a changing climate, so could the costs of insurance, further narrowing truckers’ business margins.
- **Mobility‐impaired individuals**. Individuals with personal mobility impairments may have difficulty accessing certain transportation options, such as mass transit. As a result, they may depend on personal vehicles, taxis or rideshare services, or assisted vehicular services for daily transportation needs.^57^ Impassable roadways and obstructed routes may prevent them from getting help or accessing essential services in a timely way.
- **Older adults and people with chronic health conditions**. Some older adults have limited mobility and may face some of the same challenges as mobility‐impaired individuals. People with chronic health conditions—including many older adults—often depend on a regular supply of medication and access to health care providers that could be affected by extreme weather and disrupted road conditions. Impassable roads and bridges can also hinder first responders from getting to people suffering from a sudden health emergency.

#### 3.3.2 Vulnerable sectors and industries

Climate‐related disruptions to roadways have a disproportionate impact on economic sectors and industries that rely on an on‐site workforce to provide services or perform physical labor. Sectors and industries in New York that rely on an on‐site workforce include health care, child care, agriculture, construction, utilities, transportation, manufacturing, and hospitality. These sectors depend on functioning roadways to ensure the movement of the workforce, especially in nonmetropolitan regions where workers rely on vehicles and have little or no access to mass transit. Some of these sectors also depend on roads to move goods to market—including agriculture, where transportation is especially time‐sensitive with foods at risk of spoilage.

Trucking is inherently sensitive to roadway disruptions. In the immediate aftermath of a climate‐related event, rerouting trucks can contribute to supply chain delays. The industry could also face longer‐term disruptions, such as increased road construction to address climate‐driven roadway degradation or severe storm−related flooding. Because trucking, whether long‐distance freight or local distribution, is the primary means of supply chain movement in the state,^47^ climate‐driven roadway problems that directly impact this industry could cascade and affect many other sectors, potentially leading retailers and consumers to experience shortages.

The tourism industry is important to New York State. Tourists can use multiple modes of transportation to get to and around New York City, but in other regions of the state, roadways are the primary way people get to destinations such as agricultural tourism farms, vineyards, historical sites, small towns, state parks, resorts, and lakes. Tribal tourism also depends on tourists arriving by roads to visit heritage sites, museums, and gaming centers. Many rural communities’ economies are based primarily on tourism and recreation and could experience considerable cascading effects from climate‐impacted roadways.

BOX 2 **Climate vulnerabilities of electric vehicles**

The transportation sector is currently the United States’ largest contributor to greenhouse gas emissions, with the majority of the sector's emissions coming from motor vehicles on the nation's roads and highways.^58^ Federal and state policies seeking to reduce greenhouse gas emissions from vehicles are guiding the transportation sector toward low‐ and zero‐emission vehicle adoption. New York State adopted California's Advanced Clean Cars II Regulations, a set of standards that aims to scale down and eliminate sales of new gasoline passenger cars by 2035,^59^ putting pressure on automakers to accelerate EV research and production.^60^

Yet, even as EVs are viewed as a major piece of the solution, they present some vulnerabilities to climate hazards, especially extreme conditions that threaten the functioning of the electric power grid. For example, severe storms and other climate hazards can cause power outages that may leave EV motorists unable to charge their vehicles. Severe weather and flooding can also damage the EV charging stations themselves.

Testing shows that extreme temperatures, both hot and cold, reduce the usability and driving range of EVs, with various estimates placing the optimal temperature range in the neighborhood of 70−85°F.^61^, ^62^, ^63^, ^64^ These results reflect a combination of temperature impacts on the battery itself and the fact that in an EV, heating or cooling the passenger cabin requires energy from the battery. With extreme heat projected to increase during this century and extreme cold projected to decrease,^5^ EV drivers could experience improved efficiency and range during colder parts of the year but decreases on the hottest days.

#### 3.3.3 Vulnerable regions

Certain regions of the state are particularly vulnerable to climate change impacts on roadways. The coastal and tidal regions of Long Island, New York City, and the Hudson River Valley are vulnerable to sea level rise and associated hazards, including coastal flooding and storm surge. Erosion tied to sea level rise can threaten the integrity of coastal roads and streets, while salt water can corrode metal at the base of bridges, shortening their usable lifespan. These areas will require substantial resources both for emergency response and for the design and implementation of adaptive measures that improve infrastructure resilience.

Some regions will experience heightened vulnerability to intense storm impacts. Streets in urbanized areas, for example, could be especially prone to drainage problems from intense flooding, as demonstrated by the Sauquoit Creek Infrastructure Adaptation and Hurricane Ida Vulnerabilities in New York City case studies. Roadway flooding will become an increasing concern in rural areas of the state where a lack of alternative routes could block access for residents and businesses.

Projections suggest that the composition and severity of winter precipitation events will change under future climate conditions. While snowfall, snow depth and extent, and snow water equivalent (the amount of water that falls and accumulates in the form of snow) are projected to decrease across most of New York State over the next century, the frequency and intensity of lake‐effect precipitation may increase under warming lake conditions.^5^ This is projected to lead to an increase in lake‐effect snow over the next few decades, but as the state continues to warm, the additional precipitation caused by the lake effect will increasingly fall as rain rather than snow.^5^ The snow season is also projected to become compressed.^5^ This increase in lake‐effect snow over the next few decades in regions directly downwind of the Great Lakes could result in increased burdens on transportation departments, as demonstrated in the Lake Effect Snowstorms case study.

### 3.4 Adaptation and resilience

#### 3.4.1 Known methods

Weather is estimated to account for 21% of the approximately 5.8 million crashes per year in the United States.^65^ Conditions that contribute to these crashes include wet pavement, rain, snow/sleet, icy pavement, slushy pavement, and fog.^65^ Typical adaptation responses to weather‐related road hazards include road treatment strategies, speed limit control, access control, construction planning, and evacuation.^65^

More broadly, the following adaptation and resilience strategies can help prevent or minimize climate change impacts on roadways:

- **Physical structure improvements**. Improving the physical structure of roadways and their associated systems (e.g., drainage systems) can prevent damage and slow the deterioration caused over time by climate hazards. For example:
  ∘ Many existing culverts were not designed to carry the volume of discharge that is currently occurring or is expected in the future under peak flow scenarios. Replacing existing infrastructure with right‐sized culverts can help reduce flooding that damages roadways and disrupts travel. NYSDOT recently revised its design criteria for culverts to align with requirements in the *New York State Flood Risk Management Guidance for Implementation of the Community Risk and Resiliency Act*.^66^, ^67^ As described in Section 3.2.1.4, the BRIDGE NY initiative is providing funding to local governments for the replacement and rehabilitation of outdated culverts statewide.^40^
  ∘ Greater awareness of the need for climate resilience has prompted the development of new design standards for resilient roadway infrastructure that reflects updated climate projections in addition to historical trends.^67^ Design codes are also driving the use of new technologies and materials, such as permeable paving materials to reduce storm runoff.^29^
- **Dedicated funding**. In response to the widespread damage caused by Superstorm Sandy in 2012, the state established the Extreme Winter Recovery program. Under the program, municipalities can receive state funds from the Consolidated Local Street and Highway Programs (CHIPS) to maintain and repair roads and bridges subject to undue stress from severe weather. In the 2021 fiscal year, the Extreme Winter Recovery program was funded at $100 million, which was $45 million more than the previous fiscal year.^29^ Because the needs for roadway and bridge maintenance and repair are expected to increase with more frequent and severe climate‐driven weather events, it is helpful for governments to proactively fund these efforts.
- **Project delivery streamlining**. Streamlining project delivery systems during climate‐related emergencies can help transportation departments repair and clean up roadways more swiftly. For example, some procurement laws are suspended during declared federal and state emergencies, allowing transportation departments to use project delivery methods not usually at their disposal, such as sole source procurements and design‐build contracts.
- **Enhanced emergency response**. Projected increases in extreme weather and other climate hazards underscore the importance of having emergency response plans in place at vulnerable locations, strengthening the role of transportation departments in evacuation planning and emergency response, and ensuring that transportation officials work more closely with weather analysts and emergency planners.^68^ Having processes in place to respond to climate impacts in real time is also critical. In 2020, NYSDOT published a Transportation Systems Management and Operations Strategic Plan to address state‐specific objectives for effectively operating the transportation network while providing travelers with safe and reliable travel conditions.^69^ The NYSDOT Transportation Systems Management and Operations Strategic Plan differs from traditional transportation management systems in that it addresses immediate and near‐term needs in system operations rather than long‐term needs. This shift in focus helps NYSDOT better address needs like responding to winter storms across multiple regions, procuring intelligent transportation system infrastructure to better collect and transmit real‐time information, and adapting or incorporating new technologies sustainably and responsibly.
- **Asset management**. In recent decades, many state and federal agencies have established transportation asset management (TAM) practices (Figure 9‐3). TAM can help agencies collect and manage asset data needed to identify vulnerable assets (e.g., culverts, bridges) and make informed decisions about prioritizing projects and allocating resources related to climate change impacts. TAM is “a strategic and systematic process of operating, maintaining, upgrading, and expanding physical assets effectively throughout their life cycle. It focuses on business and engineering practices for resource allocation and use, with the objective of better decision‐making based upon quality information and well‐defined objectives.”^70^ Along with electronic monitoring and smart technologies, TAM practices can also help agencies monitor asset usage, project implementation, and finances.

**FIGURE 9‐3:**
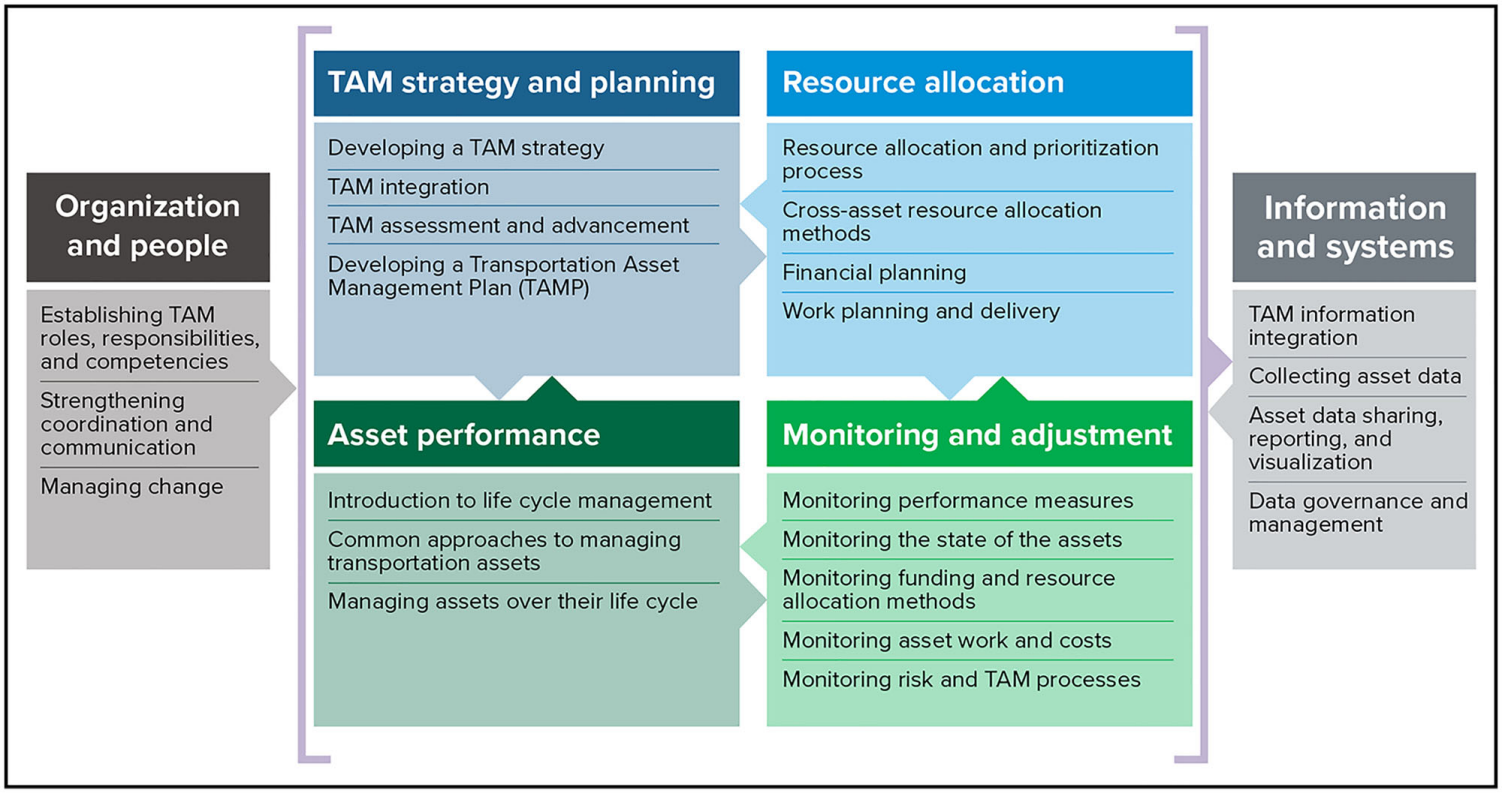
TAM guide framework. Adapted from Figure 9‐2 of the American Association of State Highway and Transportation Officials TAM Guide.^72^ Used with permission.

NYSDOT has developed a Transportation Asset Management Plan (TAMP) to: (1) define NYSDOT's asset management objectives; (2) summarize the inventory and condition of highways and bridges and summarize travel trends; (3) document realistic funding estimates over the next 10 years; (4) document NYSDOT's asset management business structure; (5) prioritize and illustrate risk management and mitigation strategies; (6) describe pavement and bridge lifetime management strategies; (7) define investment strategies; and (8) lay out an agenda for future improvements to asset management and the TAMP.^71^ The NYSDOT TAMP focuses on preserving the functionality and safety of existing highways, viewing individual projects in the broader context of the larger transportation system, maximizing return on investment, and approaching solutions from a sustainability perspective.

#### 3.4.2 Municipal and regional government concerns

Local governments own and maintain more than 96,000 miles of roadways in New York, which is 85% of the state's total.^21^ Yet, many local transportation agencies are small and might not have the financial resources or in‐house expertise to plan for and build resilience to climate impacts on roadways. Practitioners who provided input to this assessment noted that resilience and adaptation literature is vast and often technically complex, and small local agencies might not have the resources to hire staff or consultants with expertise in this area. This limits the development of institutional knowledge and may keep agencies in a pattern of responding reactively, rather than preparing for potential future problems. Anecdotally, under‐resourced local agencies are also less likely to have asset management programs like those described at the state level in Section 3.4.1. Moreover, municipal and regional transportation agencies may find it difficult to justify diverting resources away from their existing maintenance backlog and the immediate need to provide core services like repairing damaged roads, clearing culverts, and keeping roads plowed in the winter.

BOX 3 **$2.7 million roadway resilience project in Cayuga County**

In New York State, increasing cultural and political recognition of climate change impacts on transportation has led to a greater collective response. With communities demanding action, many cities and towns have developed climate action plans with transportation adaptation measures, and several municipalities and regions have adopted climate resilience infrastructure initiatives that involve roadways. For example, the town of Sterling in Cayuga County completed a $2.7 million road construction project in 2021 with funding from the state's Lake Ontario Resiliency and Economic Development Initiative, which is responding to flooding issues on the lake's shores.^73^, ^74^ The project involved installing 1.8 miles of stormwater collection system and building bioretention basins to increase the resilience of town roads to flooding from extreme rainstorms. Before the project, drainage relied on roadside ditches that could not efficiently handle large amounts of runoff and that posed a safety hazard for travelers, emergency vehicles, and local wildlife.

#### 3.4.3 Opportunities and cobenefits

Developing climate‐resilient roadways provides opportunities to address existing inequities around this mode, such as initiatives that improve mobility for underserved or at‐risk communities. For example, the New York State Route 33 (Kensington Expressway) Project in Buffalo involves covering approximately 4100 feet of the below‐grade Kensington Expressway and establishing continuous greenspace and parkland for community residents.^75^ The project not only builds climate resilience by incorporating green infrastructure (vegetative spaces that help manage stormwater and provide other environmental benefits), but it also seeks to reconnect and restore lost equity for the predominantly Black communities on Buffalo's East Side whose property values, local economy, and well‐being had been negatively affected by the construction of the expressway through their neighborhoods.

Investing in New York State roadways to make them more resilient can also bring opportunities for economic growth. For example, public infrastructure legislation passed by federal, state, and municipal governments (e.g., the U.S. Inflation Reduction Act of 2022, the New York State Accelerated Renewable Energy Growth and Community Benefit Act of 2019) promises to create jobs and training opportunities in roadway construction, watershed management, and related resilience work. These efforts could bring jobs to underserved communities where roadways are most vulnerable to climate impacts.

Adaptation strategies for roadways can also offer opportunities for planners to consider ways to reduce greenhouse gas emissions. Planners can explore how roadway resilience and greenhouse gas mitigation techniques can be mutually supportive, such as with new technologies (e.g., inductive roadway charging for zero‐emission EVs) or policies to disincentivize driving and enhance other modes (e.g., mass transit, passenger and freight rail, micromobility). Similarly, because most fuel is distributed via roadways, and electric power delivery infrastructure is often along roadways, adaptation strategies that increase the resilience of roadways can have the cobenefit of contributing to energy security by helping to prevent fuel shortages and power disruptions.

## 4 MASS TRANSIT

### 4.1 Background

#### 4.1.1 Mode description

New York State leads the United States in the use of mass transit, or public transportation. The state's mass transit systems consist of more than 16,000 vehicles that provide a transit service, more than 1700 miles of subway and rail track, more than 150 miles of tunnels, and associated stations and landings.^29^ In 2022, 21.5% of all commuters in the state traveled on public transport, compared with the national average of 3.1%.^3^

Located in the New York City Metropolitan Area, the MTA is the country's largest public transportation agency, generating nearly 40% of all public transportation trips in the nation.^76^ The MTA's operating agencies include New York City Transit, which runs subway and bus service in New York City; the Metro‐North Railroad, which serves counties immediately north of New York City from Grand Central Terminal to Poughkeepsie and Wassaic (and to New Haven, Connecticut); and the Long Island Rail Road, which connects the full length of Long Island to Penn Station in Manhattan.

In all, more than 130 public transit operators serve New York State.^77^ In addition to the MTA, these public transit operators include the Capital District Transportation Authority, serving the Albany area; the Central New York Regional Transportation Authority, serving the Syracuse area; the Rochester‐Genesee Regional Transportation Authority; the Niagara Frontier Transportation Authority, serving the greater Buffalo area; and many county‐based transit systems, as well as private bus operators.^78^ Mass transit systems generally rely on funding from some combination of passenger fares, dedicated tax revenue streams and grants, and public or private capital investments.

The extensive use of public transportation is beneficial for the environment and for other modes of transport as well. It alleviates road congestion, supports more compact settlement patterns, and reduces carbon footprints. New York State, consequently, has the second‐lowest per capita transportation emissions of all 50 states: 4.3 metric tons of carbon dioxide per person in 2018, 27% below the national average.^79^

#### 4.1.2 Scope

Though mass transit systems carry passengers over both land and water, this discussion is limited to land‐based systems. Ferries and water taxis are addressed in Section 6. Land‐based mass transit systems can be further categorized as road‐based or rail‐based.

Road‐based mass transit includes bus transportation and human services transportation:

- **Bus transportation** includes public buses and school buses, operating mainly on fixed routes, though new microtransit services are piloting on‐demand services. Bus transportation relies on road and highway infrastructure for travel and on sidewalks and other multimodal facilities and services to connect with passengers. Bus services are the most widespread form of public transportation in New York State, operating in urban, suburban, and rural contexts.
- **Human services transportation** includes services specifically designed to meet the mobility needs of older adults, people with disabilities, and individuals with low income.^80^

Rail‐based mass transit includes light rail, subways, and commuter rail services:

- **Light rail systems**, including trolleys, typically operate in urban and large metropolitan areas. They carry passengers on vehicles, such as trains, that typically run on electricity and operate at grade (i.e., at street level). Buffalo's Metro Rail is an example of a light rail system.
- **Subways** are heavy rail services. Subways carry a large number of passengers, often with short headways (i.e., the time between adjacent trains). Subways run on electricity using dedicated rights‐of‐way, which are most often grade‐separated, either on overhead trestles or in underground tunnels. The New York City subway carries far more passengers than any other subway in the nation.^81^
- **Commuter rail services** typically extend farther from urban centers than subways, offering scheduled service to surrounding suburban areas. The nation's three busiest commuter railroads serve the New York City area: the Long Island Rail Road, Metro‐North Railroad, and NJ Transit.^81^ Commuter railroads operate on both electric and diesel power.

Long‐distance passenger rail and freight rail are examined separately in Section 5. Mass transit infrastructure includes stations, bus depots, rail yards and barns, tunnels and overpasses, power substations, pumps, ventilation plants, control centers, and signal towers.^29^ Another fundamental component of mass transit is communications systems, which have become increasingly important as shared transportation platforms emerge and grow, along with application programming interfaces that provide access to real‐time data.

#### 4.1.3 Connections

Mass transit overlaps with all other transportation modes presented in this chapter. Many trips that people take are multimodal. For example, a person can walk, bike, drive a personal vehicle, or take a taxi to a mass transit hub, or they can take a bus or subway home from an airport. These types of multimodal journeys are particularly common in an urban context given the population density and diversity of modes available. Intermodal connection points are hubs where different forms of mass transit meet one other (e.g., bus‐rail interchange stations) or connect to other modes of travel such as long‐distance rail (e.g., subway connections to Amtrak trains) or air (e.g., rail lines serving airports). Climate‐driven impacts on these hubs can have cascading effects, as described in Section 4.2.

Mass transit also interfaces with other sectors explored in this assessment. For example, mass transit connects people with jobs, educational opportunities, and critical services such as health care, making it vital to the economy and human well‐being. Likewise, there are inherent connections between the energy sector and mass transit, which consumes electrical power and fossil fuels. Mass transit interfaces with the buildings sector through infrastructure and urban planning considerations, as well as through the many buildings within mass transit networks, such as stations and depots. Climate change impacts on building structures and materials themselves are covered in greater detail in the Buildings chapter.

### 4.2 Observed and projected impacts

Key hazards to New York State's mass transit systems include extreme temperatures; heavy rain; sea level rise and coastal flooding; lake‐effect snowfall; and extreme winds, rain, and storm surge from tropical storms and nor'easters.^82^

#### 4.2.1 Impacts on the physical system

The following subsections provide an overview of how specific climate hazards are expected to affect mass transit. The discussion includes impacts to subways, which have vulnerabilities by virtue of being below ground (and sometimes below sea level), as well as aboveground bus, light rail, and commuter rail operations that are more directly exposed to weather conditions.

##### 4.2.1.1 Severe storms and extreme precipitation

Extreme precipitation events are projected to increase in frequency and intensity in New York State.^5^ Projections suggest that days with more than 2 inches of precipitation will become more frequent across the state by the 2050s, and days with more than 4 inches (which are presently rare) will occur at least once per decade in most parts of the state by the 2080s, and five times per decade at locations on Long Island and in the Catskills.^5^ Such heavy storms can affect all forms of mass transit. For example, extreme rainfall can cause flash flooding that overwhelms street‐level storm drainage systems. When storm drains become overwhelmed, water can seep into subway entrances.^41^ Mass transit stations and tunnels often become the default stormwater egress systems. This is not their intended use, and each episode can cause substantial damage.

Heavy downpours can also cause power outages that damage electric substations and equipment,^83^ which in turn can shut down subway and rail service. Buses can become completely inoperable due to flooding. For example, during Hurricane Ida in 2021, Casey Stengel Depot in Queens and Castleton Depot on Staten Island experienced severe flooding.^84^ The flooding irreparably damaged 28 buses parked at the Castleton Depot, causing an estimated $8 million in damage and affecting service.^85^ Flooding can also damage signal systems, line equipment, subway structures, passenger stations, maintenance facilities, and storage yards through structural damage and saltwater corrosion.^84^ Pumping can help remove excess water and prevent flooding in underground rail and subway networks.^82^ However, if pumps become overloaded, the resulting backflow from outlets can have cascading impacts on fan plants and air circulation, potentially increasing fire hazards.^84^

Flooding from extreme precipitation can undermine track support, rail beds, and embankments.^84^ Extreme winds can blow debris onto tracks, increasing the risk of collisions, track damage, and potential disruptions when rail services must pause their operations for debris cleanup.^82^, ^84^ For example, after Tropical Storm Elsa in 2021, service on the Oyster Bay Branch of the Long Island Rail Road was suspended due to downed trees.^86^ (Refer to Section 5 for more detail on impacts to railways.) Over the next few decades, mass transit systems in Northern and Western New York State are projected to face increasing lake‐effect precipitation, including snowfall, due to warmer water and decreased ice cover on the Great Lakes.^5^ Heavy snow could disrupt both bus and rail services. For instance, in November 2022, a lake‐effect snowstorm caused the Niagara Frontier Transportation Authority to suspend bus service in Erie County for a few days.^87^

##### 4.2.1.2 Sea level rise

As described in Section 2.2, sea level is projected to rise along New York State's coastline by up to 1 foot by the 2030s and by about 2−3 feet by the 2080s.^5^ In addition, climate models project that the intensity of tropical storms will increase.^5^ Subway stations within flooding zones and low‐lying coastal areas are at greatest risk from sea level rise. Sea level rise and storm surge from tropical storms can cause flooding and saltwater corrosion of mass transit equipment.^84^ Saltwater cleanup presents a challenge because salt deposits remain on equipment after the sea water is removed, and these deposits cannot always be cleaned on site.^88^ In these cases, the equipment has to be taken elsewhere for cleaning or replacement. Salt is both corrosive and conductive, meaning that improperly cleaned equipment can lead to short circuits and fires. Superstorm Sandy flooded several subway stations and tunnels with salt water and caused a power outage in lower Manhattan, forcing New York City Transit to suspend all subway service between Manhattan and Brooklyn for 5 days.^89^ Following the storm, subway service recovered more slowly than aboveground vehicle service.^89^

Above ground, sea level rise and coastal storm surge can undermine track support, rail beds, and embankments. In some cases, sea level rise may permanently inundate portions of rail. The Metro‐North Railroad's Hudson commuter rail line, which carried 16.9 million passengers in 2017,^90^ has low‐lying sections of track that were submerged during Superstorm Sandy and could be threatened by future sea level rise.^91^ The Long Island Rail Road's Montauk line is similarly susceptible to both storm surge and sea level rise because it runs along the south shoreline of Long Island. Sea level rise can also contribute to groundwater ingress flooding of underground subway systems. The MTA pumps 14 million gallons of water out of subway tunnels on dry days and has the capacity to handle 1.75 inches of rainfall per hour.^92^ Groundwater ingress driven by sea level rise can add stress on these pumping systems.

##### 4.2.1.3 Extreme temperatures

Extreme heat can cause overheating of mass transit equipment and other impacts to vehicles and people. Extreme heat necessitates greater use of cooling and ventilation equipment in response to high underground station temperatures.^82^ More air conditioning on passenger rail cars and buses may also be required for safety and comfort. Most transit buses produced since the late 1990s are equipped with air conditioning systems, and their usage increases with outdoor temperature and heat index.^93^ The use of air conditioning systems on transit buses has also been demonstrated to increase fuel consumption.^94^

Extreme heat can cause road buckling and potholes, both of which put additional stress on buses.^84^ Rails can also buckle during high‐temperature events as the metal expands—creating conditions known as “sun kinks.”^95^ Power outages from stress on the electric power grid during excessive heat events also present a risk for mass transit.

Cold snaps can bring snow and ice accumulation and damage some rail components if they are not tolerant of cold temperatures.^82^ However, extremely cold days are projected to become less frequent in the future.^5^

#### 4.2.2 Impacts on the movement of people

Because mass transit is designed to move people, climate‐driven hazards directly impact the mobility of New York State residents and visitors. In densely populated areas, such as New York City, disruptions to mass transit can have sweeping impacts, inhibiting not only daily commuting but also personal trips for education, business, consumer activity, health care, and leisure. When a climate hazard such as flooding reduces access to mass transit or causes a suspension of service, people often must turn to more expensive modes of transportation (e.g., taxis or rideshare) or use micromobility options (e.g., bikes or scooters) that are more exposed to the elements and may not be safe in a climate‐disrupted situation.

As described in Section 4.2.1, mass transit service outages and delays can occur when climate hazards damage or block throughways such as subway tunnels, tracks, and roadways. For example, when temperatures are high enough to cause rail buckling on commuter rail lines, the standard safety response is to reduce speeds (thereby causing delays) or reduce overall traffic along the lines at risk.^95^ Climate hazards can also damage trains and buses or cause mechanical problems and power outages, resulting in delays in service.

Even when transit systems are running, extreme climate conditions can impact passengers by causing issues with station access and usability and vehicle climate control. For example, it can be a health hazard for some people to wait at an unshaded bus stop during a heat wave or at an unprotected, unheated bus stop during extremely cold winter temperatures or storms. Climate control of mass transit trains and buses is another concern. If extreme external climate conditions are not adequately controlled through air conditioning, heating, and ventilation, trains and buses may become too uncomfortable (and even dangerous) for some passengers.

During heavy precipitation events, subway stations and bus stops on city streets may become dangerous flood zones. Mass transit stations often have stairwells that become dangerous with rain and snow. Those with limited personal mobility or carrying large or wheeled loads (e.g., strollers, suitcases) often access stations through escalators and elevators, which are susceptible to service outages from precipitation or power loss. The loss of lifting devices at stations can be dangerous if these passengers must use the stairs.

Extreme storms can also threaten transportation hubs that serve as intermodal connection points, such as bus‐rail interchange stations. Given the systemic nature of climate impacts and the interconnectedness of transportation modes, disruptions to intermodal hubs can trigger cascading impacts. Residents who are unable to get to work by their usual mode of transportation may seek other modes of travel. Changing travel patterns can cause congestion and additional delays due to the surge of demand in other modes.

#### 4.2.3 Impacts on the movement of goods

Given that mass transit primarily serves to move people, climate‐related mass transit disruptions result in relatively limited impacts to the movement of goods or to supply chains. However, delivery workers do sometimes transport goods via mass transit for efficiency and to save travel time. For example, Amazon delivery personnel have been observed bringing pushcarts full of packages onto the New York City subway.^96^ Climate‐related disruptions to these deliveries would have the greatest impact on those who have personal mobility constraints and cannot easily leave their homes to procure goods at stores.

Integration of freight transport with mass transit may become more prevalent in the future, especially considering that the practice holds promise for improving transportation efficiency and reducing carbon emissions by as much as 50%.^97^ If more integration occurs, the impact of climate‐related mass transit disruptions on the movement of goods may become more pronounced.

### 4.3 Vulnerable populations and systems

#### 4.3.1 Vulnerable populations

While climate‐related disruptions to mass transit have the potential to affect all New Yorkers, not all populations will experience the same impacts. Due to factors such as race and socioeconomic status, the following populations are especially vulnerable:

- **People of color**. A recent study examined 2019 public transportation ridership demographics in New York City and found that 44% of Black residents, 39% of Asian residents, and 36% of Latino residents rely on mass transit for commuting, compared with only 24% of white people.^98^ Climate‐driven disruptions can lead to loss of income for people who rely on mass transit, especially individuals who lack backup transportation options.
- **Low‐income workers**. The COVID‐19 pandemic provided evidence that low‐income workers are especially dependent on mass transit. At the height of the pandemic, when subway usage in New York City was experiencing a sharp drop, ridership remained higher in low‐income neighborhoods than in wealthy ones.^99^ As described in Section 2.4, low‐income workers tend to lack remote work options and are more likely to need to physically commute to work. During climate‐driven disruptions to public transportation, they may be unable to get to their workplace. This situation could cause a loss of income and negatively affect their employment in the long term.^100^
- **People with disabilities and older adults**. Many older adults and people with disabilities rely on human services transportation^57^ and cannot use other forms of mass transit in their communities. They may experience reduced transportation options if the services are disrupted by climate change impacts. In New York City, the challenges for people with disabilities and older adults are compounded by inaccessible subways. The MTA committed to adding elevators and ramps to 95% of the city's subway stations over a 30‐year rollout period, but only 126 of 472 stations (27%) currently have the infrastructure needed to be fully accessible according to the standards of the Americans with Disabilities Act.^101^
- **Rural communities**. Due to their lower population density, rural areas generally have fewer public transportation options than urban areas do. Because of the lack of backup transportation options, any climate‐related disruption to existing services can have a major impact. Low‐income rural New Yorkers who cannot afford vehicles are at particular risk.^102^
- **Indigenous Peoples**. Multiple Native American reservations in New York State have bus transit services, which residents rely upon to access jobs, health care, and education in and around their communities. For example, the Seneca Nation runs the Seneca Transit System, a bus service that provides public transportation between the Cattaraugus and Allegany territories for Nation members, residents, visitors, and surrounding communities.^103^ The Saint Regis Mohawk Tribe offers daily bus services for its elders.^104^ Tribal Nations often lack adequate and safe roadway infrastructure and funding for infrastructure maintenance, which makes their transit systems vulnerable to climate change impacts.^14^

#### 4.3.2 Vulnerable New York sectors and industries

While mass transit is essential to the overall functioning of the economy, some businesses may be especially vulnerable to impacts from climate‐related mass transit disruptions. Industries and sectors that depend on in‐person work may be the most vulnerable, especially when individual businesses are located in areas that rely heavily on mass transit. Industries and sectors that require workers to be physically present on site include goods distribution, construction, manufacturing, agricultural production, health care and pharmacies, food‐related industries (e.g., restaurants, grocery stores), transit, postal and courier services, waste management, social services, and public safety.

Climate‐related mass transit disruptions can have both short‐ and long‐term impacts on affected industries. Punctuated disruptions (e.g., individual storms) will have immediate impacts such as commuter delays and the cascading effects that follow. Over longer time frames, climate‐related hazards can deteriorate mass transit systems, affecting their reliability and efficiency. Regional mass transit is critical to the economy of several parts of New York State, particularly the New York City Metropolitan Area.

#### 4.3.3 Vulnerable regions

Mass transit systems throughout New York State face risks from the climate impacts described in Section 4.2. In some parts of the state, however, subway, bus, and commuter rail systems face special hazards related to regional weather patterns or geography. For example, in the areas immediately east of Lake Erie and Lake Ontario, annual snowfall totals often reach or exceed 150 inches due to lake‐effect storms.^5^ With models suggesting that lake‐effect snow could increase over the next few decades,^5^ heavy lake‐effect snowstorms could pose a growing threat to transit systems like the Niagara Frontier Transportation Authority. However, this threat is projected to decline later in this century, as more precipitation falls as rain rather than snow.^5^ On the other side of the state, the New York City Metropolitan Area faces multiple climate change hazards unique to coastal areas. Sea level rise and coastal storm surge can cause mass transit infrastructure to flood with salt water and corrode.^84^ Permanent inundation of low‐lying mass transit infrastructure is another major risk of sea level rise. These hazards threaten systems (like the Long Island Rail Road and New York City's subways) that are responsible for the daily transportation of millions of people, creating a heightened level of vulnerability.

### 4.4 Adaptation and resilience

#### 4.4.1 Known methods

Adaptation and resilience methods for mass transit systems include a range of rapid and long‐term measures that serve to reduce short‐ and long‐term climate risks. In the New York City Metropolitan Area, sea level rise and storm surge are frequently cited as key risks to mass transit. In 2013, in the aftermath of Superstorm Sandy, the Governor's Office published the NYS 2100 Commission Report, “To Improve the Strength and Resilience of the Empire State's Infrastructure.”^105^ The report recommended protecting transit systems, particularly tunnels, against severe flooding; investing in upgrades to transit infrastructure; expanding key transit networks to create transportation redundancies; and improving long‐term planning approaches, including interagency coordination and fund allocation.^105^

The MTA's recent and current five‐year programs (i.e., the 2010−2014, 2015−2019, and 2020−2024 capital programs) addressed many of the report's major recommendations.^106^ For example, the MTA's 2010−2014 Sandy Mitigation Program added new storm doors and drainage pumps at the Queens Midtown and Hugh L. Carey (Brooklyn‐Battery) tunnels, which serve the general vehicle fleet, including transit buses.^106^ Gates at the entrances to those tunnels can be manually shut prior to an impending flood and will provide a watertight seal. The MTA and New York City Transit have also fortified subway infrastructure against future storms, constructing seawalls and floodwalls and purchasing more than 3500 flood mitigation devices designed to seal off vents and other entrances to underground subway tunnels in advance of storms.^107^ The PANYNJ has taken action with its own subway, the Port Authority Trans‐Hudson (PATH) system, repairing stations that were heavily damaged by Sandy's storm surge and adding new flood‐proofing protection, including reinforced elevators, flood doors at stairway entrances, exterior flood walls, internal aquarium glass walls, and flexible fabric barriers.^108^, ^109^, ^110^, ^111^

General flood‐protection strategies include manhole inserts, strategic barriers, and other more targeted measures that protect critical, low‐lying infrastructure (e.g., the subway system) by enabling better control of stormwater and combined sewer overflow during storm surges and extreme flooding events. Increased pumping capacity in subway systems may be necessary for dewatering after flooding events. Long‐term measures include more permanent mitigation strategies such as constructing flood barriers and seawalls and elevating low‐lying infrastructure (e.g., rail lines, bus platforms and stations, subway entrances) where feasible. Electrical generation and distribution infrastructure can be made flood‐proof, and porous pavement can be used on bus routes in flood‐prone areas,^112^ though this would require interagency coordination, as roadways are the domain of city and state departments of transportation, not the MTA. In places where permanent inundation of mass transit infrastructure is projected to occur, managed retreat and rerouting of mass transit lines may be necessary. As storms increase in intensity, there will be a need to harden vulnerable mass transit stations and tunnels by increasing civic stormwater conveyance infrastructure, especially around station entrances.

Mass transit operators can also use adaptation strategies to avoid extreme heat impacts on infrastructure and protect passenger health. Cool pavements, which are composed of materials with higher solar reflectance that remain cooler than typical pavement, can be used on landings, platforms, and bus routes.^112^ Planting vegetation and constructing other forms of shade along transit routes and stations can also help reduce heat. Methods to control temperatures inside vehicles include painting bus roofs white to increase solar reflectance and tinting windows to increase shade. Some mass transit systems may also need additional air conditioning capacity.^82^ Installing misting systems and drinking water fountains at transit stations can also help protect human health.^82^, ^112^

Mass transit systems can also take steps to prepare for power outages, which may become more common as extreme heat and extreme wind events occur with more frequency. Power outages can affect charging infrastructure for electric bus fleets, leading to service disruptions. Resilience strategies for this issue include using backup power generators, connecting charging infrastructure to the grid at multiple points (allowing chargers to receive power even if an outage affects one part of the grid), and using battery backup storage as a power source for buses.^113^ The adoption of on‐board energy storage systems for trains could become important in a climate‐disrupted future, allowing trains to run without connection to a live power source for certain stretches along their route. This measure, already in use in several international rail‐based transit systems,^114^ would allow for the de‐electrification of particularly vulnerable segments of track, such as track running through under‐river tubes. Saltwater flooding of power systems in the tunnels under the East River caused extensive damage during Superstorm Sandy.^88^ Removing live power sources from such vulnerable sections would make them much more resilient, as trains would be able to resume service after flooding more quickly and at a lower cost.

#### 4.4.2 Municipal and regional government concerns

Municipal and regional governments will need to carefully assess their current planning approaches to climate‐related impacts on mass transit. Public transit agencies have traditionally treated extreme storms as emergencies and focused their resources toward developing emergency response plans.^115^ However, resilience requires proactive plans and long‐term adaptation strategies that anticipate climate change impacts. For example, the MTA's $55 billion five‐year (2020−2024) program includes numerous provisions to address climate‐related risks, including sea level rise, storm surge, changes in precipitation (e.g., more intense storms), temperature, and wind.^116^ The MTA's five‐year program initiatives aim to ensure that bridges and tunnels will remain resilient to climate events and that all new infrastructure projects incorporate resilience elements.

Public transit agencies face multiple barriers to incorporating adaptations in mass transit systems, including limited funding, aging infrastructure, and competing organizational priorities.^115^ Transportation agencies must also address other concerns that intersect with climate change, such as safety, emergency response, land‐use planning, and connectivity with other modes, which is why interagency action is an effective way to integrate climate change adaptation into transportation planning.^117^

The MTA's normal infrastructure replacement activities align with its climate resilience goals. As one example, the 2020−2024 five‐year plan includes an electrical substation upgrade at the Henry Hudson Bridge at Dyckman Street, which matches the power output from the Kappock substation located in the Bronx, ensuring full redundancy of the Henry Hudson Bridge facility and tolling equipment.^106^ The MTA has also funded drainage system projects to convey heavy rain runoff away from bridge supports and tunnels.

#### 4.4.3 Opportunities and cobenefits

Mass transit provides many cobenefits as it efficiently moves large numbers of people. Annually, the collective mass transit services overseen by the MTA reduce greenhouse gas emissions by 17 million metric tons, save $100 million in health costs from harmful emissions, and prevent 1.6 billion gallons of fuel from being consumed.^106^, ^118^ Public transportation is also considered a safer way to travel than operating a vehicle, with the chances of being in an accident decreased by over 90% compared with driving.^119^ Mass transit options provide sizable cost savings compared with taking a ridesharing or taxi trip,^120^ which benefits the many New Yorkers who do not own cars. Making mass transit infrastructure more resilient to climate impacts fortifies these benefits and presents opportunities to address other mass transit needs simultaneously. Increasing accessibility for people with disabilities, modernizing aging infrastructure, and improving reliability for users are additional challenges and goals for New York State transit operators.^29^ A transition to electric and other zero‐emission vehicles, such as bus fleets, would also require substantial changes to supporting infrastructure. For instance, the MTA has committed to transitioning its bus fleet to 100% electric by 2040, with an implementation timeline that includes adding 60 battery‐electric buses and supporting infrastructure at five bus depots by 2023.^121^

## 5 RAILWAY TRANSPORTATION

### 5.1 Background

#### 5.1.1 Mode description

Railways have served as a primary mode of freight and passenger transportation in the United States for nearly two centuries. In New York, rail transportation has been instrumental in the development, operations, and growth of the state.^2^ Today, the state has about 3300 miles of rail.^29^ Railways are primarily used as a means of moving freight quickly and efficiently over land, though rail systems also transport substantial numbers of people daily throughout the state and throughout the northeastern United States and eastern Canada.

As Figure 9‐4 shows, New York has freight rail service statewide, including in most of the state's counties and cities.^122^ Four large (deemed “Class I”) railroads—CSX Transportation, the Canadian National Railway, the Canadian Pacific Kansas City Limited, and the Norfolk Southern Railway—operate within New York, as do about 40 smaller railroads. The Class I railroads own and maintain most of the track infrastructure in the state, with CSX alone maintaining over 2800 miles of active tracks.^29^ Major freight rail facilities are located in New York City, Albany, Buffalo, Syracuse, and Binghamton, while smaller rail yards and facilities are scattered throughout the state.^122^ According to NYSDOT, 68 million tons of equipment, raw materials, manufactured goods, and produce are transported by rail in New York State every year.^77^

**FIGURE 9‐4:**
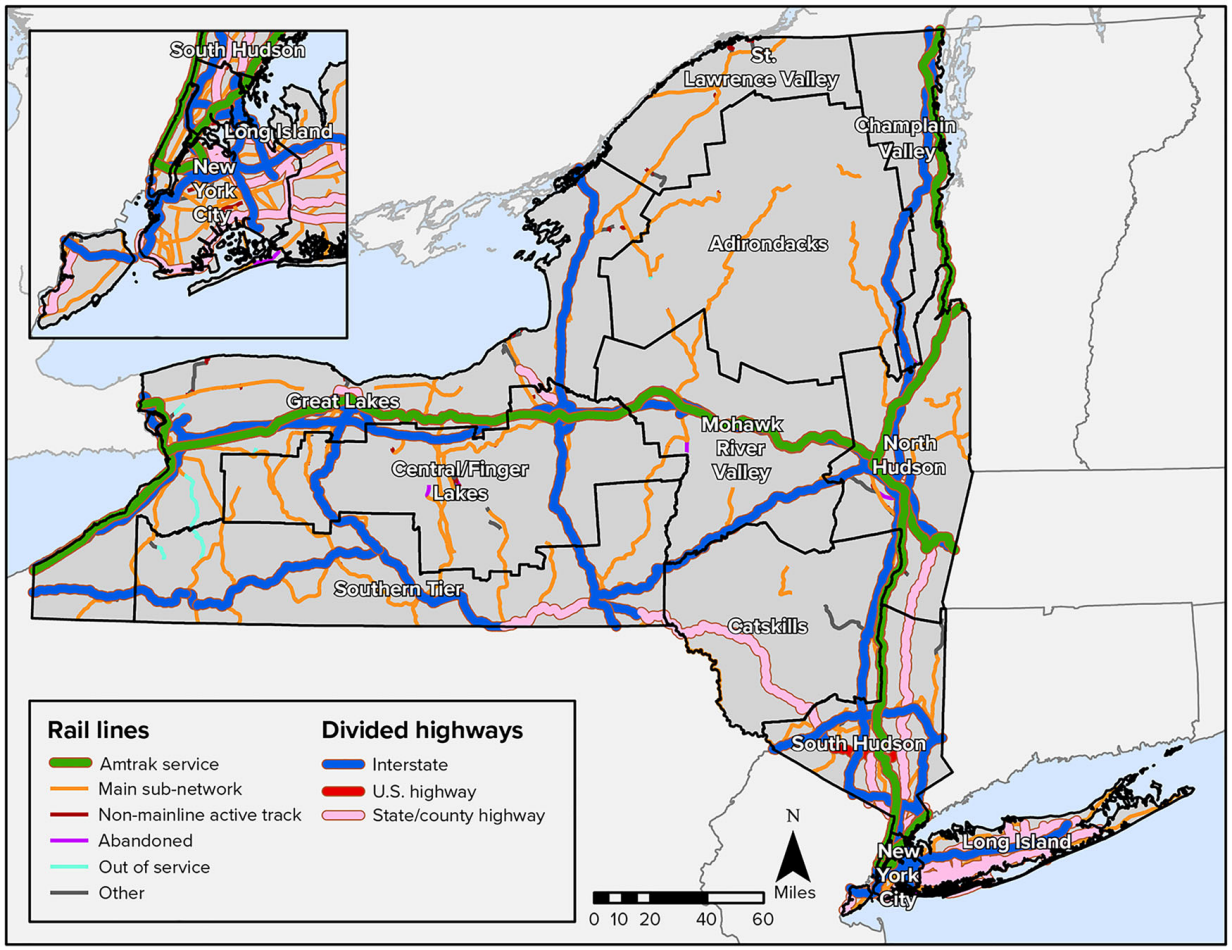
Railroads and major highways in New York State. Data from Bureau of Transportation Statistics (2019),^123^ Federal Railroad Administration (2021),^124^ and Federal Highway Administration and Esri (2023).^125^ Adapted from NYSDOT (2023).^126^

Long‐distance passenger rail service in the state is provided by the quasi‐public National Railroad Passenger Corporation, better known as Amtrak. Figure 9‐4 shows Amtrak's intercity passenger service, with routes running north−south (Montreal−New York City) and east−west (Boston−Buffalo). Amtrak also offers service along the Northeast Corridor route that connects New York City to Washington, DC, and Boston. Amtrak owns much of the track on the Northeast Corridor route, but elsewhere in the state, Amtrak generally operates on infrastructure owned by CSX and other freight railways.

Aside from some electrically powered Amtrak and commuter railroad lines in the New York City Metropolitan Area, nearly all trains operating in New York State, passenger and freight, are driven by diesel‐powered engines.^127^ Like many other states, New York has a sizable amount of overdue rail infrastructure maintenance, especially along the Northeast Corridor route. The maintenance backlog includes both basic infrastructure (like rail ties and electric wire) and major assets (such as the Pelham Bay Bridge, in the Bronx) that continue to be used beyond their useful life.^128^ This maintenance backlog is an existing stressor that may be exacerbated by projected climate impacts.

#### 5.1.2 Scope

This section considers climate change impacts on long‐distance passenger rail and freight rail. For the purposes of this discussion, passenger rail is defined mainly as intercity or interstate service, such as that provided by Amtrak. This differentiates passenger rail from mass transit systems with a metropolitan or regional scope, such as subways, light rail, and regional commuter rail, which are examined in Section 4. Rail infrastructure considered here includes trains, tracks, stations, bridges, tunnels, road crossings, intermodal terminals, and freight loading equipment. This section also considers impacts to the energy supply infrastructure for rail systems and to the labor force required to operate railways.

#### 5.1.3 Connections

Railway transportation connects to all other transportation modes. People reaching their destination by passenger train commonly transfer to other modes, such as mass transit, personal vehicles, or micromobility options. Freight rail has intermodal connections with maritime ports, airports, and depots that connect rail to highways. Because of these connections, climate change impacts to railways could disrupt other modes. For example, an impact to freight rail could delay the intermodal transfer of freight to and from ships and trucks, causing shipping backlogs. Extended disruptions to freight rail services could cause a temporary modal shift to trucks, leading to greater congestion on roadways and increased air pollution.

Railway transportation also intersects with other sectors of this assessment, including energy, human health and safety, and society and economy. Rail infrastructure relies on both electrical power and fossil fuel energy to operate, so climate‐related disruption to energy sources and distribution will create an indirect effect on rail transportation. Rail transportation intersects with human health and safety because of the potential for collisions at roadway crossings and the potential for the release of hazardous materials during rail accidents. New York State's economy depends on freight railways to move many goods and commodities, and intercity rail provides a vital link for people to access New York City and the surrounding regions, avoiding the congestion of the metropolitan area's roadways.

### 5.2 Observed and projected impacts

Climate‐related hazards such as flooding and extreme heat have already affected rail operations and infrastructure in New York State, and climate projections suggest increased risk in the decades ahead. Impacts can result in disruptions to service, damage to equipment, transit accidents, increased maintenance needs, and consequential downstream effects on communities, businesses, and the economy of the state.

#### 5.2.1 Impacts on the physical system

The following subsections provide an overview of how key climate hazards are expected to affect railway infrastructure and trains.

##### 5.2.1.1 Flooding

Increases in the frequency and intensity of extreme precipitation events may increase the risk of flooding across New York State, and sea level rise is expected to continue to increase the height and frequency of New York's coastal floods in future decades.^5^ Flooding impacts to railways include inundation of tracks and tunnels, damage to bridges and rail infrastructure, corrosion from salt water, infrastructure slope failure, and scour of bridges and embankments.^129^ Scour occurs when supporting soil and material around bridge foundations and embankments are washed away during a flood, which in the worst cases can lead to bridge collapse, track washout, and derailments.^129^ Water damage to electrical equipment, such as signals and switches, can cause failure, resulting in service disruptions or delays. For example, due to a signal power failure in 2018 that was attributed to weather‐related causes, the Northeast Corridor Amtrak trains in New Rochelle, New York, had to operate at restricted speeds, affecting 28 trains for a total of 255 train delay minutes.^130^ Power outages from flooding can also disrupt rail service for electrically powered trains.

##### 5.2.1.2 Extreme temperatures

The number of days above 90°F is projected to increase across New York State during the 21st century, and multiday heat waves are also expected to become more common.^5^ These changes can affect railways in a number of ways:

- Extreme heat can buckle tracks.^129^ When the track temperature rises above the expected operating temperature, the rails become weakened. The weight of train cars adds stress to these weakened areas, leading to deformations in the track called sun kinks.^95^ Compared with other parts of the United States, the Northeast, including New York State, is expected to experience the greatest railway impacts from temperature increases. This is due partly to the region's high density of rail networks and partly to the magnitude of its projected temperature increase, relative to the temperature at which the rails were originally laid.^95^
- Thermal expansion of railroad bridges and other structures can increase stress on building materials and lead to damage.^131^ Thermal expansion has affected New York State railway networks in the past. In 2018, for example, the West Onondaga Street railroad bridge in Syracuse partially collapsed in the middle of a record‐breaking heat wave, an incident that engineers attributed to the thermal expansion of concrete and steel due to high temperatures.^132^
- Extreme heat can cause catenary (i.e., overhead power lines, commonly used by electrically powered passenger trains) to expand, lose tension, and sag dangerously low.^129^ A recent Amtrak vulnerability assessment found that its network of catenary in the New York City Metropolitan Area will become increasingly vulnerable to extreme heat through the end of the century. The assessment noted that catenary produced the highest vulnerability scores for heat risks among all of Amtrak's Northeast Corridor assets.^133^
- Power outages related to increased regional demand during heat waves could disrupt electrically powered rail service.
- Extreme heat can lead to failures in signaling infrastructure, especially where it is housed in signal huts lacking climate control, which can reach internal temperatures of up to 130°F.^133^ Studies have shown that extreme heat has led to blown fuses and cable faults in signaling equipment.^134^

Snow, ice, frost, and freeze‐thaw hazards can cause damage to overhead lines and signaling equipment, freezing of switches, tunnel icing, and cracking and breakage of rails.^129^ These risks could decrease as extremely cold days become less common throughout the state during the rest of this century, although snowfall could increase in certain areas over the next few decades due to the lake effect. Projections of change in ice storm frequency are inconclusive.^5^

##### 5.2.1.3 Severe storms

Some types of severe storms, such as heavy rain and lake‐effect precipitation, are projected to become more frequent and intense in New York due to climate change; other types, such as tropical storms and hurricanes, are expected to become stronger, with more intense wind and rain.^5^ Fallen trees and windblown objects can result in downed overhead lines, structural damage, and track misalignment.^129^ Heavy rain can cause flooding, and wind can cause power outages, resulting in rail service disruptions. New York City−area Amtrak tunnels suffered $689 million in damage from Superstorm Sandy flooding in 2012.^135^ Inundation with seawater left behind salts that would have eventually damaged the tunnels’ cast‐iron and concrete linings if not removed. The salts also coated the railway tracks themselves, and severe cracks formed in the corroded bench walls of the tunnels. Both the bench walls and tracks had to be replaced.

#### 5.2.2 Impacts on the movement of people

In fiscal year 2022, 9,890,816 passengers boarded Amtrak trains in New York State—an average of 27,098 riders per day.^136^ Climate impacts on passenger rail lines, stations, and other infrastructure disrupt service of passenger trains and affect how people are able to move around the state and access education, jobs, health care, and other critical services. When a climate event does occur, the impacts are generally confined to passengers traveling along a particular track or to or from a particular affected station. Many long‐distance passenger routes on Amtrak include stops in New York City, making the rail tunnels, bridges, and stations in the city a key juncture of critical passenger rail infrastructure. Climate‐driven infrastructure failures in New York City can have cascading effects for travelers across the Northeast. For example, power outages in New York City in 2021 caused by Hurricane Ida caused substantial delays on some long‐distance Amtrak routes and cancellations on others.^137^ Such disruptions can leave passengers frustrated over lost time and can force them to incur unplanned costs for accommodation, alternative travel options, and food. For those who use long‐distance rail for commuting, frequent service disruptions and outages can impact jobs and income.

#### 5.2.3 Impacts on the movement of goods

Railways in New York State are used primarily for freight transport, and therefore, climate hazards that impact rail systems tend to have an outsized effect on supply chains and economic activity. As freight shipments are delayed by damaged or blocked routes or depots, cascading effects are felt across the economy. Further, most rail freight consists of raw or premanufactured materials and commodities, and railway disruptions directly impact the businesses that use these materials and commodities for manufacturing, food production, energy, and construction. Many hazardous materials are shipped by freight rail, which increases the risk of harm if derailments or crashes were to occur due to climate‐driven factors.

Freight railroad companies own nearly all the rail infrastructure on which they operate and invest substantial amounts of capital in system maintenance. Along with causing direct damage to infrastructure, extreme weather can accelerate normal track degradation and increase maintenance and repair costs for railroads. These costs are likely to be passed on to shippers, manufacturers, and consumers.

### 5.3 Vulnerable populations and systems

#### 5.3.1 Vulnerable populations

Railroad workers, particularly those working outdoors in rail yards, will be exposed to increasingly extreme weather, including extreme heat. Workers could also encounter damaged infrastructure, which could cause accidents.^138^

Cascading impacts from rail disruptions could affect workers at manufacturing plants and other facilities in New York State that depend on rail freight shipments. In 2009, for example, the Clearwater Paper Rail Bridge in Gouverneur, New York, was shut down due to unsafe conditions, and the Clearwater Paper Company had to use trucks to transport pulp.^139^, ^140^ The state‐funded reopening of the rail bridge and the resumption of rail service reduced transportation costs, kept the paper mill economically viable, and saved almost 100 jobs.^139^ Climate change impacts to rail infrastructure could cause similar supply chain disruptions and pose similar risks to businesses and jobs.

#### 5.3.2 Vulnerable sectors and industries

Among the businesses most vulnerable to climate impacts on rail transport are the freight rail companies themselves, as well as those New York industrial, agricultural, and commercial businesses that rely on railroads for freight transport. The food industry in the Central/Finger Lakes region developed in part because of its proximity to the Livonia, Avon & Lakeville Railroad.^139^ These businesses, which include Barilla Pasta, Kraft Foods, Archer Daniels Midland, Cargill, and Perdue, would be affected if climate hazards were to limit the railroad's ability to transport a high volume of freight. According to 2021 data, railways transported 24,900 carloads of food products that terminated in New York State, totaling 15% of all rail traffic that terminated in the state.^141^

The chemical industry, another major user of the rail network, shipped 40,000 carloads of chemicals by freight rail in 2019.^141^ Chemicals are considered extremely sensitive and critical cargo. In 2022, at a time when rail workers nationwide were threatening to strike, freight companies planned to halt chemical shipments prior to the strike date, which would have forced chemical facilities to shut down.^142^ When hazardous chemicals cannot be shipped via rail, they may be moved via trucks, resulting in potential safety concerns on the state's roadways.^143^ Climate change impacts on railways could disrupt the shipment of chemicals or increase the risk of accidents involving vehicles carrying hazardous chemicals.

The rail industry itself may face increased costs due to disruptions and damage. This challenge may be compounded by rail labor shortages, which can cause rail operations and maintenance to lag and can affect recovery from climate‐related events.^29^ Climate change is also projected to impact the expected lifespans of railway assets, a problem that requires consideration through asset management policies.^129^

#### 5.3.3 Vulnerable regions

Railways in the coastal regions of New York State are susceptible to sea level rise, storm surge, and tidal flooding, which can threaten low‐lying tracks and other rail infrastructure. Amtrak has identified sea level rise as one of the top threats to assets such as tracks and interlockings in the New York City area, with increased precipitation being another top threat.^133^ Saltwater corrosion of rail equipment and infrastructure is also a risk when saltwater inundation occurs in coastal regions. Electrically powered trains on Amtrak's Northeast Corridor route, which travels near the coast, face additional hazards not shared by diesel trains. Overhead power lines can be downed by extreme wind or affected by heat through excessive sag, and electrical equipment is vulnerable to flooding.^129^

Climate impacts on railways can differ between urban and rural areas. For example, the cities of Buffalo, Syracuse, Albany, Binghamton, and New York City are major rail freight hubs. At hubs, with their many interconnections, one set of climate‐related disruptions and delays can propagate to other parts of the rail system.^144^ The urban heat island effect increases the air temperature in cities and magnifies the impacts of extreme heat, increasing the vulnerability of urban railways to thermal expansion, overhead line sag, and track buckling.^144^ Rural areas also have concerns: for example, grade crossings with roadways tend to be more common in rural areas, whereas busy urban areas are more likely to have grade separation (e.g., overpasses). Of the nearly 2700 public grade crossings in New York State, 750 are passive grade crossings, which use signage instead of the flashing warning lights and gates found at active grade crossings.^145^ Passive crossings are especially dangerous during heavy storms, when visibility is reduced. Climate impacts could also affect active grade crossings—for example, through damage to electronic warning equipment caused by low temperatures or flooding.^129^

### 5.4 Adaptation and resilience

#### 5.4.1 Known methods

The following adaptations in the areas of policy, design, and retrofit can enhance the resilience of rail infrastructure:

- Consider climate projections in design criteria for retrofits, upgrades, and design of new rail infrastructure.
- Relocate critical rail infrastructure that is at risk of repeated damage and disruption due to climate‐related factors.^146^ For example, relocation may entail moving rail infrastructure outside of a floodplain. However, locating and preparing a new site may be challenging and expensive.
- Elevate the track, infrastructure, and key assets above current and future floodplains, considering sea level rise, future high tides, and flooding related to extreme precipitation.^129^, ^146^
- Dry floodproof buildings and stations by creating an impermeable barrier on the exterior to minimize the risk of water intrusion during flooding events.^146^ Floodproofing methods include waterproof layers, flood shields, reinforcement of walls and floors, backflow valves that prevent contaminated water from flowing back in, and pumps. Apply hydrophobic coatings to tunnel walls.^146^
- Wet floodproof infrastructure and buildings where possible to minimize disruption while letting water inundate the area in a controlled manner during a flood.^146^ Upgrade and increase the capacity of drainage to accommodate more intense precipitation events.^129^
- Create perimeter flood barriers at key water‐ingress paths.^146^ Inside tunnels, install shields for ventilation shafts and pressure‐relief ducts.^129^
- Upgrade rail bridges to protect foundations from floodwaters and to prevent bridge scour.^129^, ^146^ Performing routine inspections of rail bridges is an alternative regulatory adaptation strategy.^129^
- Install fencing and manage vegetation along tracks to control windblown debris during extreme weather conditions.^129^
- Paint tracks white or apply a specialized coating to decrease solar gain and reduce the risk of buckling due to thermal expansion.^129^, ^147^
- Prestress rails to prevent track buckling at high temperatures.^129^, ^147^ However, prestressing requires regular maintenance, and the prestress may be lost as rails age.
- Ensure backup power and redundancy in critical systems.

#### 5.4.2 Municipal and regional government concerns

Land use and congestion near rail yards is a potential concern for municipal and regional governments. In many cases, cities have grown around rail yards over time, limiting the land available for expansion today.^148^ These land constraints may limit where relocation of rail infrastructure can occur for climate adaptation purposes.

The maintenance of publicly owned infrastructure that interfaces with railways (e.g., bridges, grade crossings with public roadways, some passenger rail stations, etc.) could fall to municipal and regional governments. Aging infrastructure may require repairs and upgrades to enhance resilience.

#### 5.4.3 Opportunities and cobenefits

Rail is more fuel‐efficient than other modes commonly used to transport people and goods, so the use of rail inherently provides a relative climate benefit.^149^ Still, operators could look for ways to reduce the greenhouse gas emissions that result from rail's reliance on diesel fuel.^149^ As New York State makes further investments in rail infrastructure, the state has an opportunity to increase accessibility for people with disabilities and provide equitable access to affordable, reliable transportation options for people in underserved communities. Building resilient rail networks can help the state achieve its aims of reducing emissions and reducing reliance on overburdened modes, such as roadways, in the coming decades.

## 6 MARITIME TRANSPORTATION

### 6.1 Background

#### 6.1.1 Mode description

New York is the only state whose shorelines front on both the Atlantic Ocean and the Great Lakes. As a result, it has a robust waterway transportation system that includes marine and freshwater ports and terminals, as well as the New York State Canal System. The state's waterways help to move people (e.g., on commuter ferries, cruise ships, and other boats) and goods (e.g., on cargo ships and barges).

Ports and terminals throughout New York are operated by state and regional port authorities, regional economic development corporations, or private owners.^29^ PANYNJ manages various marine terminal properties in New York City and New Jersey. In 1962, PANYNJ opened the world's first container port, the Elizabeth‐Port Authority Marine Terminal in Newark.^150^ Today, the Port of New York and New Jersey accommodates the world's largest vessels and is one of the largest container ports in the country.^151^ In 2022, PANYNJ accounted for 16.4% of the entire U.S. container throughput market.^152^ PANYNJ also operates a major cruise ship terminal, the Brooklyn Cruise Terminal.^153^

The New York City Department of Transportation operates the Staten Island Ferry, while private operators such as NYC Ferry and New York Water Taxi provide services for the many other ferry routes throughout the city.^154^ Other passenger ferries cross Long Island Sound and Great South Bay, the Hudson River, and Lake Champlain. In addition to providing transportation within the state, ferries connect New York to New Jersey, Connecticut, and Vermont.

The Great Lakes Marine Transportation System (GLMTS) provides shipping access to the largest concentration of surface fresh water in the world. It includes Lakes Ontario, Erie, Huron, Michigan, and Superior, their connecting waters, and the St. Lawrence River. The system is operated by the Great Lakes St. Lawrence Seaway Development Corporation and serves 15 major international ports and 50 regional ports.^155^, ^156^ New York has four ports of note along the GLMTS: the ports of Buffalo, Oswego, Rochester, and Ogdensburg, in descending order of annual cargo tonnage.^157^

The New York State Canal Corporation, a state entity, manages and operates the New York State Canal System. The canal waterways span 524 miles and connect the Hudson River to the Great Lakes, the Finger Lakes, and Lake Champlain.^158^ The Port of Albany on the Hudson River is just downstream of where the canal system begins. The canal system has 57 locks, 17 lift bridges, and various marinas and public docks.^159^ Canalside communities benefit from the many waterway transportation opportunities on the canals, such as recreational boating, cruise lines, and some commercial shipping. Bike paths and heritage sites lie alongside the canals.^158^, ^160^

Figure 9‐5 shows key ports in New York State along with several water routes that are federally designated as marine highways.

**FIGURE 9‐5:**
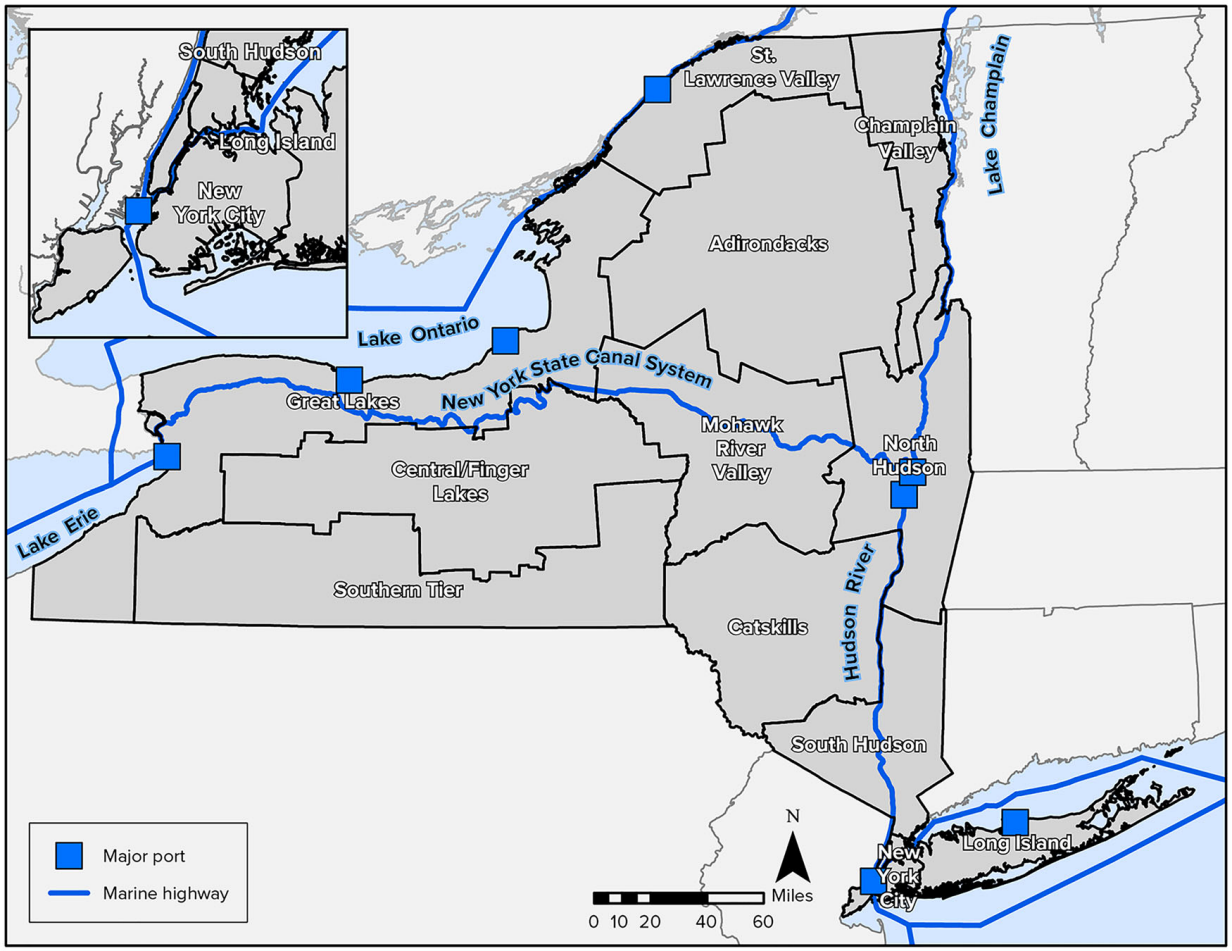
New York State's major ports and marine highways. The state is served by four formally designated marine highways, which are water‐based corridors for moving freight by ship.^148^ Data from U.S. Maritime Administration (2023),^161^ U.S. Army Corps of Engineers (2023),^162^ and National Atlas of the United States (2014).^163^

#### 6.1.2 Scope

This modal section covers the cargo ships, tugs, barges, cruise ships, boats, and ferries that traverse the marine waters and inland waterways in New York State. It also considers associated infrastructure:

- Harbors, marinas, and docks where vessels can be moored and secured.
- Cargo ports and terminals, which have specialized infrastructure, such as cranes, shore‐side vehicles, and other equipment, along with intermodal road and rail connections.
- Sea walls, flood gates, and other barriers that help regulate the flow of water to protect marine and waterway infrastructure.
- Bridges and tunnels that allow ground transportation to cross over or under waterways, and lift or drawbridges that can be raised to allow tall watercraft to clear the bridge as they pass.
- Locks that raise or lower watercraft as they travel from one stretch of a waterway to the next. For example, a series of locks on the St. Lawrence River raise ships more than 200 feet in elevation as they travel between Montreal and Lake Ontario.^164^
- Other systems, including traffic systems and fuel distribution and delivery systems.

While ferries are often considered mass transit, the assessment covers them with maritime transportation because they share key attributes and climate risks with other types of ships and boats.

This section examines the effects of climate hazards on the infrastructure that supports major maritime transportation systems spread across the state, including the GLMTS, the Port of Buffalo, and the Port of New York and New Jersey. Although much of the Port of New York and New Jersey is located on the New Jersey side of New York Harbor, this section assesses climate change impacts to the entirety of the port system, as the port system is the largest component of New York State's maritime trade and is governed by a bi‐state authority, PANYNJ.

#### 6.1.3 Connections

Maritime transportation intersects with other modes of transportation covered in other sections of this chapter. Ports are intermodal hubs with roadways and rail connections, while ferry terminals in New York State primarily connect to roadway, mass transit, and micromobility options. Hazards that disrupt water transport or port operations can create congestion in other modes as people seek alternatives.

Water‐based transportation also intersects with other sectors of this assessment. For example, ports play a critical role in international trade. The Society and Economy chapter of this assessment provides greater detail about how climate events can disrupt global supply chains, including the import of parts for manufacturing. Marine transportation also intersects with energy, as New York State's supply of petroleum fuels depends in part on tanker ships and the terminals where they unload. The Energy chapter provides additional analysis of how marine terminal disruptions can affect the distribution of petroleum fuels.

### 6.2 Observed and projected impacts

Notable climate‐related hazards for maritime transportation in New York State include extreme storms, coastal flooding, sea level rise, changes in precipitation patterns, rising temperatures, and changes in wind and wave conditions.^165^ Climate hazards affecting inland waterways such as the Great Lakes, the St. Lawrence Seaway, the New York State Canal System, and the Hudson River include rising air temperatures, increased precipitation, and riverine flooding. Riverine flooding is typically a result of heavy precipitation, whereas coastal flooding's main drivers are tidal fluctuations, storm surges, sea level rise, and their interplay.^166^ These hazards affect the physical systems and vessels that maritime transportation relies upon, with the potential to disrupt the movement of goods and the movement of people across the state's bodies of water.

#### 6.2.1 Impacts on the physical system

The following subsections provide an overview of how certain key climate hazards are affecting maritime transportation infrastructure and vessels.

##### 6.2.1.1 Sea level rise

The most devastating impacts to maritime transportation infrastructure are expected to come from storm surge, which will reach higher and farther inland as sea level rises.^167^ As described in Section 2.2, sea level is projected to rise along New York State's coastline by up to 1 foot by the 2030s, by about 2−3 feet by the 2080s, and by more than 4 feet by 2150. Sea level rise increases the risk of overtopping, which occurs when waves rise over sea defense structures, and increases the exposure of piers and wharfs to waves.^165^

Rising sea level could necessitate a shift from roll‐on/roll‐off port facilities to lift‐on/lift‐off port facilities in harbors. Roll‐on/roll‐off port facilities rely on vehicles to roll cargo on and off a vessel, but they may no longer be effective at higher water levels. In contrast, large tidal variations do not affect lift‐on/lift‐off port facilities, which use cranes to load and unload a vessel.^168^

The safe movement of ships in and out of ports depends in part on wave height and patterns.^169^ Sea level rise increases the water depth in and around harbors, where waves are depth‐limited, potentially leading to higher waves.^169^ Substantial increases in wave height can limit ship maneuverability and operations.^165^ Sea level rise and flooding can also result in reduced clearance under bridges, preventing vessels from traveling under bridges and raising the risk of vessel‐bridge collisions.^170^

##### 6.2.1.2 Severe storms

Tropical storms, including hurricanes, are among the most dangerous and disruptive climate hazards affecting ports in New York State.^165^ While it is not clear whether the number of hurricanes that reach the state will increase, those that do affect New York are projected to be more intense.^5^, ^171^ High winds, heavy rain, and storm surge flooding from tropical storms can damage port facilities and electrical infrastructure, disrupting operations and causing fuel shortages and prolonged power outages. Disruptions to oil terminal operations during a hurricane can further contribute to fuel deficits, exacerbating storm‐driven impacts to shipping and other transportation modes that connect at ports. High winds during storms can damage vessels and make maneuvering, securing, loading, and unloading them more challenging, dangerous, and costly.^165^, ^167^ Extreme weather can cause a temporary shutdown of ports and worsen sailing conditions.^170^ Port security can also be impacted by extreme storms when facility infrastructure and security equipment become compromised.

At the Port of New York and New Jersey, most operations were shut down for nearly a week in the fall of 2012 due to damage and energy supply disruptions inflicted by Superstorm Sandy.^172^ Wind and storm surge damaged vessels, berths, and equipment. The storm toppled shipping containers into the harbor and created a floating debris field that blocked vessels. It also knocked out power at the port for several days and forced the port's many oil terminals to cease operations, causing a regional fuel shortage.^172^ PANYNJ reported about $170 million in losses from Superstorm Sandy, $130 million of which was in capital.^172^

Hurricanes and storms also disrupt ferry services and other maritime passenger systems. For example, Superstorm Sandy caused the Staten Island Ferry to suspend service for 5 days as a result of debris wash‐up and flood‐related electrical damage to terminals.^172^, ^173^ The Staten Island Ferry carries tens of thousands of passengers on a typical weekday (45,000 as of 2023),^174^ so this 5‐day service suspension affected many thousands of potential passengers.

Other types of storms can also affect maritime transportation operations. Nor'easters tend to produce weaker winds and less storm surge flooding in New York than hurricanes but can last longer. While projecting climate change implications for nor'easters remains difficult, the amount of precipitation associated with a nor'easter is expected to increase.^5^, ^175^ Meanwhile, Great Lakes ports may have to contend with disruptions from more frequent and more intense storms over the next few decades due to an enhanced lake effect with warmer water and less ice cover.^5^ Heavy precipitation limits visibility for ship operators,^165^ requiring reductions in speed, causing delays, and heightening the risk of collisions.^167^

##### 6.2.1.3 Heavy precipitation

Total annual precipitation is projected to increase through the 21st century across all parts of New York State, as is the frequency of days with more than 1, 2, or 4 inches of rain.^5^ Increased precipitation reduces port capabilities for loading and unloading cargo by decreasing visibility, increasing safety hazards for port workers, and preventing the loading or unloading of cargo that cannot get wet, such as grain, cement, and machinery.^176^ Increased precipitation also contributes to the corrosion of port equipment, vessels, and infrastructure.^170^

Increased precipitation and heavier storms raise the risk of flooding in New York State's rivers and canals.^5^ Riverine floods can disrupt inland waterway transportation and damage port facilities.^177^ They can also accelerate the accumulation of sediment in a river and change the shape of the river channel, which poses a hazard for navigation.^177^

Rainy conditions have been shown to negatively affect ferry ridership more than bus or train ridership—possibly due in part to exposed waiting areas and walkways.^178^

##### 6.2.1.4 Rising temperatures

Higher air and water temperatures could increase evaporation in the Great Lakes, which would lower surface water levels and counteract the effects of increased precipitation. Projections of future lake levels are limited, with some conflicting and uncertain results, but there is general agreement across climate models that annual and multiyear variability in Great Lakes water levels will increase in the decades ahead.^5^ If warming and drought were to lower lake levels, it would restrict vessel drafts (the vertical distance between the waterline and the lowest point on the bottom of the hull) and force shipping companies to limit their loads so that their vessels would ride higher in the water, maintaining minimum clearance. More trips would be needed to transport the same amount of cargo, resulting in higher costs.^179^, ^180^ One study found that if the draft of a vessel is decreased by 3 feet, the cargo capacity of the vessel is reduced by 15%.^180^ As a result of shallower water depths, Great Lakes shipping costs could increase by 30%.^168^ For some types of cargo, alternative modes can be used for shipping and may become more competitive if water transport across the Great Lakes becomes too costly.^180^ Conversely, if water levels were to increase—a possibility that has been suggested for certain seasons (late winter through early summer)^5^—it could lead to impacts analogous to those described above for sea level rise.

In rivers, drought can cause barge shutdowns due to low water levels.^167^ While New York State is expected to experience long‐term increases in precipitation, short‐term seasonal droughts could become more common, partly as a result of higher temperatures in the warmer months.^5^ Short‐term droughts would bring the potential for lower river levels.

Ice coverage on the Great Lakes and canal systems hinders overwater transportation. Large ice sheets that form on the surface of the water must be broken by icebreaking vessels to allow the safe passage of other vessels. This can lead to transportation delays and increase shipping costs, as icebreaking vessels are expensive to operate and add time to the journey. With temperatures projected to rise in New York State, ice cover is projected to continue to decline, allowing for more efficient overwater transportation during the winter.^5^, ^168^

Port infrastructure and vessels are also impacted directly by increases in average air temperatures. Rising air temperatures can affect refrigerated cargo ships by reducing their capacity to refrigerate goods and increasing refrigeration challenges and costs.^170^ Extreme heat can also soften or melt asphalt at port facilities, challenging the movement of heavy cargoes and the operation of cargo‐handling equipment, or accelerating the deterioration of wharfs, docks, and terminal lots.

#### 6.2.2 Impacts on the movement of people

Maritime passenger transport in New York State is centered around ferries. Where roadways and mass transit provide modal redundancy, climate‐driven impacts to ferry operations are expected to have only a limited effect on the movement of people. However, Staten Island residents could experience substantial impacts from climate‐driven service disruptions, as the Staten Island Ferry is the island's only direct connection to Manhattan. Cruise ship operations may also be disrupted by climate‐driven impacts to terminals in New York City, with cascading impacts such as lost revenue for the businesses in the city that serve the cruise industry and its customers. Increased temperatures have the potential to increase ridership on the city's ferries, as overwater transportation offers cooler temperatures than other transportation modes in urban areas. New York City's public and private ferries also provide transportation to beaches, with ridership spiking during summer months and heat waves. For example, during a 2‐day heat wave in June 2017, ferry ridership from Wall Street to Rockaway increased by more than 30% when compared with the 3‐day Memorial Day weekend a few weeks prior.^181^

#### 6.2.3 Impacts on the movement of goods

Cargo‐bearing vessels serve ports along the Atlantic Ocean, the Great Lakes/St. Lawrence Seaway, and the Hudson River. While climate‐driven disruptions can occur as vessels travel between ports, especially during storms, most impacts to the movement of goods happen during port‐specific intermodal connections, when cargo is transferred between ships and railways or trucks. Intermodal transfer of goods cannot occur when ships cannot dock due to hazardous conditions or when trains and trucks cannot access the cargo transfer areas of the port. During Superstorm Sandy, for example, PANYNJ activities were shut down for 24 h prior to the storm making landfall to finalize storm preparations.^182^ The storm damaged critical infrastructure at the port, including ship berths, piers, operations centers, transformers, cranes, oil storage tanks, fuel‐oil pumps, security systems, and transportation infrastructure. Although some critical services (such as the operation of sewage‐transfer vessels) and limited water taxi services resumed within 24 h after the storm passed, the port was closed to most tug and barge traffic and deep‐draft vessels for 3−5 days.^182^ Some of the damage took more than a year to repair. Such impacts can be costly, as they affect supply chains and the movement of goods to markets. They can also create a cascading effect where cargo deliveries are delayed throughout the state.

### 6.3 Vulnerable populations and systems

#### 6.3.1 Vulnerable populations

Populations vulnerable to the impacts of climate change on maritime transportation include workers, near‐port communities, and people who commute by ferry. Port and maritime workers will face growing hazards from extreme heat, as the number of days above 90°F is expected to increase statewide during the 21st century, as is the number of heat waves.^5^ According to the Occupational Safety and Health Administration, outdoor temperatures above 77°F can create unsafe working environments for individuals performing strenuous work, and such temperatures also create a high risk of heat‐related illness for unacclimated workers.^183^ Workers in ship engine rooms and cargo storage areas may be especially vulnerable, as the combination of external climate conditions and the heat given off by engine components can put them at risk of heat stress.^184^ The Human Health and Safety chapter provides additional information about heat‐related illnesses, while the Society and Economy chapter discusses how increased heat can also decrease worker productivity as a result of both hot conditions and the measures taken to mitigate them, such as more frequent breaks.

Near‐port communities experience elevated exposures to air pollution from marine traffic. A study by the New York City Health Department found that areas with heavy marine traffic had high levels of air pollutants associated with ship emissions.^185^ Climate‐related port disruptions can cause shipping congestion in harbors, extending the time that ships, as well as trucks at the intermodal transition from sea to land, spend idling and contributing to air pollution. These pollution impacts will disproportionately affect communities living closest to marine terminals, which are often low‐income communities or communities of color.^186^

Commuters are particularly vulnerable to climate change impacts on waterway transportation. Waterfront communities that are underserved by other modes of mass transit may rely on ferries and boats, which connect waterfront communities to one another.^187^ In 2021, 43% of people commuting via NYC Ferry identified as essential workers.^188^ Some New York residents near Lake Champlain also use ferry services for commuting or job‐related travel.^189^

#### 6.3.2 Vulnerable sectors and industries

Climate‐related disruptions to maritime transportation services could affect a wide range of economic sectors and industries, including sectors that ship cargo by sea or inland waterway. For example, the Port of New York and New Jersey's top cargo imports by volume are furniture, machinery and appliances, plastic, and beverages.^190^ The top cargo exports are wood pulp, vehicle parts, plastic, wood, and articles of wood.^190^ The major exports of the Port of Albany include scrap iron, manufactured wind turbine components, and generators and power systems equipment. The Port of Albany also exports wood pulp to paper mills in the region.^191^ The Ports of Buffalo, Oswego, and Rochester distribute construction materials such as sand and cement to local suppliers, and municipalities in the Great Lakes region depend on lake transportation to deliver salt for winter road safety.^192^ Many New York State farmers deliver grain to global markets via the Port of Oswego's grain export center.^193^

Disruptions that occurred during the COVID‐19 pandemic can provide insight on how shutdowns of maritime transportation services can affect the economy. PANYNJ lost $3 billion in revenue between March 2020 and March 2022 as ports were forced to limit activity.^194^ The pandemic caused thousands of port workers to lose their jobs temporarily or permanently, forced ferry shutdowns and restrictions that limited ridership, and reduced or limited access to ports. Although COVID‐19 affected maritime transportation operations differently from the way a climate event would, the pandemic showed how supply chain disruptions can create economic stresses.

Climate change impacts to ferry operations will affect the local and regional distributors in New York State that use ferries. For example, some distribution companies send their delivery trucks on the Lake Champlain ferry between Plattsburgh, New York, and Grand Isle, Vermont, because of the convenience, shortened travel time, and fuel cost savings.^195^ During climate‐related ferry shutdowns, these businesses will be forced to take less direct routes by road.

The New York City Department of Sanitation uses barge transport to move much of the city's municipal solid waste from marine transfer stations to locations outside the city for treatment or disposal.^196^ Barge infrastructure at intake terminals and end‐point waste facilities is vulnerable to flooding and corrosion from storms and sea level rise, which could result in contamination of water bodies and cause waste backups within the city.^197^

New York State's tourism and recreation industries use maritime transportation and are vulnerable to climate change impacts, too. For example, the Staten Island Ferry is popular with tourists, and the New York Water Taxi operates sightseeing services in addition to its shuttle service.^187^ Ferry service delays and shutdowns resulting from extreme storms and flooding will disrupt the movement of tourists. Additionally, the New York State Canal System is primarily used for tourism and recreation today, with the waterways attracting thousands of recreational boaters.^198^ Flooding could damage infrastructure that supports recreational boating, such as marinas and docks.

#### 6.3.3 Vulnerable regions

Several parts of New York State have substantial commercial vessel traffic. Each has its own climate vulnerabilities:

- In the Great Lakes and the St. Lawrence Seaway, decreases in water levels could pose a threat to the economics of shipping by reducing the weight that cargo vessels are able to carry. Rising water levels could also pose challenges.
- In the coastal region around New York City, shipping terminals and vessels that move millions of tons of cargo face potential disruptions from severe storms and sea level rise, and ferry services are also at risk.
- In the Hudson River, sea level rise and coastal storm surges can propagate up to the Federal Dam in Troy,^78^ flooding low‐lying areas and threatening port and marina infrastructure.
- In Lake Champlain, high water levels from storms and extreme precipitation events can disrupt ferry service.^189^

### 6.4 Adaptation and resilience

#### 6.4.1 Known methods

Methods of adaptation and resilience for maritime transportation primarily involve changes to port, terminal, and marina infrastructure. Port infrastructure typically has an expected lifespan of 20−50 years, and therefore, should be designed to withstand multiple decades of climate impacts.^165^ In response to a changing climate, port managers may need to plan the replacement of current infrastructure sooner than they typically would based on a normal expected lifespan. A key step to establishing port resilience is to develop construction codes and guidance for waterfront design.^165^, ^199^

New York State is already working to upgrade marine and waterway transportation infrastructure. PANYNJ, for example, has published science‐based Climate Resilience Guidelines that exceed existing codes and standards and ensure that infrastructure design considers future climate risks.^200^ To prepare for sea level rise and increased coastal flooding, the New York City Economic Development Corporation and the Mayor's Office of Climate Resiliency released the *Financial District and Seaport Climate Resilience Master Plan* in 2021. This plan calls for elevating terminals and piers as well as critical electrical and mechanical systems, using flood‐resistant materials, and constructing flood defense systems.^201^ Other important infrastructure for marine shipping, such as access roads, rail tracks, and warehouse facilities near ports, might also need to be elevated to increase resilience to sea level rise and coastal flooding.^78^

Because bridge clearance will decrease with sea level rise, raising the height of bridges may be necessary to ensure that vessels will be able to pass underneath them in the future.^78^ In 2019, the Bayonne Bridge in the Port of New York and New Jersey was raised to boost trade by allowing larger ships to enter the port.^202^ Raising bridges in a similar manner to adapt to sea level rise would enable large ships to continue transporting goods.

Though there is no scientific consensus about the future of Great Lakes water levels, raising or lowering docks and other infrastructure in New York ports on Lakes Erie and Ontario could become necessary if water levels change markedly.^180^ Elevating or lowering infrastructure in response to water level changes across all New York waterways and coastal areas could be an effective, though expensive, adaptation strategy.

If a decline in Great Lakes water levels were to occur, harbor and channel dredging, which removes sediment and other materials from lake bottoms, is a potential strategy to increase the draft clearance of the shallowest parts of shipping lanes. Historically, dredging has been conducted to counteract low water levels. During an episode of low water levels that began in 1963, dredging activity in the Great Lakes increased 10‐fold over a 5‐year period.^203^ However, dredging can have harmful environmental impacts as it can disturb lakebed sediments that are contaminated with heavy metals, mercury, and polychlorinated biphenyls.^168^

Technical advancements in maritime transportation also offer promise as a means of adapting to climate hazards. One example is the use of artificial intelligence and data analytics for cargo and port arrangement optimization, smart propulsion systems, and digital route management.^204^ These technologies can help shipping vessels and ports respond to extreme storms, changes in precipitation patterns, and rising temperatures by altering shipping routes and docking arrangements based on real‐time weather data measured by on‐board monitoring systems.

Adaptation for maritime transportation can benefit from “resilience alternatives”—a set of actions tailored to decision‐makers for encouraging wide buy‐in.^205^ Although resilience alternatives can involve investing in physical mitigation measures like those described above, they can also include updating plans and procedures, holding employee workshops and community outreach seminars on planned resilience measures, or allocating a budget to future resilience initiatives. Each port, terminal, or marina has its own intricacies that will require individualized approaches to maximize adaptation and resilience. Metrics such as the ratio of recovery time to degree of damage, network functionality, or intermodal connectivity can help operators identify the most critical systems to prioritize.^205^

#### 6.4.2 Municipal and regional government concerns

Ferries can provide mobility and connectivity for local communities, which is especially critical for routes that lack a nearby road or rail alternative (e.g., ferry crossings of Lake Champlain or the direct ferry connection from Staten Island to Manhattan). It will be in the interest of municipal and regional agencies to ensure that these ferry services are resilient and able to keep people moving in support of the local economy.

Port resilience is also important to municipal and regional governments, as ports can contribute substantially to local and regional economic activity and employment. Adaptation projects can create their own forms of disruption—for example, the traffic and mobility impacts of closing roads and bridges to elevate them—so it will be important for port operators to plan collaboratively with adjacent communities. Local planners might also have to manage competing land uses as cargo terminals vie with commercial developers and others for space along a changing coastline.

#### 6.4.3 Opportunities and cobenefits

While changing climate conditions are expected to have multiple negative impacts to marine and waterway transportation, they may also produce new opportunities. In addition, the work of building resilience to climate impacts could generate cobenefits for this transportation mode.

Climate change could spur increased use of maritime transportation in some situations. For example, in waterfront communities, ferries can be used when other modes of transit shut down, because ferries are not dependent on the electric power grid or limited to tracks.^206^ When extreme storms and flooding disrupt adjacent modes, ferries can still operate so long as terminals are unaffected and waterways are calm enough for service. For months after Superstorm Sandy, New York City and the Federal Emergency Management Agency implemented emergency ferry services while land‐based mass transit was recovering from the disruptions from the storm.^172^ In the future, the redundancy offered by marine and waterway transportation may become a key component of resilience planning.

In some areas of the state, climate change may extend the season during which waterway transportation is possible. During the winter, operations in the Great Lakes and in New York's canal system have traditionally been restricted due to ice conditions. Under a warming scenario, waterway navigation may be possible year‐round, increasing, for example, trade opportunities via the Port of Buffalo and recreational usage through the canals. Additionally, certain costs that result from extreme winter weather could decrease. These include costs associated with icebreaking, damage to vessels and infrastructure caused by floating ice, and delays from ships trapped in ice.^168^ More broadly, new transportation patterns may emerge under warming conditions—for example, Arctic shipping routes may open due to reduced sea ice conditions,^207^ which might divert traffic away from or bring new traffic to New York.

Conversely, a shift to year‐round waterway transportation could bring new challenges. As noted in the 2011 ClimAID assessment, the winter season has traditionally been used to conduct lock maintenance at the Welland Canal, which connects Lake Ontario and Lake Erie. If year‐round navigation becomes possible and operations at the canal are no longer suspended during winter, then increasing lock capacity in each direction may be necessary so shipping is not disrupted by lock maintenance.^78^

Efforts to connect vessels to shore power when alongside the berth reduce the need for vessels to keep their own engines running, which can reduce local air pollution as well as emissions of greenhouse gases that contribute to climate change. Plans for sustainable energy updates to ports and terminals include onshore power connections and other clean‐energy technology applications, such as hydrogen fuel cells to power heavy‐duty port equipment and trucks. On‐site electric power generation could create useful backup power during grid outages driven by extreme weather.

## 7 AIR TRANSPORTATION

### 7.1 Background

#### 7.1.1 Mode description

Aviation provides quick domestic and international connections to move people and goods. New York State has more than 500 public and private aviation facilities, 131 of which were active public‐use facilities as of 2018 (Figure 9‐6). These include 19 commercial service airports, 101 general aviation airports, 5 heliports, and 6 seaplane bases.^208^ In 2019, nearly 55.5 million people boarded an aircraft at one of the state's commercial airports, an increase of 1.6% from 2018.^209^ A combined total of 94.2 million passengers traveled through three airports operated by PANYNJ within New York State: John F. Kennedy (JFK) International Airport, LaGuardia Airport, and New York Stewart International Airport.^210^ Another 46.3 million traveled through Newark Liberty International Airport, which serves the New York and New Jersey metropolitan areas.^210^ In addition, approximately 3.4% of all U.S. cargo and mail freight is handled through New York State airports, with most of it moving through JFK, which is one of the busiest airports in the country in terms of the volume of cargo handled.^211^

**FIGURE 9‐6:**
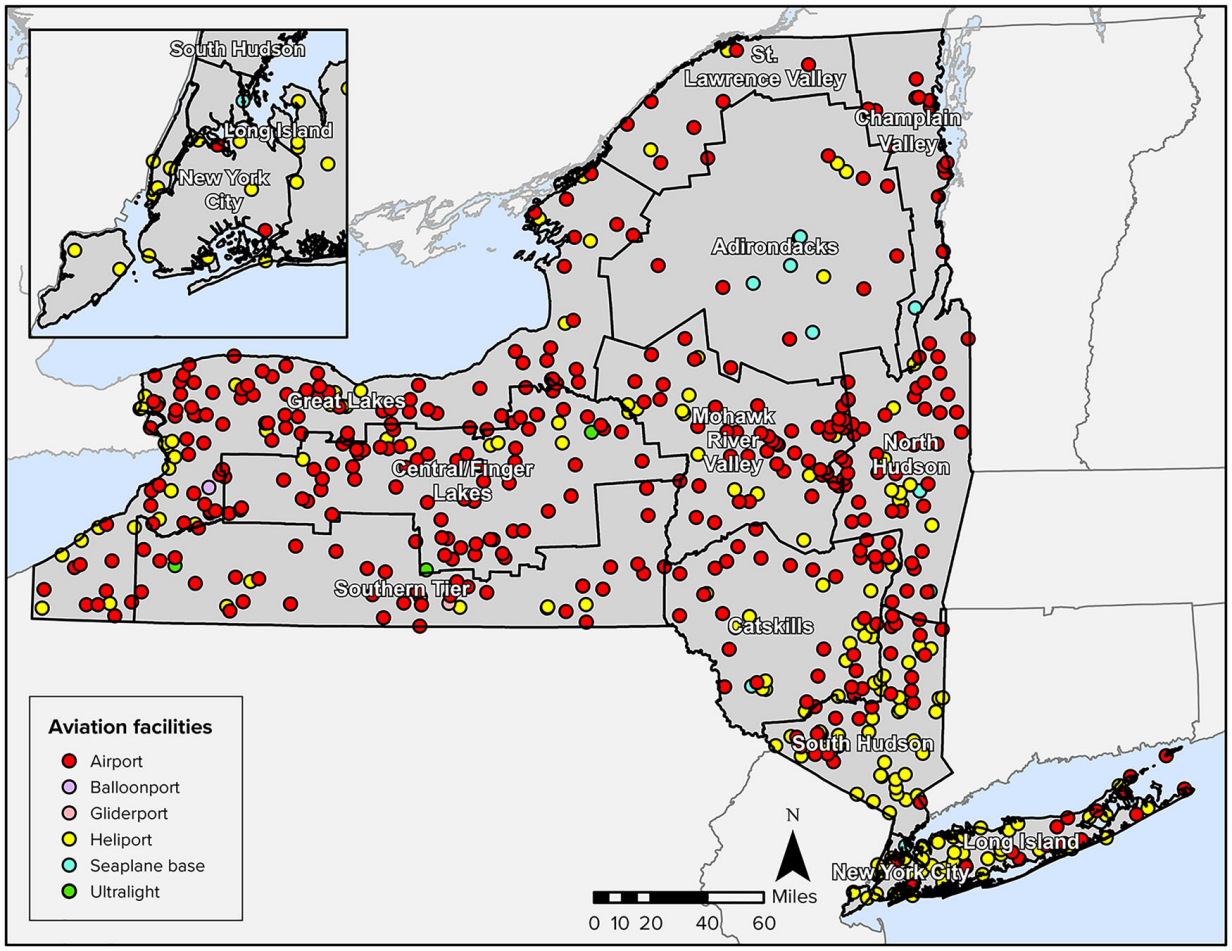
Aviation facilities in New York State. Data from Federal Aviation Administration and Esri (2023).^213^

Airports provide many modes of employment and facilities for people. Beyond direct aviation support activities, for example, many commercial airports provide access to retail stores and restaurants, office space, and other services. NYSDOT reported that in 2019, airports across the state generated nearly $80 billion in economic activity, 454,500 jobs accounting for $27.5 billion in payroll, and $6.1 billion in state and local tax revenues.^211^ These figures illustrate the importance of the aviation industry to the state in generating income, providing jobs, and contributing to the tax base of the communities it serves.^208^

#### 7.1.2 Scope

This section covers all types of aviation facilities, from the international, national, and regional airports that most travelers are familiar with to specialized facilities such as seaplane bases and heliports. Built infrastructure can include terminals, hangars, parking garages, air traffic control towers, fuel storage and refueling infrastructure, maintenance sheds, warehouses, intermodal cargo depots, radar and telecommunications systems, and sewage treatment plants.^208^ Runways, taxiways, and helipads are also important forms of infrastructure.^212^ This section also considers impacts to ground support vehicles that move people and goods around airports, including shuttles, trains, baggage conveyance systems, and runway vehicles, as well as impacts to the workers who operate them.

#### 7.1.3 Connections

Air transportation depends on, and is integrated with, several other modes of transportation. Climate change impacts on these other modes can have important consequences for air transportation, and vice versa. For example, disruptions to roadways and mass transit systems caused by storms or flooding can make it difficult for travelers and workers to access airports.

Air transportation also intersects with other sectors in this assessment. For example, the human health and safety sector relies on air transportation to connect communities and residents with emergency preparedness and response services, including aeromedical flights, law enforcement, national security, search and rescue, and disaster relief. In the agriculture and buildings sectors, aircraft are used for functions such as aerial application of fertilizer and pesticides and aerial surveys for the development of maps and infrastructure plans. Increased requirements for air conditioning at aviation facilities will increase energy demand. Conversely, disruptions to the electric power grid could cause power failures at airports and disrupt air traffic communications and ground operations, particularly as ground operations become more electrified.

### 7.2 Observed and projected impacts

New York's air transportation sector is susceptible to a variety of climate hazards, including rising temperatures, sea level rise, and severe storms. These hazards will result in impacts to physical air transportation systems and may disrupt aviation operations across the state, causing delays in the movement of both people and goods. Although PANYNJ is investing in protection for critical airport assets, the risks from climate events that cause storm surge and flooding are particularly high for coastal airports in the New York City Metropolitan Area.

#### 7.2.1 Impacts on the physical system

While the impacts of the aviation industry on the environment have been well documented,^214^, ^215^ impacts from climate change also affect air transport operations and economics. Climate hazards such as sea level rise, extreme weather events, and rising temperatures pose risks to airport infrastructure and airline operations. Airports and aviation infrastructure at lower elevations are vulnerable to increased storm surge and coastal flooding, while an increase in precipitation and severe storms may lead to more flight delays and cancellations.^78^ The following subsections provide more detail.

##### 7.2.1.1 Sea level rise and coastal flooding

As described in Section 2.2, sea level is projected to continue rising along New York State's coastline throughout this century and beyond. Rising sea level increases the potential for storm surge to reach low‐lying coastal airports. Floodwaters can inundate ground transportation and runways^216^ and can also damage or destroy facilities and equipment such as airfield vehicles. The three major airports that serve the New York City Metropolitan Area—JFK, LaGuardia, and Newark Liberty—are all within 21 feet of elevation above current mean sea level,^217^ and Superstorm Sandy produced storm surges that flooded parts of each. Had Flushing Bay not been at low tide when the storm struck, it is likely that LaGuardia would have experienced extensive terminal flooding instead of simply jetways underwater.^218^ With the sea level projected to rise by 2−3 feet during this century,^5^ storm surges in Newark Bay, Jamaica Bay, and Flushing Bay will increasingly threaten the New York City area's air traffic.

##### 7.2.1.2 Severe storms

Severe weather events can cause substantial impacts to air transportation operations and infrastructure. Extreme storms such as hurricanes, nor'easters, and lake‐effect blizzards cause operational impacts such as flight cancellations and airport closures. Increases in the severity of these storms may lead to more frequent power failures, requiring airports to invest in additional backup energy supplies.^78^ High winds and lightning strikes also have the potential to damage aircraft and critical airport infrastructure such as power, telecommunications, and navigation systems. This could necessitate more extensive maintenance checks and repairs after extreme storms.^219^ Western New York State airports like Buffalo Niagara International face a growing risk from lake‐effect snowstorms, which are projected to become more common over the next few decades before declining later in this century.^5^ Heavy snowfall and ice buildup could cause flight delays and cancellations and increase the need for snow removal, deicing, and salting of runways. While a changing climate makes severe weather events more likely and potentially more intense, snowfall is projected to decrease over time for the state as a whole.^5^ A 2021 study published in the Journal of Climate suggested that the number of events with moderate or heavy daily snowfall in areas like New York City will decrease.^220^ That study also projected an increase in the frequency of mixed rain and snow and freezing precipitation events in the state.^220^ Heavy precipitation events (whether defined as days with more than 1, 2, or 4 inches of rain) are also projected to occur more often statewide in the decades ahead under the blended greenhouse gas emissions scenario used for the projections developed as part of this assessment.^5^

##### 7.2.1.3 Extreme temperatures

Average annual temperatures in New York State are projected to rise 2.5−4.4°F by the 2030s, 3.8−6.7°F by the 2050s, and 5.1−10.9°F by the 2080s.^5^ Indoor aviation facilities, such as airport terminals and air traffic control towers, may require additional air conditioning capacity to adapt to rising temperatures.^219^ The increased use of air conditioning would in turn increase energy demands and cause strain to the grid and could require additional backup energy capacity. Extreme heat can be particularly dangerous for outdoor airport workers, as the tarmac tends to absorb heat.^221^

Outdoor infrastructure is also vulnerable to extreme temperatures. Asphalt runways, for example, can soften and deteriorate in extreme heat. At the United Kingdom's Luton Airport, soaring temperatures during a heat wave in July 2022 caused a patch repair in the asphalt along the runway to debond and lift.^222^ The airport had to halt flight operations for a few hours while the damage was repaired. The composition and stiffness of the asphalt used in runways is typically based on the local climate; therefore, runways are not necessarily designed to withstand temperatures considered extreme for the area.^222^ Infrastructure damage and reduced runway capacity caused by extreme temperatures can cause flight delays and impact airport revenue.

Because hot air is less dense than cold air, air temperatures have a direct effect on aircraft lift capacity. As temperatures rise, aircraft may generate less lift during takeoff. Beyond a certain temperature threshold, aircraft of a certain weight cannot take off or land on a particular runway length at a particular altitude.^223^ Increases in average air temperatures could require airports to lengthen runways. Some airports (like LaGuardia) that have limited space for expansion may have to resort to increased weight restrictions. A reduction in payload, in the form of both passengers and cargo, could result in millions of dollars in lost revenue for airlines.^223^ For example, high temperatures in Arizona and Seattle recently caused airports to cancel and delay flights.^224^

Along with affecting lift capacity, rising air temperatures have an impact on jet stream winds at higher altitudes. More intense jet stream winds increase flight times for flights heading west, resulting in greater fuel consumption. As a result, some U.S. transnational flights may have to stop to refuel before reaching their destination,^225^ potentially impacting travel times for New York State travelers and cargo.

##### 7.2.1.4 Wildfires

Smoke from wildfires causes air pollution and can reduce visibility. While wildfires are relatively uncommon in New York State, compared with western states, fires do occur every year.^5^ In addition, New York can be affected by wildfires in other states and Canada. In June 2023, smoke from wildfires in Canada blanketed the New York City Metropolitan Area, causing many flights to be delayed.^7^, ^8^ According to the Federal Aviation Administration, air traffic controllers take extra safety precautions during smoky conditions by spacing out flight arrivals and departures. These precautions can cause delays. Additionally, the advanced navigation systems that aircraft rely on during periods of poor visibility do not work as well when there are high concentrations of smoke particles in the air, so pilots must use extra caution during landing and takeoff.^8^

##### 7.2.1.5 Changes in prevailing winds

Studies have shown that a warming climate could lead to changing wind patterns.^226^ A change in prevailing winds could make some runways unusable during dangerous crosswinds, which could reduce airport and aircraft operating efficiency and capacity.

#### 7.2.2 Impacts on the movement of people

People rely on air travel for tourism, recreation, immigration, work, education, visiting family and friends, and various other reasons. Many types of climate hazards can disrupt this travel, as described above. Certain hazards can pose a direct threat to the functioning of aircraft in the air or on the ground, while flights can also be disrupted by hazards that impede other operational aspects of air travel such as the timely transportation of flight crew and airport staff to the airport, ground vehicle operation on the tarmac, intraterminal transportation systems (e.g., shuttles, trains), maintenance operations, and fuel supply. In addition, airport operations can be affected by power outages.

Passenger air travel is a complex operation in which all aspects of the system must function properly to ensure the safety of all passengers. A disruption to one part of the system can lead to cascading impacts, which can be especially acute at the three major airports that serve the global hub of New York City.^227^ For example, during a 3‐day period in mid‐June 2022, a collection of storms caused the three largest U.S. airlines to cancel more than 1600 flights, and more than 6800 flights were delayed.^228^ At LaGuardia, 30% of flights were canceled on the first day, with another 20% canceled the next day.^228^ Delayed and cancelled flights in turn caused air traffic congestion and staffing shortages, leading to more flight cancelations. At the height of disruption from Superstorm Sandy, the cancellation of thousands of flights at JFK, Newark Liberty, and LaGuardia affected about 400,000 passengers worldwide each day, and airlines were losing an estimated $190 million per day in revenue to closures.^229^

The occurrence of clear‐air turbulence is also projected to increase with climate change.^230^ Clear‐air turbulence is characterized by turbulent air currents that occur in cloudless air.^231^ Any increase could add to the risk of injury for passengers and crew.^232^

#### 7.2.3 Impacts on the movement of goods

Air cargo is affected by many of the same impacts that affect air passenger travel. Severe storms, tarmac flooding, or extreme heat can ground passenger and cargo flights alike. The movement of cargo can also be disrupted by climate impacts at intermodal transfer points—locations at airports where trucks and trains offload or pick up cargo, or where cargo is loaded onto aircraft.

Cargo flights may struggle with the physics of operating with heavier loads in extreme climate conditions, as higher temperatures make it harder for aircraft to achieve lift. According to a Columbia University study, by the middle to end of this century, under moderate to high emissions scenarios, approximately 10%−30% of annual flights departing during the hottest parts of the day will have to reduce their weight.^233^ These weight restrictions, which could reach 4%, could lead to flight delays and additional costs to shippers.

### 7.3 Vulnerable populations and systems

#### 7.3.1 Vulnerable populations

Some groups of people are especially vulnerable to the impacts of climate change on air transportation. These populations include airport and airline workers; older passengers and those with disabilities or illnesses; near‐airport communities; and certain travelers with specific dependencies on air transportation.

More than 450,000 New York State residents work in aviation or aviation‐related industries.^211^ Ground crews responsible for loading and unloading baggage, refueling, and guiding aircraft to the gate face health and safety risks during severe weather events and extreme heat days. Section 6.3.1 discusses extreme heat thresholds, heat illness, and associated productivity concerns for outdoor workers in the maritime industry; the same concerns apply to aviation. Ground crews’ heat‐related health risks are exacerbated by working on a heat‐amplifying paved tarmac—and, in some cases, by having to wear heavy protective gear.^234^ Flight crews face risks from increases in clear‐air turbulence. Flight crews and other workers may also face loss of income as more flights are grounded due to extreme weather conditions.^232^

Weather‐related delays that force departing aircraft to wait on runways under extreme heat conditions can cause passenger discomfort and health emergencies and could disproportionately affect older passengers and passengers with disabilities or medical conditions. Addressing the resulting emergencies could result in further flight delays. Disruptions to air transportation also pose a risk to the more than 8000 New York State residents waiting for life‐saving organ transplants.^235^

Near‐airport communities could be affected if climate impacts force the expansion or relocation of runways, facilities, or equipment. Communities affected by airport relocation or expansion not only would have to deal with land‐use issues, but also would face the possibility of increased noise and air pollution. Studies have shown that communities of low socioeconomic status and communities with high concentrations of people of color are disproportionately exposed to noise and air pollution.^236^, ^237^ One study found that exposure to pollutants in the vicinity of airports is as serious as exposure in the vicinity of highways.^238^ Near‐airport communities also supply much of the labor force for airlines, airports, and their supply chains. These workforces include many hourly, low‐income workers who could experience disproportionate economic hardship if climate change impacts disrupt airport operations or reduce demand.

#### 7.3.2 Vulnerable sectors and industries

New York State businesses and customers relying on the shipment of goods via air transportation will be affected by any disruptions to the movement of air cargo. Roughly, 3.4% of cargo and mail freight in the United States is handled through 15 of the state's airports.^211^ Goods that sometimes depend on the speed of air transportation include high‐value goods, physically perishable goods such as foods and pharmaceuticals, and “economically perishable” goods such as holiday items.^239^ Climate change impacts that force the delay, cancellation, or rerouting of air shipments of goods could result in loss of revenue as well as inconvenience to customers. Disruptions in the movement of goods also have a direct effect on freight and distribution companies of other modes, particularly trucking companies and drivers picking up and transporting air cargo to other destinations in the state and beyond the state's borders. New York State serves as a major international gateway for air cargo to and from North America.^239^

The state's tourism and hospitality industries are also vulnerable to climate change impacts on air travel. New York City in particular is a global tourism destination, and its vibrant entertainment and hospitality industries rely on thousands of visitors who arrive daily at the city's international airports. Extreme storms that cause short‐term disruptions to airports are especially harmful to tourism. Increasingly volatile weather and a changing climate could also make travel to vacation destinations more difficult or less desirable and reduce travel demand.

The airline industry itself is particularly susceptible to climate change impacts. Volatile weather patterns and extreme events strain operations and planning, and increased flight delays and cancellations could lead to losses in both jobs and revenue.^232^

#### 7.3.3 Vulnerable regions

The Long Island and New York City assessment regions are most vulnerable to coastal storms and storm surge flooding. As previously mentioned, Superstorm Sandy flooded parts of JFK, LaGuardia, and nearby Newark Liberty in 2012, causing the cancellation of thousands of flights.

Central and northern regions at higher elevations and in closer proximity to the Great Lakes are more vulnerable to lake‐effect storms and other snow and ice storms.^78^ Risk of in‐flight icing—and the need for preflight deicing—could become more acute for airports and flight routes in Western and Central New York. Buffalo Niagara International Airport was disrupted in November 2022 when a severe lake‐effect snowstorm caused a state of emergency and forced several airlines to cancel dozens of flights.^240^, ^241^

### 7.4 Adaptation and resilience

#### 7.4.1 Known methods

The most common methods for adapting to climate change impacts involve changes to infrastructure and equipment. Adaptation measures to protect against sea level rise and flood damage include installing flood barriers, constructing flood‐resistant buildings, installing backflow‐prevention devices, increasing water‐removal capacity, and moving or elevating critical equipment processes.^242^ Superstorm Sandy prompted PANYNJ to develop guidelines to ensure that the agency integrates resilience to sea level rise in capital projects moving forward.^243^ The guidelines mandate that project designs include flood protection against the “100‐year storm” along with a 5‐foot buffer to account for potential future sea level rise and flood levels—a standard that substantially exceeds local and national flood protection standards. The $2.5 billion agency‐wide Sandy Recovery Program includes the reconstruction of a pump station at LaGuardia that powers one of six high‐capacity airfield pumps. The station was rebuilt on 10‐foot stilts to decrease vulnerability to flooding.^243^

Schiphol Airport in the Netherlands has set an example with its efforts to combat sea level rise and flooding. As an airport built on reclaimed land that is below sea level, Schiphol developed a 150‐mile network of drainage structures along with a pumping system to pump out water that collects inside its surrounding levees.^244^ The airport is also implementing other resilience projects to prepare for future climate change, such as harvesting rainwater to use for firefighting and toilet flushing, installing green roofs, and using algae basins for local treatment of polluted runoff.^245^

With the number of days above 90°F projected to increase statewide,^5^ airports and airlines will need to take actions to account for the fact that aircraft lift capacity is reduced at high temperatures. Runways may need to be lengthened at some airports, and airlines may need to limit the load capacity for certain aircraft models.^78^ Another option would be to phase out older aircraft and replace them with newer generations of aircraft whose engines are powerful enough to overcome this issue. To combat heat‐related illness among workers and passengers, airports may need to make operational and infrastructure changes such as creating plans to limit public demand during summer heat waves, installing additional air conditioning capacity,^78^ and increasing shading around key activity zones.

Climate adaptation and resilience measures will require funding. Of New York State's 131 public‐use airports, 87 are eligible for funding through the federal Airport Improvement Program (AIP).^208^ Airports that are not eligible for AIP funding must rely on funds generated from airport revenue or apply for funding from the competitive New York State Aviation Capital Grant Program, run by NYSDOT. The grant program funds airport capital improvements to address critical infrastructure needs and includes energy efficiency and emissions reduction among its criteria.^246^ Additionally, public‐private partnerships among local governments, financial institutions, and private organizations can help ensure risk‐sharing and social protection while mitigating downstream costs.^242^ Downstream costs, which refer to the negative effects on other communities, could affect vulnerable populations living near air transportation centers.

#### 7.4.2 Municipal and regional government concerns

Climate impacts to the air transportation sector could affect land‐use planning, infrastructure design, emergency response planning, and finances for airport owners. Although New York City's large airports are owned and operated by PANYNJ, a bi‐state authority, many other airports in the state are owned and operated by municipal or regional agencies. This includes small airports as well as many with commercial service—for example, Long Island MacArthur Airport (town‐owned and operated); Westchester County Airport (county‐owned); Buffalo Niagara International Airport (owned and operated by a regional transportation authority), and the primary airports that serve Rochester, Syracuse, and Albany (all owned and operated by some combination of city, county, or regional agencies or authorities). Municipal and regional governments can consider climate adaptation strategies when conducting vulnerability and risk assessments and in the planning process for rehabilitating old aviation facilities and building new ones.

#### 7.4.3 Opportunities and cobenefits

Some measures to adapt to climate change can produce multiple benefits. For example, some airports have installed green roofs (i.e., rooftop vegetative covering) on buildings to reduce heat retention, minimize potential heat island effects, reduce stormwater runoff, and serve as insulation.^247^ Another trend is the growing prevalence of solar panel installations on airport buildings or open land. For instance, JFK Parking Lot 9 has 12 megawatts of solar and 7.5 megawatts of battery storage installed, while the roof of LaGuardia's Terminal B Garage has 3500 solar panels.^248^ Although fundamentally a greenhouse gas mitigation strategy, generating clean electricity on site can also boost resilience, particularly as airports in New York State and elsewhere are increasingly switching to electric buses and ground support vehicles,^249^, ^250^ and as increasingly extreme weather poses challenges for the electric power grid.

## 8 MICROMOBILITY

### 8.1 Background

#### 8.1.1 Mode description

The term *micromobility* first came into popular use around 2016.^251^ Due to its recent emergence, the term lacks a universal definition. This assessment uses a definition from the Federal Highway Administration, which describes micromobility as “any small, low‐speed, human‐ or electric‐powered transportation device, including bicycles, scooters, electric‐assist bicycles (e‐bikes), electric scooters (e‐scooters), and other small, lightweight, wheeled conveyances.”^252^ While some of these devices have long been used to get around cities, the past decade has brought new attention to the role micromobility can play in addressing urban transportation problems, such as congestion and greenhouse gas emissions from gas‐powered vehicles. A report by the National Association of City Transportation Officials stated that micromobility can fill gaps for first‐mile/last‐mile trips—that is, trips from a person's home to a mass transit terminal or from their transit stop to their final destination—and can complement mass transit systems by “expanding the number of people who can easily be served by each transit station or giving transit riders options to avoid transfers or overcrowding.”^253^ Micromobility is also an important alternative for neighborhoods underserved by mass transit systems.

*Shared mobility* is a related concept that covers the shared use of cars, bicycles, and other transportation devices. Some forms of shared mobility, such as carpooling, have been practiced for decades, but the category has expanded to include services such as car sharing, ridesourcing, and bike sharing.^254^ The shared use of micromobility devices such as bikes and scooters has expanded rapidly in recent years. As Figure 9‐7 shows, shared micromobility ridership in the United States grew from 321,000 trips in 2010 to 113 million trips in 2022.^255^

**FIGURE 9‐7:**
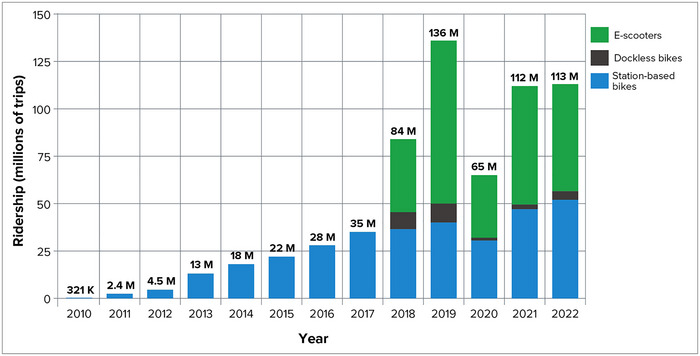
Shared micromobility ridership growth in the United States, 2010–2022. Data from NACTO (2023).^255^

Users of micromobility devices share the roadways with cars, trucks, and buses. They rely on the same infrastructure, including paved roads, bridges, traffic signals and signs, and drainage systems. Micromobility users also have access to a network of shared‐use paths that larger vehicles are excluded from, such as bike paths, bike lanes, walking trails, sidewalks, and curb ramps.

Some shared micromobility systems depend on additional infrastructure. For example, docked systems for bike and e‐bike sharing are made up of numerous docking stations placed at strategic locations around an urban area, such as near mass transit stations. New York City's bike‐share system, Citi Bike, by far the largest in the state, has approximately 1500 docking stations spread across Manhattan, Brooklyn, Queens, the Bronx, and parts of New Jersey.^256^ Docking stations typically include the docks themselves—special racks that lock the bikes—and a kiosk. In most systems, the process of paying for and unlocking a bike is fully digitized. The rider checks out a bike using a mobile phone app or a computerized interface at a kiosk. In this way, bike sharing relies on cloud computing infrastructure and local internet service to enable individual devices to share data with a centralized server.

Like many parts of the country, New York State has experienced a surge in biking and other forms of micromobility. The increase in ridership became the topic of headline news during the early stages of the COVID‐19 pandemic, when millions of people in urban areas sought alternatives to public transportation. However, the groundwork for the micromobility boom was laid years before the pandemic as many cities in the state responded to a renewed interest in biking and other forms of active transportation by investing in plans to better accommodate bikes on city streets. Rochester, for example, developed a master plan for improving biking infrastructure in 2011.^257^ Buffalo released a similar plan in 2016.^258^ The League of American Bicyclists now recognizes both cities (along with New York City and Ithaca) as Bicycle Friendly Communities.^259^

In New York City, the number of people who use a bike as their primary mode of commuting to work doubled over the 10‐year period prior to the pandemic, from 25,000 in 2010 to more than 52,000 in 2019.^260^ The city's biking infrastructure expanded over the same period and has continued to grow. Since 2017, the city has added more than 20 miles of new protected bike lanes each year.^261^ As of 2022, the city had a total of 1525 miles of bike lanes, of which 644 were protected.^260^

Shared micromobility offerings have also expanded across the state. Besides Citi Bike in New York City, which maintains a fleet that has grown to about 25,000 bikes,^256^ smaller shared mobility programs have also operated in cities such as Albany, Buffalo, New Rochelle, Rochester, and Syracuse.^262^ These programs have offered bike‐sharing only (pedal bikes and/or e‐bikes) or a mix of bike sharing and e‐scooter sharing. In addition, several e‐scooter‐sharing companies have offered service in the Bronx. While some of the state's shared mobility programs are well established, others are not yet a permanent component of local transportation systems. In recent years, programs have shut down in places such as White Plains and Long Beach, even as new programs have launched in other cities. Ithaca began a new e‐bike‐sharing program in 2022, filling a gap left when a previous program was discontinued in 2020.^263^

While micromobility is mainly used for moving people, it is also used to move goods. With the growth in app‐based food delivery services, delivery by bicycle has become more common in many cities in the state. New York City, which has a long tradition of bike delivery, now has approximately 65,000 workers who deliver food and other goods by bike, often using throttle‐powered or pedal‐assist e‐bikes for added speed.^264^ In 2019, New York City launched a pilot program to use cargo bikes for parcel deliveries, partly as a way of relieving street congestion and greenhouse gas emissions caused by idling freight vans and box trucks. As of January 2021, six freight and e‐commerce companies were operating more than 350 cargo bikes in the city, making as many as 45,000 deliveries per month.^265^ The pilot is based on a model introduced in Europe, where the use of cargo bikes has become common in some cities.

#### 8.1.2 Scope

Micromobility, as discussed in this section, encompasses all forms (motorized and nonmotorized) of bikes, scooters, and skateboards along with devices such as rollerblades and roller skates, e‐skates, and hoverboards and other self‐balancing boards. The devices used in micromobility can be individually owned or shared. Examples of shared micromobility include docked and dockless systems for bike and e‐bike sharing, and dockless systems for e‐scooter sharing.

Manual wheelchairs, electric wheelchairs, and other similar devices for individuals with mobility impairments are generally not considered micromobility devices, because they have their own legal definition and are regulated as medical devices.^266^ In addition, regulations under the Americans with Disabilities Act give people with disabilities the right to operate wheelchairs and other mobility devices in settings such as office buildings, restaurants, and other businesses where the use of micromobility devices by nondisabled people would be prohibited.^267^ While characterizing the use of wheelchairs and other similar devices by people with disabilities is outside the scope of this section, the discussion that follows does, in places, touch on specific climate‐related vulnerabilities that these medical devices share with micromobility devices.

#### 8.1.3 Connections

Micromobility intersects with mass transit services, including buses, subway, commuter rail, trolleys, and ferries. Bikes, scooters, and other devices can cover the “first and last mile” of transit trips or provide an alternative to mass transit and private cars for short trips. Micromobility also can serve as a substitute for mass transit in underserved communities (sometimes call “transit deserts”) and during disruptions in public transportation, including those caused by extreme storms. Studies have found that bike‐sharing systems see a considerable increase in ridership during transit system shutdowns.^268^

With the recent surge in shared micromobility ridership, several researchers have examined whether micromobility complements public transportation systems or competes with them. One literature review found that bike sharing can contribute to an increase in public transportation ridership by providing users with an option for first‐mile/last‐mile transportation.^269^ However, other studies cited by the authors found evidence that bike sharing may draw riders away from public transportation by offering an alternative that is faster and less expensive. The timing of the studies, conducted during the COVID‐19 pandemic, complicates the findings, as some people might have chosen micromobility over public transit to reduce exposure.

Micromobility also has connections to other sectors evaluated in this assessment, including the energy sector. Battery‐powered micromobility devices such as e‐bikes, e‐scooters, and hoverboards depend on electricity for charging, yet studies show that micromobility can reduce overall energy consumption from passenger travel.^270^ From a human health perspective, active transportation methods like biking have health benefits, and shared micromobility can also help improve access to health care facilities. However, the use of micromobility devices in crowded urban environments carries risk, with tens of thousands of injuries occurring every year nationwide.^271^

### 8.2 Observed and projected impacts

#### 8.2.1 Impacts on the physical system

Data on observed impacts of climate change on micromobility are limited, as this mode is evolving rapidly and several types of micromobility have only gained widespread use in the last few years or decades. However, some connections to climate impacts can reasonably be inferred based on other evidence.

As noted above, users of micromobility devices share the roadways with cars, trucks, and buses, and rely on the same infrastructure. Section 3.2 describes climate hazards that pose a threat to roadways and other related infrastructure, such as bridges, electrical systems, and drainage systems. Any climate hazard that damages roadways or makes them impassable (e.g., due to flooding, fallen trees, or downed power lines) will have an impact on micromobility use. Such hazards include extreme heat, drought, intense precipitation, severe storms, and coastal inundation related to sea level rise.

Micromobility systems also face risks to the extent that infrastructure elements such as charging stations (often located outdoors), telematics, and mobile apps rely on electric power and telecommunications networks. Electric power and telecommunications are increasingly vulnerable to outages due to projected increases in severe weather, flooding, and other climate impacts.^272^ Power outages from extreme weather events can disable docking stations and also interrupt electronic payment services by bringing down cloud computing systems, preventing users from using bike sharing.^273^

#### 8.2.2 Impacts on the movement of people

Climate change will affect micromobility use to the degree that it increases the frequency or intensity of weather conditions that either encourage or deter ridership. Intuitively, micromobility is more sensitive to weather and road conditions than other transportation modes. Devices such as bikes, scooters, and skateboards lack protective shells and climate control, so the rider is exposed to the elements and easily affected by changing weather conditions. Broadly speaking, nice weather encourages the use of micromobility, while precipitation, extreme high and low temperatures, and stormy conditions reduce the use of micromobility.

Because of New York State's climate, with its cold, snowy winters and hot, humid summers, micromobility use varies seasonally. Figure 9‐8 shows monthly variations in the average number of trips per day for New York City's Citi Bike program. Some other bike‐share and scooter‐share programs in the state, such as those in Albany, Rochester, and Buffalo, shut down completely during winter.^274^, ^275^, ^276^

**FIGURE 9‐8:**
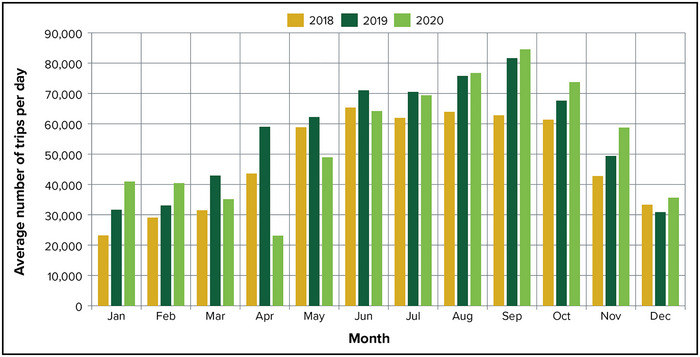
Average Citi Bike trips per day, by month, 2018–2020. Figure adapted from New York City Department of Transportation (2021).^277^

Several studies have examined how different weather conditions influence micromobility ridership. One study on the impacts of temperature on bike‐share usage in New York City concluded that ridership increases as temperatures rise, up to a threshold of about 79−82°F (26−28°C); above that, ridership begins to decline.^278^ Other studies have reported similar patterns, though the ideal temperature range for biking appears to vary from city to city, based on the local climate.^279^, ^280^

Precipitation, strong wind, and high humidity have also been shown to deter the use of micromobility, with snow having the most pronounced effect. However, evidence suggests that the magnitude of the impact is smaller on weekdays, when micromobility use is more likely to be work‐related and trips are less likely to be canceled due to inclement weather.^279^, ^280^

BOX 4 **Climate change impacts on wheelchair use**

Like micromobility devices, wheelchairs and other wheeled mobility devices are highly sensitive to certain types of weather and sidewalk conditions when used outdoors. For example, when not properly cleared, snow can create a barrier to wheelchair use. Wheelchairs can become stuck in deep snow or in frozen ruts.^281^ Ice can cause wheels to lose traction and put the operator in danger of sliding out of control. Heavy rain can create puddles that are impassible, and extreme storms can result in conditions that place wheelchair users at risk, including flooding, reduced visibility, and debris‐strewn sidewalks and streets.^282^ Changes in climate conditions in New York State will reduce some risks for wheelchair users while increasing other risks. Snowfall, for example, is projected to decrease overall.^5^ However, more winter precipitation is expected to fall as rain, creating different kinds of hazards. Heavy rainstorms and some kinds of severe storms are expected to become more frequent and/or intense.^5^

##### 8.2.2.1 Observed impacts

Across the state, the average annual temperature has warmed at a rate of approximately 0.21°F per decade, and annual precipitation has increased at a rate of 0.47 inches per decade.^5^ While the state's climate is incrementally warmer and wetter than it used to be, there is no evidence that these gradual changes have had a direct impact on trends in the use of bikes and other micromobility devices. Micromobility's recent surge in popularity has more to do with factors like advances in the technology of micro‐vehicles, widespread application of the shared mobility model, changing attitudes about automobile ownership, and investments in micromobility infrastructure like bike lanes and bike paths.^283^ Some users may choose micromobility out of a desire to reduce their carbon footprint and slow global warming, but there have been no widespread claims that the warming climate has produced conditions that are better suited for micromobility.

In some instances, micromobility ridership has experienced sudden short‐term increases due to extreme weather. When flooding from Hurricane Ida disabled New York City's subway system in early September 2021, the Citi Bike bike‐share system had the busiest day in the program's history to date, with more than 126,000 trips recorded in a single 24‐h period.^284^ Cases like this illustrate how micromobility can contribute to the resilience of transport systems by acting as an alternative mode during service disruptions. However, micromobility's vulnerability to the same conditions that cause mass transit disruptions (flooding, power outages) raises questions about its ability to serve as a dependable alternative.

##### 8.2.2.2 Projected impacts

Researchers have begun to look at the question of how future climate change is expected to impact micromobility. One study that modeled bike‐share ridership patterns using data from the Citi Bike system predicted that projected average temperature increases over the period 2040−2069 would result in a net increase in bike usage of up to 3.1%.^278^ Although summer ridership is expected to decline as temperatures more frequently exceed the 79−82°F threshold, the authors projected a large increase in winter ridership and smaller increases in spring and fall ridership.^278^ Another study modeled how bike‐share ridership in 40 cities would change in reaction to small increases in the Universal Thermal Climate Index, an index that combines four weather variables (air temperature, humidity, solar radiation, and wind) and was developed as an indicator of thermal comfort for outdoor activities. The authors concluded that a 1.8−3.6°F (1−2°C) increase in the index would lead to increased bike‐share ridership in cities in cold climates and decreased ridership in cities in warm climates, but that the effects would be small.^280^

While climate change is measurable as a creeping change in average conditions, it also manifests itself in extreme weather events such as hurricanes, heat waves, and coastal flooding. The impacts of these events on micromobility are unpredictable, and therefore, difficult to model. Shared micromobility services are sometimes suspended during severe weather, as happened in 2021−2022 when an e‐scooter‐sharing pilot in the Bronx was temporarily shut down during Hurricane Henri and two major snowstorms.^285^ A power outage caused by severe weather can bring down a cloud computing system, resulting in a service disruption in a bike‐sharing program. Conversely, as described above, severe weather can sometimes lead to a temporary increase in micromobility ridership.

#### 8.2.3 Impacts on the movement of goods

The same climate hazards (e.g., extreme heat, flooding, severe weather) that can lead to short‐term or seasonal declines in general micromobility use can also create challenges for delivery workers who depend on bikes or scooters. However, as described in Section 8.3.1, workers making deliveries for app‐based services have a financial incentive to continue working even in inclement or hazardous conditions. More study is needed to assess how changing climate conditions could affect these workers and the movement of goods via micromobility devices.

### 8.3 Vulnerable populations and systems

The impacts of climate change on micromobility will affect different populations in different ways. This is partly because the demographics of micromobility use vary from city to city and neighborhood to neighborhood, and partly because different people use micromobility in different ways, from recreational outings to daily commutes to deliveries.

#### 8.3.1 Vulnerable populations

Studies of shared micromobility programs have shown that the most frequent users tend to be males between the ages of 25 and 45, with above‐average incomes and levels of education.^286^ White populations tend to be overrepresented in shared micromobility ridership, while people of color are underrepresented.^287^ A 2019 report by the Urban Politics and Governance research group at McGill University found that New York City's Citi Bike system disproportionately serves high‐income New Yorkers who already have subway access, and that the Citi Bike service area is “twice as white as the rest of the city.”^288^ The report stated that of 1.2 million city residents who gained access to Citi Bike between 2013 and 2019, “only 48,700 of them are underprivileged people lacking subway access.”

Nationwide, many shared micromobility programs have explored ways to increase ridership in frontline communities and provide equitable access.^253^ Several programs in New York State, including those in Rochester, Syracuse, and New Rochelle, offer discounted fares for qualified residents. Citi Bike offers $5 monthly memberships to residents of public housing operated by the New York City Housing Authority, as well as to recipients of aid from the federal Supplemental Nutrition Assistance Program. In 2019, Citi Bike formed an Equity Advisory Board to oversee the expansion of the system into low‐income neighborhoods.^289^

To the degree that micromobility use is impacted by future changes in New York State's climate, people who depend on micromobility devices for transport to jobs or for access to basic services will be among the most affected. This population includes people who live in neighborhoods with limited access to mass transit and people who are unable to afford more expensive transit options. Data from the American Community Survey show that people in the lowest income brackets are the most likely to depend on bikes (all bikes, not just bike‐share programs) for commuting.^290^

Another vulnerable population is people who depend on micromobility for the actual execution of their job responsibilities, including tens of thousands of delivery workers who rely on bikes, e‐bikes, and various forms of e‐scooters. These people work outdoors and are expected to deliver in all weather conditions, including extreme heat and cold and heavy precipitation. During Hurricane Ida, when New York City's government urged residents to stay home and take shelter, many bike delivery workers continued to make deliveries through flooded streets and heavy downpours. The *New York Times* reported afterward that “food delivery companies offer extra money when demand is high or during inclement weather, leading some couriers to risk their safety for the promise of potentially higher wages.” The *Times* also reported that workers “fear retaliation from the companies if they chose to turn down deliveries”^291^—a situation exacerbated by the undocumented status of many of these workers.^264^

While not considered micromobility users, people with mobility impairments can also find themselves in situations of extreme vulnerability at times when the use of wheeled mobility devices becomes hazardous or impossible. Residents of high‐rise buildings can become stranded in their apartments during power outages, unable to leave via stairwells.^292^ Loss of power can also make it impossible to charge electric wheelchairs. If evacuation is necessary, mobility‐impaired people may not be able to transport themselves to public transit terminals in weather conditions that make sidewalks and streets impassable. They are likely to need special support, such as pickup via a vehicle that has a wheelchair lift.

#### 8.3.2 Vulnerable sectors and industries

Micromobility is still emerging, but some have projected that the micromobility industry will become a $300 billion global market by 2030 and will have a substantial impact on public and private transportation ridership.^293^ Given that changing climatic conditions are projected to increase micromobility ridership in some seasons and decrease ridership in others, it is hard to predict what effect climate change will have on the expected growth of the industry.

As discussed above, climate change is expected to multiply the challenges and hazards faced by food delivery workers who rely on bikes and other micromobility devices. It could also affect initiatives to increase the use of cargo bikes for parcel deliveries in New York City.

#### 8.3.3 Vulnerable regions

Micromobility is usually thought about as a means of transportation for urban areas because trip distances are generally short. The average trip for a scooter or bike‐share user in the United States is about 1−1.5 miles and lasts about 11−12 min.^253^ In rural areas, micromobility is more often used as a means of recreation or healthy activity. This is partly because average trip distances are much longer in rural areas, making it difficult for residents to commute or access basic services using a bike or other micromobility device, and partly because of a lack of infrastructure. A 2020 report by the National Center for Mobility Management stated that “many small and rural communities are located on state and county roadways that were built to design standards that favor high‐speed motorized traffic, resulting in a system that makes walking and cycling less safe and uncomfortable.”^294^

Recreational biking has increased in popularity in many areas of New York State. The Society and Economy chapter includes a discussion of climate impacts on recreational biking.

### 8.4 Adaptation and resilience

#### 8.4.1 Known methods

Micromobility itself can add resilience to the transportation system. Cheng et al. define transport resilience as “the ability of the transport network to withstand the impact of extreme weather, to operate in the face of such events and to recover promptly from its effects.”^268^ That study goes on to say that one way to achieve resilience is by introducing redundancies. Whether micromobility complements or competes with mass transit, redundancy between the two modes contributes to resilience. Micromobility not only has the potential to help reduce congestion and greenhouse gas emissions from fossil fuel−powered vehicles; it can also serve as an adaptation that helps residents get around when climate‐related disruptions affect other modes.

The devices used for micromobility—bikes, scooters, skateboards, and so on—are not easily modified to protect the rider from severe weather and moderate the impacts of climate change. They are, by design, lightweight, and small enough to store in an apartment or wheel onto an elevator. They lack a protective shell and are relatively inexpensive. Some manufacturers are experimenting with products such as three‐wheeled micro‐vehicles with shells to protect the rider from precipitation. Courier companies have begun using partially or fully enclosed e‐bikes for parcel deliveries in some cities. In a 2019 report, SAE International wrote that “industry experts have clearly indicated that we are only scratching the surface with what is possible in terms of microvehicle shape, size, and capability.”^283^ Yet, even as new vehicle types emerge, they are unlikely to replace the bike, the scooter, and the skateboard—simple, affordable, convenient devices whose designs have withstood the test of time and proven their worth in hundreds of cities around the globe.

Some cities have begun looking at another approach to addressing the vulnerability of micromobility to extreme weather conditions: building state‐of‐the‐art infrastructure such as covered bike paths or paths that are heated to keep them free of snow and ice. In South Korea, a 3‐mile section of bike path connecting the cities of Daejeon and Sejong is covered with a roof of solar panels that protect riders from sunlight and precipitation while also generating clean energy.^295^ An et al. report that “Qatar has proposed to build a shaded, solar‐powered, mist‐cooled bicycle path of 30 kilometres.”^279^ On a more basic level, cities can increase the viability of micromobility by building and maintaining resilient stormwater management systems to prevent street flooding from extreme precipitation.

#### 8.4.2 Municipal and regional government concerns

As micromobility usage expands, municipal and regional governments are addressing concerns about climate impacts, usage, and safety. Local governments have the responsibility to address disparities in micromobility availability and usage among varying populations and to protect the health and safety of users of micromobility devices. Large cities such as Chicago, Los Angeles, and Berkeley, California, have increased the number of bike lanes, restricted the riding and parking of micromobility devices on public sidewalks, and strategically placed docking and charging stations in underrepresented communities to meet micromobility goals.^296^ Local governments have also advocated for micromobility use as a part of broader transportation and climate action objectives.

#### 8.4.3 Opportunities and cobenefits

The boom in micromobility ridership brings several benefits. Biking and other active transportation methods have well‐documented health benefits. Micromobility also provides an important alternative for neighborhoods underserved by mass transit and can help individuals access health care and employment opportunities. By filling gaps for first‐mile/last‐mile trips, micromobility can expand the reach of mass transit systems by increasing the number of people who can access transit stations. In 2021, 37% of North American micromobility trips replaced car trips, reducing traffic congestion on roadways and offsetting approximately 54 million pounds of carbon dioxide emissions.^296^

## 9 LOOKING AHEAD

This section looks at opportunities for positive change that can grow out of climate adaptation efforts and identifies emerging topics and research needs in the Transportation sector. The section concludes by summarizing the major findings and recommendations presented in the chapter.

### 9.1 Opportunities for positive change

The urgent need for action on climate change adaptation could catalyze positive change, particularly if adaptation goes hand in hand with greenhouse gas emission reduction strategies that ultimately help to reduce the severity of future climate impacts. Given that transportation is the second largest source of greenhouse gas emissions in New York State,^297^ it is especially important that adaptation solutions not increase emissions. Conversely, it is also important that adaptation solutions address the climate risks inherent in certain greenhouse gas mitigation solutions, like electrification and the increased reliance it puts on the electric power grid. The interconnectedness of transportation systems presents an opportunity to address multiple historical legacies simultaneously while making progress toward a more sustainable and equitable built environment.

The paragraphs below consider some of the opportunities associated with actions to reduce vulnerability and improve resilience to climate impacts.

#### 9.1.1 Aspiring to resilient and restorative transportation investments

Historically, the development of some transportation infrastructure (such as highways) has had the effect of separating and sometimes isolating communities. Addressing the consequences of past choices can deliver sustainable transportation solutions that connect people and move them where they need to go. As New York State looks to adapt transportation systems and infrastructure to address climate risks, there is an opportunity to consider who experiences benefits and adverse effects from transportation systems and how these systems are distributed. Large‐scale capital projects, such as placing caps on highways or developing public greenways, can support this aim. Another restorative approach to transportation adaptation involves addressing procedural inequities in transportation planning by changing the purpose of and approach to public engagement.^24^ Public engagement can transition from a compliance requirement to an opportunity to incorporate diverse perspectives into decision‐making and to coproduce innovative solutions.

#### 9.1.2 Adapting to climate change without increasing contributions to climate change

Regularly scheduled asset maintenance and investments to adapt transportation systems to climate impacts will require construction. Planners can consider the life cycle impact of construction and seek opportunities to reduce environmental impacts. Design standards can integrate climate change considerations, and project designs can incorporate risk‐informed decision‐making. Integrating environmental declarations and life cycle analysis into construction planning is one approach to reducing the climate impacts from construction. Life cycle analysis can also consider future maintenance and repair costs, which would incentivize more resilient design.

#### 9.1.3 Meeting multiple needs through redesign

- **Creating designs that improve adaptation, resilience, and accessibility**. Planners and engineers can design transportation infrastructure to incorporate adaptive elements and respond to new challenges, such as increases in the frequency or intensity of some types of severe storms, flooding, and extreme heat. The need to revisit designs can serve as an opportunity to update existing infrastructure to incorporate not just resilience measures, but also other improvements that increase accessibility, reduce environmental impacts, and consider local needs. For example, when redesigning an intersection to reduce disruptions from flooding, planners could opt to include new flood‐control measures (e.g., rain gardens) as well as wider sidewalks and improved road markings. As a result, the intersection would experience improvements in safety, stormwater management, pollution from runoff, and accessibility.
- **Improving access to safe, low‐carbon mobility**. Actions to make roadway infrastructure more resilient (e.g., by installing permeable paving materials) present an opportunity to improve safety conditions through better sidewalks, bike lanes, signals, and road markings. These changes could have the synergistic benefit of enabling micromobility and active mobility, which in turn could help address problems with traffic congestion and local air quality. Improving the quality and safety of road infrastructure to accommodate multiple modes of transportation also contributes to resilience through redundancy, enabling multiple modes to travel from one point to another.
- **Considering natural systems and transportation systems**. Planners can consider the natural environment and ecosystem services as tools to support adaptation and resilience through innovative design and conservation. When managed effectively, natural systems near transportation assets can provide protection from natural hazards. For example, healthy seagrass and oyster colonies can reduce wave action that affects coastal transportation assets,^298^, ^299^ while effectively managed roadside environments inland can reduce erosion and manage flooding.
- **Considering resilience and adaptation in a future with increased automation and connected and automated vehicles**. Investments in transportation adaptation and resilience will ideally consider structural changes in transportation technologies. The increase in connected and autonomous vehicles creates new pressures and needs for transportation systems.^300^

### 9.2 Emerging topics and research needs

Many issues emerged during the development of the chapter that could benefit from further investigation. Transportation resilience and adaptation to climate change is an active area of research and implementation. The following are examples of possible research and development priorities:

- Improving methods and tools for risk‐ and equity‐informed decision‐making to support capital prioritization. Such tools could support decision‐makers in prioritizing and communicating the returns on investments and social impacts associated with investments.
- Investigating the impacts of emerging technologies such as electrification and connected and autonomous vehicles on adaptation and resilience, electric systems, the built environment, and society.
- Improving methodologies for life cycle assessment of transportation infrastructure and data on embodied carbon to inform opportunities for construction.
- Improving the availability of data on the condition of transportation infrastructure and the effectiveness of existing adaptation measures to support improved planning and design for future climate conditions.
- Identifying opportunities to improve engagement and participation to transition in a way that integrates host communities as stakeholders and codesigners with shared ownership.
- Investigating managed retreat, the movement of assets away from risk, as one of multiple strategies for reducing the impact of flood hazards on transportation infrastructure in low‐lying coastal areas.
- Advancing approaches to modeling and planning for multimodal trips and goods movement. Additional data and research could improve understanding of patterns of multimodal mobility and how to maximize resilience to climate change.
- Improving understanding of how transportation infrastructure and activity produce benefits and negative impacts in surrounding communities, and also improving understanding of the ridership needs of historically underrepresented and underserved communities.
- Improving understanding of interdependencies, redundancies, and opportunities for coordination and collaboration to improve system‐wide resilience.

Attention to issues such as these will be critical as New York State develops transportation system resilience strategies and plans to both adapt and thrive under a changing climate.

### 9.3 Conclusions

Climate change is already affecting transportation systems in New York State. Key hazards—including extreme temperatures, heavy precipitation and flooding, severe storms, and sea level rise—are causing damage to transportation infrastructure, disrupting operations, creating safety risks for travelers, and straining businesses in ways that ripple through the economy. In recent decades, the state has experienced challenges from notable events that represent a wide range of climate impacts. In October 2012, for example, Superstorm Sandy flooded parts of the New York City subway system, shut down the three major airports in the metropolitan area, and disrupted the movement of cargo through maritime shipping terminals for nearly a week. In November 2022, a severe lake‐effect snowstorm produced a state of emergency in Western New York, shutting down roadways and causing airlines to cancel flights. In June 2023, smoke from hundreds of wildfires in Canada blanketed the New York City Metropolitan Area, causing many flights to be delayed. Impacts like these are expected to become more common in the future, with evidence suggesting that inland and coastal flooding pose the highest risk.

New York State's transportation systems are multimodal and interconnected. This means that a climate‐driven disruption in one mode can cascade to create impacts in other modes, but also that there are often backup modes available—a source of redundancy and resilience. Climate impacts on transportation can also affect other sectors, while climate impacts on other sectors can affect transportation systems, such as when a severe storm causes a power outage that disables subway service.

Aging infrastructure is a nonclimate stressor that causes transportation agencies and departments to invest heavily in maintenance, retrofitting, or replacement. Changing climate conditions can exacerbate problems with aging infrastructure components, speeding deterioration and shortening expected lifespans. Replacing infrastructure earlier than planned increases costs to society. Transportation systems also face the need to adapt to new dynamics and technologies that affect how people and goods move through the region. Examples of changes include the evolution of micromobility, electrification of personal vehicles and fleets that increases dependence on the electric power grid, just‐in‐time delivery, increases in the size of container ships, and the integration of autonomous and connected vehicle technologies. Public transit agencies are also experiencing lagging impacts from the COVID‐19 pandemic on ridership patterns and revenue sources.

Individual modes of transportation confront some shared climate risks and some unique risks. New York State's extensive network of roadways, bridges, and tunnels is exposed to multiple climate impacts, from coastal and riverine flooding to lake‐effect snowstorms and extreme heat. Micromobility and rail are similarly vulnerable, with micromobility users facing additional risk because they are exposed to the elements. Mass transit is comparatively more concentrated in urban geographies where small disruptions can have amplified effects because of the number of riders served. Mass transit is especially impacted by flooding and extreme temperatures. The proximity of New York State's largest air transportation hubs to coastal areas presents a unique challenge for aviation. Other challenges to aviation include the growing impacts of wildfire smoke and disruptions from winter storms. Maritime ports and vessels are vulnerable to extreme storms (such as tropical cyclones and hurricanes), coastal flooding, sea level rise, changes in precipitation patterns, extreme wind, and changes in wave conditions. Extreme heat poses a threat to outdoor workers throughout the transportation sector.

Climate change compounds existing challenges in transportation such as legacy infrastructure and inequities in the way benefits and disbenefits from mobility are distributed among society. Mobility inequities can be manifested in different ways. As public funds are invested in making transportation infrastructure more resilient to climate change, attention can be given to who benefits and who experiences adverse effects. Transportation agencies and private employers will also need to take steps to maintain safe work environments for the transportation workforce in the context of extreme temperatures, air quality concerns, and other climate hazards. Ultimately, adapting to future conditions provides an opportunity to reconsider and re‐envision the movement of people and goods, prioritizing accessibility, sustainability, efficiency and reliability, and adaptability when designing the future of New York State's transportation systems.

## 10 TRACEABLE ACCOUNTS

Traceable accounts examine each key finding in depth. They provide citations that support each assertion and present the authors’ assessment of confidence in each finding.

### 10.1 Key Finding 1

**Many climate hazards could damage or disrupt New York State's transportation systems, with heavy precipitation and sea level rise presenting the most severe risks**. All modes of transportation are vulnerable to increased inland flooding driven by more intense storms or increased coastal flooding driven by sea level rise and storm surge. These events can block vital routes, impede the safe operation of vehicles, damage infrastructure, and accelerate long‐term infrastructure deterioration. Transportation agencies have begun to respond to this threat with a combination of “gray” infrastructure, like elevating structures and creating barriers to keep floodwaters out of tunnels, and “green” infrastructure that uses natural solutions to absorb or drain excess water.

#### 10.1.1 Description of evidence

There is extensive documentation of extreme weather events in recent years impacting New York State and its transportation systems with hydrological hazards, including flooding from severe tropical and extratropical storms^26^, ^33^, ^301^ as well as extreme precipitation,^302^ severe snow storms,^303^ and coastal storm surge that is made more dangerous with rising sea levels.^89^, ^304^ Documentation and scientific assessment of these events provide evidence of impacts to each mode of transportation, including roads and highways,^38^ mass transit,^84^ railway transportation,^129^ maritime transportation,^165^ air transportation,^305^ and micromobility.^279^, ^280^ In addition to the impacts on transportation infrastructure, various studies describe evidence for the impacts of hydrological climate hazards on the ability of vehicles to move across land, sea, and air.^46^, ^167^, ^216^ This assessment's updated physical climate projections provide evidence of increasing risk of severe storms, extreme precipitation, and sea level rise.^5^ Other climate hazards with a range of documented impacts on transportation are also intensifying,^5^, ^25^ but the preponderance of evidence reviewed in this chapter points to hydrological hazards as the most impactful, particularly in New York State. This chapter profiles several of the most disruptive and costly climate‐related disasters in the state's recent history, and nearly all involved inland or coastal flooding, as epitomized by the most damaging event to date: Superstorm Sandy. Evidence of adaptive actions already taken or underway within New York State include proactive planning by NYSDOT,^69^ elevation of critical infrastructure at LaGuardia Airport,^243^ and investments by the MTA and PANYNJ in fortifying subway infrastructure.^106^, ^111^, ^201^ The state has made funding available through the Extreme Winter Recovery program for municipalities to maintain and repair roads and bridges affected by climate‐related events.^29^ Green infrastructure has been implemented as well—for example, the stormwater collection system and bioretention basins constructed in Cayuga County and profiled in BOX 3.

#### 10.1.2 New information and remaining uncertainties

There is much research on the impacts of severe storms, sea level rise, and heavy precipitation on long‐existing transportation systems, but researchers are less certain about the performance of new and planned transportation systems under projected climate conditions.

While sea level rise is a certain risk in New York State, less is understood about the risks posed by potential changes in the surface levels of Lakes Erie and Ontario. This uncertainty has implications for maritime traffic as well as near‐shore road and railway infrastructure. Chapter 2, New York State's Changing Climate, explores this uncertainty in more depth.

#### 10.1.3 Assessment of confidence

There is **very high** confidence that hydrological forces in the form of increased heavy precipitation and sea level rise will present an increasing risk to New York State's transportation systems. There is a wide body of documented events that have impacted these transportation systems, as well as published literature assessing these impacts and projecting future climate impacts based on existing and planned transportation infrastructure and patterns of use. These sources are consistent in their assessment of climate change−driven hydrological risks to New York transportation infrastructure. Additionally, Chapter 2 of this assessment, New York State's Changing Climate, documents **very high** confidence that the climate will continue to change throughout the remainder of the 21st century, even if the world achieves some success in reducing the rate of greenhouse gas emissions. These changes will bring higher sea levels, more intense storms, and increasingly heavy rain events to New York State.^5^ There is **very high** confidence that transportation agencies and departments in New York State are already using gray and green infrastructure to better manage and protect against riverine, urban, and coastal flooding—a finding based on many documented projects.

### 10.2 Key Finding 2

**New York State's transportation systems are vulnerable to impacts that cascade across modes of transport and across sectors**. The intermodal and cross‐sectoral nature of the state's transportation network increases the potential for cascading impacts, which can affect physical infrastructure, user accessibility, and supply chains. For example, power outages or floods that disrupt mass transit or intermodal hubs can create a surge of demand in other modes, increasing congestion and delays. Solutions include not only making individual modes more resilient, but also ensuring the availability of reliable backup modes to keep New York moving.

#### 10.2.1 Description of evidence

A large body of studies have documented the broad‐ranging impacts that weather can have on all modes of transportation, including roads and highways,^25^, ^46^, ^306^ mass transit systems,^82^, ^84^, ^85^ heavy railway,^95^, ^129^, ^135^ maritime transportation,^165^, ^167^, ^170^, ^172^, ^177^ air transportation,^8^, ^78^, ^222^, ^226^, ^230^, ^305^ and micromobility.^278^, ^280^, ^282^

New York's transportation systems are complex; the state's roadway system is one of the oldest in the nation and one of the most expensive to maintain.^29^, ^307^ It is also overseen by multiple levels of government. Outdated infrastructure can be difficult to replace and can interface with new construction. However, the state's roadway system is crucial for connecting residents and businesses to the state's marine ports of entry, airports, railways, and mass transit. Buses and micromobility modes directly rely on road infrastructure, though they are considered separate transportation modes. The connectedness of New York State's systems presents an increased likelihood of climate‐driven cascading impacts from one mode to another.^29^, ^32^, ^168^, ^172^, ^296^, ^308^ For example, the relatively large share of people in New York who commute to work using a method other than driving alone means the potential for more interdependent mode shifts—such as more people in the New York City area depending on multiple modes (like a combination of bus, commuter rail, subway, and/or ferry, plus driving, walking, or micromobility for the “first and last mile”) to get to and from work.

Climate‐driven disruptions to transportation also affect the economy overall,^165^, ^182^ agriculture, and human health and safety.^26^, ^29^, ^55^, ^301^ Similarly, climate‐driven disruptions to other sectors, primarily the energy sector, can affect transportation systems.^43^, ^78^, ^250^, ^270^

#### 10.2.2 New information and remaining uncertainties

Technology advancements in transportation—such as EVs, connected and autonomous vehicles, and transportation network companies—are rapidly evolving. Their impact on travel patterns and their requirements from New York's transportation infrastructure remains to be seen. These new technologies may have specific climate vulnerabilities that are not yet studied.

While the evidence points to risks increasing throughout the remainder of the 21st century, exact risks are difficult to quantify, particularly with an increasingly variable or unstable climate and the fact that much of the damage may be caused by a few individual extreme events, which climate models can only project in terms of general likelihood of occurrence. Furthermore, the impacts of climate change can accumulate and intensify over time by causing incremental damage to transportation infrastructure, making it more susceptible to, for example, the next storm or heat wave. The extent of these compounding effects is uncertain.

#### 10.2.3 Assessment of confidence

There is **very high** confidence that individual transportation modes in New York State are vulnerable to the physical climate hazards described throughout this assessment, as shown by numerous assessments, studies, and real‐world examples. The body of evidence supports an assessment of **high** confidence in the finding that the interconnectedness of New York State's transportation systems can lead to climate impacts that cascade across modes, based on empirical observations and known dependencies.

### 10.3 Key Finding 3

**Climate change could exacerbate existing transportation system inequities, which well‐planned climate solutions can help to reduce**. The construction of some transportation infrastructure such as highways or transit lines has created disproportionate burdens, such as highways that severed historically Black neighborhoods decades ago or “transit deserts” that leave some communities with few transportation options. Climate change could amplify inequities as people with higher dependence on certain kinds of transportation or with limited mobility options lack alternatives if their primary mode is disrupted. Investments in climate adaptation and resilience present opportunities to improve transportation access and mobility while addressing the consequences of past decisions.

#### 10.3.1 Description of evidence

There are acknowledged transportation system inequities in New York State that pose challenges such as access to jobs.^98^, ^102^, ^309^ Because transportation systems can be impaired by extreme weather events, especially flooding,^68^ existing inequities may be exacerbated in the face of climate change. Scientific articles, institutional reports, and other published documentation of climate events agree that climate change has and will continue to affect vulnerable populations, including those who live near transportation infrastructure,^185^, ^186^ those with already limited mobility options,^12^ and those who experience disproportionate transportation hazards.^310^ Climate risks are expected to affect transportation systems and exacerbate inequities for many of the state's vulnerable communities by making it more difficult to earn a living and to access daily necessities and health care services. Inequities in human safety are also expected to increase, including the ability to evacuate during a climate event. Documented inequities that could be exacerbated in a changing climate include disproportionate transportation dependencies among low‐wage workers,^52^, ^53^ rural residents,^55^ and mobility‐impaired individuals.^57^ The literature also documents a large body of varied adaptation strategies that are being developed and implemented for every transportation mode and are considering harm reduction and transportation equity issues.^82^, ^108^, ^129^, ^146^, ^201^, ^243^, ^253^

#### 10.3.2 New information and remaining uncertainties

Changing travel demand (e.g., reduced or increased demand, or demand shift among modes) remains an uncertainty in projecting the results of efforts to reduce inequality through climate adaptation and resilience. More broadly, the net effect of climate impacts and climate adaptation measures on vulnerable populations is too uncertain to project quantitatively.

#### 10.3.3 Assessment of confidence

There is **high** confidence that transportation‐related inequities could be exacerbated by climate change impacts in New York State in the decades ahead. The assessment team found ample evidence and wide agreement that transportation‐related inequities exist within New York State, but confidence is only high (not very high) because the likelihood of these inequities being exacerbated in the future is based on observed patterns and intuition more than quantitative projections. There is also **high** confidence that planning for climate adaptation can be a vehicle for addressing such inequities, though sources vary on proposed and enacted solutions and the extent of their impact.

### 10.4 Key Finding 4

**Transportation investments increasingly seek to address climate change impacts, but decision‐makers still face constraints that limit their ability to respond proactively**. As climate impacts on the transportation system become more apparent, many transportation agencies in New York State have taken steps to design for the climate of the future, not the climate of the past, and embrace new techniques and technologies. Still, decision‐makers must contend with rising costs, financial constraints, limited personnel capacity (particularly in smaller or less well‐funded jurisdictions), and a backlog of routine maintenance needs and infrastructure in disrepair. Sustaining proactive investments requires transportation agencies and departments to balance their resource commitments against the potential costs of inaction.

#### 10.4.1 Description of evidence

The Fifth National Climate Assessment cites a wide range of evidence to underscore the importance of incorporating climate projections, adaptation, and resilience into transportation planning to reduce future risks.^24^ Reports and studies have shown that both the private and public sectors are investing in transportation system adaptation, though they face substantial constraints.^130^, ^165^, ^199^, ^208^, ^242^, ^249^, ^307^ For example, there is evidence that local spending on roads in New York has decreased relative to other competing priorities.^21^ Assertions about financial and personnel capacity constraints are supported by anecdotal evidence and intuition, including the observation that many of the most robust programmatic advancements profiled in this chapter—like NYSDOT's asset management efforts and PANYNJ's resilience guidelines for capital projects—are the work of large agencies with in‐house resilience specialists and the resources to devote to such efforts, in contrast to the smaller jurisdictions that manage the majority of New York State's roads. Multiple transportation professionals who provided input to the assessment team noted that adaptation and resilience resources can sometimes be difficult for practitioners to identify, locate, and understand. Nevertheless, transportation agencies and departments have taken steps to ensure resilience in the face of climate impacts, including measures taken in New York State, particularly since Superstorm Sandy.^69^, ^78^, ^82^, ^113^, ^115^, ^117^, ^118^, ^121^, ^147^, ^200^, ^201^, ^242^, ^247^ Additionally, there is substantial evidence in reports, published research, and news stories of enacted adaptation policies for a broad range of transportation systems in published plans and guidelines.^29^, ^69^, ^71^, ^165^, ^199^, ^200^, ^243^ Most of the citations noted here are specific to New York State.

#### 10.4.2 New information and remaining uncertainties

This assessment focuses primarily on impacts and is not intended to be an exhaustive analysis of the status of transportation system adaptation across New York State. Thus, the assertion that adaptation is underway in New York can only be taken as a qualitative observation and does not represent a quantitative characterization of any “adaptation deficit” or lack thereof. Moreover, the extent of constraints that decision‐makers face is uncertain, as the nature of various constraints changes over time and from one jurisdiction to another.

#### 10.4.3 Assessment of confidence

There is **very high** confidence that investments in transportation adaptation are occurring in New York State, based on the broad range of climate adaptation plans and related guidance by transportation authorities, as well as actual implementation, particularly in the aftermath of Superstorm Sandy. There is also **very high** confidence that decision‐makers still face impediments to proactive planning, as substantial bodies of research and empirical evidence illustrate the challenge of balancing proactive investment with funding, legal, and other constraints, while highlighting the risks of climate‐reactive stances. Publicly available data reinforce the breadth of the maintenance backlog that New York State transportation agencies and departments face, as exemplified by the number of roads and bridges rated to be in poor condition.

### 10.5 Key Finding 5

**Advancements in transportation planning, systems, and mobility present both opportunities and challenges for climate adaptation**. EVs, on‐demand mobility options, intelligent systems, and advanced materials are among the many advancements fostering the evolution of the transportation sector. While these advancements may present opportunities for enhanced mobility and environmental benefits, they can also exacerbate vulnerabilities, such as increasing dependence on the electric power grid. Planners must consider how climate change will affect the transportation systems of tomorrow, not just the systems of today.

#### 10.5.1 Description of evidence

There is abundant evidence of advancement in the transportation sector in New York State and beyond in terms of planning,^71^ system enhancement,^311^ mobility improvements,^312^ and technological change.^114^ Sources from gray literature, news stories, and scientific research document the broad range of evolutions in the sector.^59^, ^204^, ^313^, ^314^, ^315^ It is widely acknowledged that some of these innovations serve to reduce transportation's carbon footprint, thus offering a climate benefit. Examples include EVs (especially when powered by zero‐emissions electricity) and innovations that encourage greater use of mass transit, micromobility, and biking or walking instead of driving. Some innovations can also help to improve resilience. For example, the NYSDOT Transportation Systems Management and Operations Strategic Plan introduced in Section 3.4 discusses the use of vehicle‐based weather data collection to inform weather‐responsive traffic management decisions in real time.^69^ New forms of micromobility could also be considered as innovations for resilience to the extent that they give people more alternatives when other modes of transportation are disrupted. Other examples of adaptive innovations include cool pavement materials,^112^ onboard energy storage for electric trains,^114^ and artificial intelligence and data analytics to optimize port operations and vessel movements.^204^

However, studies also show that advanced transportation methods and technologies, including adaptation strategies, may underperform as projected climatic changes exceed design parameters,^316^ strain existing systems and vulnerabilities,^317^ or present problematic consequences upon deployment.^318^ For example, there is an intuitive risk inherent in making mobility more dependent on the electric power and telecommunications grids, which themselves are vulnerable to outages resulting from increasingly severe weather and climate events.^42^, ^43^, ^44^ Primary transportation infrastructure is inhibited where communities are singularly dependent on that mode of transportation and lack the opportunity or the means to transfer to another suitable mode. Evidence of planning to mitigate potential inequities and unforeseen consequences from deployed transportation advancements exists in various studies and reports.^75^, ^296^

#### 10.5.2 New information and remaining uncertainties

Advancements in the transportation sector continue to develop globally as new technology is introduced and implemented. Much research is still underway to develop new technologies and to understand their potential consequences, both positive and negative. Thus, this assessment does not attempt to comprehensively forecast future technology trends.

#### 10.5.3 Assessment of confidence

There is **very high** confidence that the evolution of the transportation system presents a mix of benefits and challenges from the perspective of climate change. Many sources suggest the potential of various advancements in the sector that can both reduce greenhouse gas emissions and build resilience. However, growing bodies of research continue to discern examples of earlier deployed advancements in transportation that led to unforeseen consequences where they have interacted with changing climate conditions, and further caution against overly optimistic assumptions about the projected benefits of current and future advancements.

## AUTHOR CONTRIBUTIONS

A.M.: Revising and editing the manuscript; review; general supervision. J.M.: Revising and editing the manuscript; review; general supervision. T.S.: Drafting, revising, and editing the manuscript; manuscript compilation and review; general supervision. H.C.: Drafting, revising, and editing the manuscript. J.C.: Drafting, revising, and editing the manuscript. C.C.: Drafting, revising, and editing the manuscript. P.D.: Drafting, revising, and editing the manuscript. A.C.R.: Drafting, revising, and editing the manuscript; manuscript compilation and review.

## COMPETING INTERESTS

The authors declare no competing interests.

### PEER REVIEW

The peer review history for this article is available at: https://publons.com/publon/10.1111/nyas.15198
